# Effectiveness of Pelvic Floor Muscle and Education-based Therapies on Bladder, Bowel, Vaginal, Sexual, Psychological Function, Quality of Life, and Pelvic Floor Muscle Function in Females Treated for Breast Cancer: A Systematic Review

**DOI:** 10.1007/s11912-024-01633-3

**Published:** 2025-01-29

**Authors:** Marie-Pierre Cyr, Tamara Jones, Udari N. Colombage, Helena C. Frawley

**Affiliations:** 1 School of Health and Rehabilitation Sciences, The University of Queensland, Brisbane, QLD Australia; 2 Melbourne School of Psychological Sciences, The University of Melbourne, Melbourne, VIC Australia; 3 Department of Physiotherapy, Monash University, Melbourne, VIC Australia; 4 Melbourne School of Health Sciences, The University of Melbourne, The Royal Women’s Hospital, Melbourne; Mercy Hospital for Women, Melbourne, VIC Australia

**Keywords:** Conservative treatment, Education, Breast cancer, Pelvic floor, Rehabilitation, Women’s health

## Abstract

**Purpose of review:**

Breast malignancy is the most common cancer in females. Symptoms of pelvic floor disorders and sexual dysfunction secondary to systemic cancer treatment may occur. Non-surgical, non-pharmaceutical conservative therapies, namely pelvic floor muscle (PFM) and education-based therapies, could be beneficial to reduce these symptoms in this population. This systematic review aimed to examine the evidence regarding their effectiveness on bladder, bowel, vaginal, sexual, psychological function, quality of life, and PFM function in breast cancer populations.

**Recent findings:**

Six databases were searched to identify interventional studies on the effect of PFM therapies, education-based therapies, or combined (multimodal) therapies on any outcome of interest. The search yielded 603 results, from which 12 studies were included. Of these, six (50%) were RCTs, one (8%) was a non-RCT with two groups, and five (42%) were non-RCTs with a single group. Findings suggest that PFM therapies (active) may be beneficial, and education in the format of CBT may improve bladder function. No data were found for bowel function and results from two RCTs were inconclusive to draw conclusions for vaginal function. Sexual function was the most frequently reported outcome. PFM therapies (active > passive) may be beneficial, and education is more likely than not to improve sexual function. For psychological function, PFM therapies (active + passive) may be beneficial, and education is more unlikely than likely to improve psychological function. For quality of life, PFM therapies (active + passive) may be beneficial, and education is more unlikely than likely to improve quality of life, although CBT combined with physical exercise may provide further improvement. PFM therapies (active ± passive) may improve PFM function. Given the limited number of studies and their methodological limitations, caution should be exercised when interpreting these study results. More research is needed to confirm findings and to investigate the clinical value of PFM therapies and combined, multimodal therapies for breast cancer populations.

**Summary:**

Non-surgical, non-pharmaceutical conservative therapies may be helpful for breast cancer populations. Clinicians should consider the highest level of available evidence to guide their practice and use their clinical judgement to select the treatment components and appropriate dosages.

**Supplementary Information:**

The online version contains supplementary material available at 10.1007/s11912-024-01633-3.

## Introduction

Breast malignancy is the most common cancer, accounting for 24–25% of cancer cases in females, with nearly 2.3 million of new cases annually worldwide [1]. Symptoms of pelvic floor disorders are prevalent in this population, with a prevalence of 38% for urinary incontinence and 18% for fecal incontinence [2]. Symptoms of sexual dysfunction are also common, with a prevalence reaching up to 70% [3–5]. Breast cancer treatment generally consists of surgery, radiotherapy, systemic therapy (e.g., endocrine therapy or chemotherapy), or a combination of these. Endocrine therapy and chemotherapy have been proposed to induce indirect changes in pelvic floor tissues by hormonal suppression or failure [6, 7]. These changes could contribute to the onset of new or the aggravation of preexisting symptoms of pelvic floor disorders and sexual dysfunction. Furthermore, there are reports of increased burden of hypoestrogenic side effects in females after breast cancer treatment compared to those who undergo natural menopause, due to the rapidity and severity of symptom onset following iatrogenic menopause [8].

Pelvic floor muscle (PFM) training has been suggested to increase blood flow and improve PFM function [9], which could mitigate the adverse effects of systemic breast cancer treatment, including symptoms of pelvic floor disorders and sexual dysfunction. A recent systematic review on conservative therapies in gynecological cancer populations concluded that combined, multimodal therapies, i.e., PFM therapies in conjunction with education-based therapies, can be effective in improving vaginal, overall pelvic floor, sexual, and PFM function [10]. These conclusions may not be generalizable to breast cancer populations as the underlying mechanisms of symptoms of pelvic floor disorders or sexual dysfunction differ between both populations. Gynecological cancer populations, for instance, may undergo pelvic surgery or radiation therapy. No study has systematically and comprehensively reviewed the evidence of the effectiveness of PFM and education-based therapies in breast cancer populations. Given that the effects of conservative therapies may extend beyond the target tissue or psychosocial domain that was the focus of the intervention [10], examining the evidence of the effectiveness of PFM and education-based therapies across a wide spectrum of outcomes that affect breast cancer females is critical to inform future research directions and clinical practice.

The objective of this systematic review was to examine the evidence regarding the effectiveness of non-surgical, non-pharmaceutical conservative therapies, namely PFM and education-based therapies, on bladder, bowel, vaginal, sexual, psychological function, quality of life, and PFM function in breast cancer populations.

## Materials and Methods

### General Methodology and Search Strategy

This systematic review adhered to the guidelines of the Preferred Reporting Items for Systematic Reviews and Meta-Analyses (PRISMA) [11] and the Synthesis Without Meta-analysis (SWiM) Reporting Guideline [12]. Informed by one prior review [10] and in collaboration with a medical librarian, we created a search strategy based on PICO eligibility criteria. We searched six databases (Medline, Embase, CINAHL, Cochrane Library, PsycINFO, and Emcare) from their respective inception dates to December 2023, without imposing restrictions on publication dates. The search aimed to identify studies of PFM and education-based therapies in breast cancer populations. The database search strategy is shown in Supplementary Information 1. References cited in included studies were considered for inclusion. For screening, if a report was not retrievable, we attempted to contact the authors for access to the report.

### Study Selection

Any original, quantitative, prospective study was included, i.e., randomized controlled trials (RCTs), non-randomized controlled trials (non-RCTs), interventional cohort or case series (pre-post) studies, and pilot studies, except case studies. Papers published in English or French in peer-reviewed journals were eligible. Following the PICOT approach (P: population, I: intervention, C: comparison, O: outcome, T: time frame; timing of delivery), the eligibility criteria were: (P) at least 75% of the participants were adult females (aged ≥ 18 years) who had either been diagnosed with or undergone any treatment for breast cancer, i.e., ductal carcinoma in situ or invasive breast cancer, or a majority of participants were adult females who had been diagnosed with or undergone any treatment for any type of breast cancer; (I) to be grouped as: a) ‘PFM therapies’; b) ‘education-based therapies’, or c) ‘combined therapies’. ‘PFM therapies’ include non-surgical, non-pharmaceutical interventions that target the pelvic floor soft tissues (e.g., performance of voluntary activation and relaxation of PFM, electrical or magnetic stimulation, manual therapy, dilator therapy, or desensitization techniques). ‘Education-based therapies’ include non-surgical, non-pharmaceutical interventions that target cognitive aspects related to the pelvic floor. Therapies had to have a plausible explanation that they could indirectly influence the function of the pelvic floor (i.e., bladder, bowel, vaginal, or muscle function). Counselling, psychoeducation, cognitive-behavioral therapy (CBT), or mindfulness-based therapy, etc. were eligible if they provided information about the pelvic floor structure, function, or exercise; addressed emotional, psychological, behavioral, or sexual aspects related to the cancer or cancer treatment repercussions, e.g., advice for vaginal lubricant, moisturizer, or body scanning. ‘Combined therapies’ refers to any intervention combining these two types of therapies. If any other treatment components were part of the intervention, they were included (e.g., general exercise) unless a pharmaceutical ingredient or laser therapy was used. Interventions were considered for eligibility regardless of the setting (e.g., individual or group, in person or remotely) or healthcare provider [10]; (C) with or without any type of comparator group; (O) any outcome related to bladder, bowel, vaginal, sexual, psychological function, quality of life, or PFM function. These can be assessed through patient-reported outcome measures, clinician-reported outcome measures, and instrument-measured outcomes; and (T) outcomes assessed before and after the intervention, at any follow-up timepoint, corresponding to either immediately after or within two weeks post-intervention (short-term follow-up), beyond two weeks but less than 12 months post-intervention (medium-term follow-up), or 12 months or longer post-intervention (long-term follow-up). Interventions delivered at any timepoint in relation to cancer treatment were included, i.e., before cancer treatment (prehabilitation), during cancer treatment, after cancer treatment (rehabilitation), or over more than one cancer treatment time phase (mixed). One reviewer (MPC or UC) independently selected the studies by screening titles and abstracts, followed by full texts. Two reviewers (HF and TJ) verified the eligibility of included studies. Any disagreement between the reviewers for selection of studies was solved through team discussion.

### Data Extraction

Two reviewers (reviewer 1: MPC or UC, and reviewer 2: TJ) independently conducted data extraction using a customized sheet, which was developed, and pilot tested by the team (MPC, HF, UC, and TJ). Extracted data included the following: study details, population characteristics, intervention arms – the treatment group (TG) intervention and the comparator group (CG), outcomes as listed in the eligibility criteria and their associated outcome measures or tools, adverse events, assessment timepoints, and results of between-group comparisons for RCTs and non-RCTs with two groups or results of within-group comparisons for non-RCTs with single group, derived from intention-to-treat or per protocol analysis if the former was unavailable. In cases where the data were non-interpretable, we attempted to contact the authors for clarification. The Template for Intervention Description and Replication (TIDieR) checklist [13] was used to retrieve information about TG interventions. Any discrepancy between reviewers for data extraction was solved through team discussion.

### Risk of Bias and Quality Assessment

The risk of bias and quality assessment of included studies was assessed independently by two reviewers (reviewer 1: MPC or UC, and reviewer 2: TJ). The Cochrane risk-of-bias (RoB) tool was used for RCTs, and the classification of risk of bias per domain (i.e., ‘low risk’, ‘unclear risk’ or ‘high risk’) was performed in accordance with the Cochrane Collaboration’s tool [14]. The Newcastle – Ottawa Scale (NOS) for non-RCTs with two groups [15], and the Joanna Briggs Institute (JBI) Critical Appraisal Checklist for Quasi-Experimental Studies for non-RCTS with a single group [16]. The percentage of agreement between reviewers was calculated. Any disagreement between the reviewers for risk of bias and quality assessment were solved through team discussion.

### Data Interpretation and Synthesis

The PFM therapies were analyzed and classified based on their description in the included studies, and they were grouped for interpretation to reduce heterogeneity. These therapies were labeled ‘active’ if they involved voluntary control of PFM activation by the individual; ‘passive’ if they involved no voluntary control of PFM activation by the individual but rather techniques from external sources aimed at changing pelvic floor tissues, such as manual therapy, dilator therapy, desensitization techniques, direct application of vaginal lubricant, moisturizer; or ‘electrostimulation’ if they involved electrical current to stimulate PFM activity. Assessment timepoints were categorized into either ‘baseline’ for the initial assessment before intervention, ‘follow-up timepoint’ for subsequent assessments, or ‘end timepoint’ when there was only one subsequent assessment, or when explicitly identified as such by the authors in cases of multiple follow-up assessments. To facilitate interpretation of individual study results, data values along with their significance, or narrative descriptions, were used to indicate the ‘direction of findings’. Considerations for interpretation were summarized to offer further insight to the reader. Among these, studies were assigned a TIDieR checklist score, where each item described in the checklist within the paper was given a point of ‘1’. The maximum achievable score was 12, indicating that the description of the TG intervention fulfilled all checklist items.

This systematic review used a summary narrative synthesis approach and thematic categorization to enable a thorough examination of the available evidence [12]. Findings are organized according to each outcome of interest, including bladder, bowel, vaginal, sexual, psychological function, quality of life, and PFM function. In addition, the Oxford Centre for Evidence-Based Medicine (OCEBM) 2011 Levels of Evidence framework was employed to rate the level of evidence for each outcome [17]. Findings are grouped according to the level of evidence derived from the study design, the type of TG intervention, and the population (breast or mixed), which contributed to a comprehensive interpretation.

## Results

### Selected Studies

**Fig. 1:**
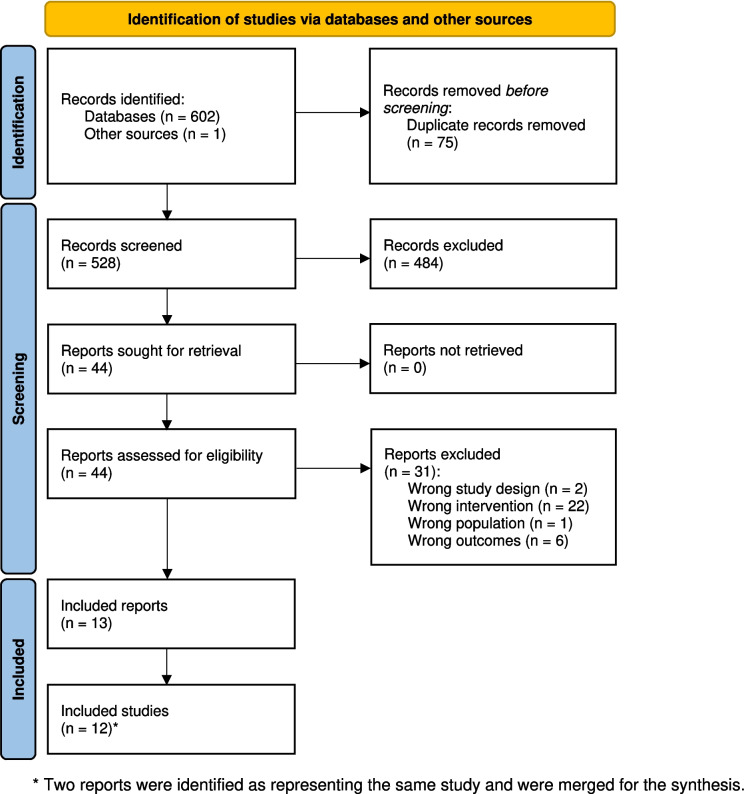
PRISMA Flow Chart

The flow chart of this systematic review is presented in Fig. 1. A total of 603 records were identified and screened. After screening titles and abstracts, 44 full texts were assessed for eligibility. Thirteen full texts were included, representing 12 original, individual studies. Six studies were RCTs, of which five were conducted in breast cancer populations (*n* = 1063 females) and one in a mixed cancer population (*n* = 58 females, 47 with breast and 11 with gynecological cancer). One non-RCT with two groups was conducted in a breast cancer population (*n* = 80 females). Five studies were non-RCTs with single group conducted in breast cancer populations (*n* = 161 females).

Table 1 shows the characteristics of included studies. Studies are presented according to the population of interest, i.e., breast (*n* = 11 studies) and mixed (*n* = 1 study) cancer populations, study design (RCTs followed by non-RCTs), type of TG intervention (combined therapies followed by PFM and education-based therapies), arranged from the most recent to the oldest studies. The age—average (years)—of participants ranged from 39 to 59 in breast cancer populations. Eleven (92%) studies examined a rehabilitation intervention (after cancer treatment), one (8%) a mixed intervention (delivery over more than one cancer treatment time phase), and none examined a prehabilitation intervention (before cancer treatment). Investigated TG interventions were the following: one (8%) combined therapies (PFM + education-based therapies), eight (67%) education-based therapies (+ other), and three (25%) PFM therapies (+ other). The full description of TG interventions, as detailed by the authors and inserted into the TIDieR checklist, is provided in Supplementary Information 2. This includes scores indicating the extent to which checklist items have been met. The number of items from the TIDieR checklist used to describe therapies delivered to the TG ranged between 4–11 (out of 12), corresponding to approximately 33%−92% of the total checklist items. Based on the available assessment timepoints in included studies, the most reported follow-up time frame related to short- and medium-term effectiveness, and two studies (17%) provided results for long-term effectiveness. Studies reported intervention effect on bladder (*n* = 4, 33%), vaginal (*n* = 2, 17%), sexual (*n* = 11, 92%), psychological (*n* = 6, 50%) function, quality of life (*n* = 5, 42%), and PFM function (*n* = 3, 25%). No study investigated effects on bowel function.

**Table 1:** Characteristics of Included Studies

Authors; year; country	Study design; sample size	Type of cancer, n (%)	Age, years Mean (SD)	Category of intervention for timing of delivery; time elapsed since cancer diagnosis or last cancer treatment to pre-intervention (baseline) assessment	Treatment Group (TG) details: type; name; short description	Comparator Group (CG) name (short description)	Assessment timepoints; follow-up time frame	Reported outcomes
Advani et al. [18]; 2017; United States	RCT; *n* = 57	Breast, 57 (100%)	59 (8)	Rehabilitation; Time since cancer treatment: NR	**PFM therapy (passive) + education (+ usual care)**; Combination of sexual counselling, vaginal moisturizers, lubricants, dilation, and PFM exercises; Access to a website providing detailed help with women’s cancer-related sexual problems and study booklet, plus 6 × 15–30-min coaching calls during the treatment period and a follow-up call at 3 months. Participants were instructed to use vaginal moisturizer daily during week 1, and then 2–3 times weekly, and advised to have penetrative sex with a partner and/or to use the dilator at least 2 times per week with water-based vaginal lubricant. Duration of therapy: 12 weeks and 6 months of supply for moisturizers	Usual care (study booklet including self-help strategies and resources for problems with arthralgia, vaginal dryness and pain during sex, hot flashes, and loss of bone density)	Baseline: pre-intervention; Follow-up timepoint 1: 6 months post-intervention; Follow-up timepoint 2: 12 months post-intervention; Medium- and long-term follow-up	Vaginal function; Sexual function
Fatehi et al. [19]; 2019; Iran	RCT; *n* = 118	Breast, 118 (100%)	44–45 (7)	Rehabilitation; Time since cancer treatment: mean 10–11 (SD 4–5) in months	**Education**; Psychosexual counselling; 6 × 90–120-min counselling sessions (one per week) involving education on the anatomy of sexual organs and sexual cycles for the gender of both spouses, the consequences of breast cancer and its treatment effects on sexual relationships and the relationship with the spouse and providing solutions based on Master and Johnson’s sex therapy principles to deal with sexual problems. Duration of therapy: 6 weeks	Wait-list (no intervention)	Baseline: pre-intervention; Follow-up timepoint 1: immediately post-intervention; Follow-up timepoint 2; 3 months post-intervention; Short- and medium-term follow-up	Sexual function
Hummel et al. [20, 21]; 2017; 2018; Netherlands	RCT; *n* = 169	Breast, 169 (100%)	51 (7)	Rehabilitation; Time since cancer treatment: NR; Time since cancer diagnosis: mean 38 (SD 16) in months	**Education**; Internet-based cognitive behavioral therapy (CBT); Approx. 20 weekly sessions to be completed within the intervention period. The therapist and client formulated therapeutic goals and selected 4–5 modules (4–8 interventions per module) that suited the sexual problems best. Each intervention consisted of standardized information texts, homework assignments, a report to the sexologist, and feedback from the sexologist. Sessions consisted of an extensive reply (feedback, additional questions, and remarks) from the therapist in response to the completed homework assignment(s) via email. Duration of therapy: 20–24 weeks	Wait-list (information booklet addressing sexuality issues after cancer treatment)	Baseline: pre-intervention; Follow-up timepoint 1 (not reported in this review): mid-intervention (10 weeks after the start); Follow-up timepoint 2: post-intervention; Follow-up timepoint 3: 3 months post-intervention (for TG only); Follow-up timepoint 4: 9 months post-intervention (for TG only); Short- and medium-term follow-up	Sexual function; Psychological function; Quality of life
Duijts et al. [22]; 2012; Netherlands	RCT; *n* = 422	Breast, 422 (100%)	48 (6)	Rehabilitation; Time since cancer treatment: NR	**Education ± physical exercise**; (1) CBT (+ relaxation exercises); (2) physical exercise (aerobic); (3) CBT (+ relaxation exercises) + physical exercise; The CBT involved 6 × 90-min group sessions including relaxation exercises with a booster program 6-week after completion (primary focus was hot flashes and night sweats, but other symptoms were also addressed) The physical exercise group completed an individually tailored, home-based, self-directed, exercise program of 2.5–3 h per week. Each woman was provided with a heart-rate monitor and was instructed in its use to achieve a target heart rate (60–80% intensity using the Karvonen formula) in an appropriate form of aerobic exercise, supported by 2 telephone interviews with a physiotherapist, and a final session during which they received physical activity advice. The CBT (+ relaxation exercises) + physical exercise group completed both intervention components concurrently. Duration of therapy: 12 weeks	Wait-list (usual care)	Baseline; pre-intervention; Follow-up timepoint 1: post-intervention; Follow-up timepoint 2: 3 months post-intervention; Short- and medium-term follow-up	Bladder function; Sexual function; Psychological function; Quality of life
Schover et al. [23]; 2011; United States	RCT; *n* = 297	Breast, 297 (100%)	54 (10)	Rehabilitation; Time since cancer treatment: NR	**Education**; Sisters Peer Counseling in reproductive issues after treatment program; Workbook and 3 × 60–90 -min in-person peer counselling sessions. Duration of therapy: 6 weeks	Workbook + optional telephone peer counseling of less than 30-min	Baseline: pre-intervention; Follow-up timepoint 1: immediately post-intervention; Follow-up timepoint 2: 6 months post-intervention; Follow-up timepoint 3: 12 months post-intervention; Short-, medium- and long-term follow-up	Bladder function; Vaginal function; Sexual function; Psychological function; Quality of life
Schover et al. [24]; 2013; United States	RCT; *n* = 58	Breast, 47 (81%) Gynecological, 11 (19%)	53 (9)	Rehabilitation; Time since cancer treatment: NR; Time since cancer diagnosis: mean 3.5 (SD 4) in years	**Education**; Internet-based intervention for cancer-related sexual problems + 3 supplemental in-person counselling sessions; All participants received access to the *Tendrils* website that provided information on sexual information and concerns, vaginal dryness and pain, and body image. Half the sample also received 3 supplemental in-person counselling sessions. Duration of therapy: 12 weeks	Internet-based intervention for cancer-related sexual problems	Baseline: pre-intervention; Follow-up timepoint 1: immediately post-intervention; Follow-up timepoint 2: 3 months post-intervention; Follow-up timepoint 3: 6 months post-intervention; Short- and medium-term follow-up	Sexual function; Psychological function; Quality of life
Zangeneh et al. [25]; 2023; Iran	Non-RCT; *n* = 80	Breast, 80 (100%)	41–42 (5–6)	Unclear (likely mixed); Time since cancer diagnosis/treatment: NR	**Education**; Sexual education based on the Ex‑PLISSIT model: permission (P), limited information (LI), specific suggestions (SS), and intensive therapy (IT), including relaxation techniques, breathing techniques, PFM exercise); 4 × 60–90-min education sessions (one per week). Based on the four stages of the Ex‑PLISSIT model, the education sessions included measures to solve common sexual problems after cancer treatment, relaxation techniques, breathing exercises, PFM exercises, and managing body image. Duration of therapy: 4 weeks	Routine care	Baseline: pre-intervention; End timepoint: immediately post-intervention; Short-term follow-up	Sexual function
Alfarra et al. [26]; 2022; Saudi Arabia	Non-RCT; *n* = 30	Breast, 30 (100%)	46 (range 28–65)	Rehabilitation; Time since cancer treatment: NR	**PFM therapy (active + passive) + yoga**; Supervised pelvic floor rehabilitation (which included PFM training, seven yoga poses, manual therapy, and dilator education); 8 × 45-min sessions (one per week), including 30 min of exercise (including PFM exercises and yoga poses: mountain pose, tree pose, standing forward bend pose, warrior pose, bridge pose, bound angle pose, and seated twist pose), 10-min manual therapy for PFM, and 5-min dilator education. Dilator use was encouraged at home 3–4 times per week. Duration of therapy: 8 weeks	NA	Baseline: pre-intervention; End timepoint: 8 weeks post-intervention; Medium-term follow-up	Bladder function; Sexual function; PFM function
Colombage et al. [27]; 2023; Australia	Non-RCT; *n* = 54	Breast, 54 (100%)	50 (7)	Rehabilitation; Time since cancer treatment: NR; Time since cancer diagnosis: mean 4.5 (SD 3) in years	**PFM therapy (active)**; Telehealth PFM training; 8 × PFM training telehealth sessions using the femfit® intra-vaginal pressure biofeedback device. Participants completed 3 sets of 6–10 maximal contractions, 6–10 fast contractions, 3 podium (endurance) contractions and 3 “knack” contractions per session. Participants were instructed to complete the home PFM training program 5 times per week using the femfit® phone application. Duration of therapy: 12 weeks	NA	Baseline: pre-intervention; End timepoint: immediately post-intervention; Short-term follow-up	Bladder function; PFM function
Juraskova et al. [28]; 2013; Australia	Non-RCT; *n* = 25	Breast, 25 (100%)	51 (8)	Rehabilitation; Time since cancer treatment: NR	**PFM therapy (active + passive)**; Olive oil, vaginal exercise, and moisturizer; 4 appointments with a physiotherapist who taught techniques for penetration to achieve PFM relaxation, and PFM relaxation exercises which involved 5 repetitions of gently contracting, holding, and relaxing the PFMs. Participants were also provided with Replens® and organic olive oil and instructed to apply Replens® 3 times per week, perform PFM relaxation exercises 2 times per day, and use olive oil during intercourse. Duration of therapy: 26 weeks	NA	Baseline: pre-intervention; Follow-up timepoint 1: mid-intervention (at week 4); Follow-up timepoint 2: mid-intervention (at week 12); Follow-up timepoint 3: end of intervention (at week 26) (likely immediately post-intervention) Short-term follow-up	Sexual function; Psychological function; Quality of life; PFM function
Bokaie et al. [29]; 2022; Iran	Non-RCT; *n* = 32	Breast, 32 (100%)	40 (8)	Rehabilitation; Time since cancer treatment: mean 3.5 (SD 3) in years	**Education**; Group counselling based on a problem-solving solution; 8 × 90-min sessions (one per week), including dedicating time and space to oneself, relaxation skills, sensory exercises, breathing training, joint massage, and PFM exercising and sharing it with one’s spouse. Duration of therapy: 8 weeks	NA	Baseline: pre-intervention; Follow-up timepoint 1: immediately post-intervention; Follow-up timepoint 2: 1 month post-intervention; Short- and medium-term follow-up	Sexual function
Bober et al. [30]; 2020; United States	Non-RCT; *n* = 20	Breast, 20 (100%)	39 (7)	Rehabilitation; Time since cancer treatment: 85% at 1 month or less and 15% at 2–3 months post-cancer treatment	**Education**; Sexual health and rehabilitation after ovarian suppression treatment; 1 × 4-h group session and telephone booster at 1-month. The group session comprised four modules: education about sexual health rehabilitation; experiential exercises in body awareness (including PFM relaxation); structured mindfulness based cognitive exercises, and; individual goal planning, with actionable steps to be reviewed during the telephone booster. Duration of therapy: 4 h	NA	Baseline: pre-intervention; End timepoint: 2 months post-intervention; Medium-term follow-up	Sexual function; Psychological function

*CBT* cognitive-behavioral therapy, *CG* comparator group, *NA* not applicable, *NR* not reported, *PFM* pelvic floor muscle, *Q1* first quartile, *Q3* third quartile, *RCT* randomized controlled trial, *SD* standard deviation, *TG* treatment group

### Risk of Bias and Quality Assessment

Table 2, Table 3, and Table 4 show the rating on the risk of bias or quality assessment tool of all included studies. The overall risk of bias was assessed as ‘some concerns’ for RCTs, ‘poor quality’ for the non-RCT with two groups, and the percentage of satisfied criteria on the JBI checklist for non-RCTs with single group ranged from 43 and 86%. The agreement between reviewers for all scored items was 87%.

**Table 2:**
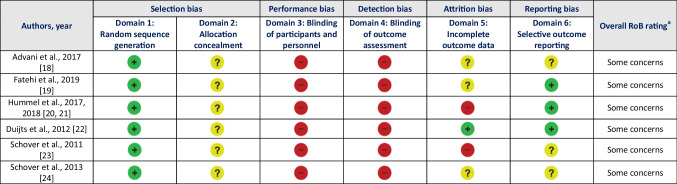
RoB Domains of Reviewed RCTs

*RCT* randomized controlled trial, *RoB* risk of bias

Legend:

Low risk of bias

Unclear risk of bias

High risk of bias

^a^ ‘Low risk overall’: domains 1 and 2 are low risk, domain 3 is low or unclear risk, domains 4 to 6 are low risk. ‘Some concerns’: all other combinations. ‘High risk overall’: domains 1 and 2 are high risk.

**Table 3:** NOS Items of Reviewed Non-RCTs with Two Groups

Authors, year	Selection (0–4 stars)				Comparability (0–2 stars)	Outcome (0–3 stars)			Overall quality rating
	Representativeness of the exposed cohort	Selection of the non-exposed cohort	Ascertainment of exposure	Demonstration that outcome of interest was not present at start of study	Comparability of cohorts on the basis of the design or analysis	Assessment of outcome	Long enough follow-up for outcomes to occur	Adequacy of follow-up of cohorts	
Zangeneh et al., 2023 [25]	**□**	**□**	-	**□**	**□**	-	**□**	-	Poor quality

*NOS* Newcastle – Ottawa Scale, *RCT* randomized controlled trial

Legend:

**□**Criterion satisfied

-Criterion not satisfied: high risk of bias

**Table 4:** JBI Checklist for Quasi-Experimental Study Items of Reviewed Non-RCTs with Single Group

Authors, year	Is it clear what is the ‘cause’ and what is the ‘effect’ (i.e., there is no confusion about which variable comes first)?	Were the participants included in any comparisons similar?	Were the participants in the comparison receiving similar treatment/care other than the exposure or intervention of interest?	Was there a control group?	Were there multiple measurements of the outcome both pre and post the intervention/exposure?	Were follow- up complete and, if not, were differences between groups in terms of their follow-up adequately described?	Were the outcomes of participants included in any comparisons measured in the same way?	Were outcomes measured in a reliable way?	Was appropriate statistical analysis used?	Number of satisfied criteria among applicable criteria (n satisfied/n applicable)
Alfarra et al., 2022 [26]	Yes	NA	NA	No	Yes	Unclear	Yes	No	No	3/7
Colombage et al., 2023 [27]	Yes	NA	NA	No	Yes	Yes	Yes	Yes	Yes	6/7
Juraskova et al., 2013 [28]	Yes	NA	NA	No	Yes	Yes	Yes	Yes	Yes	6/7
Bokaie et al., 2022 [29]	Yes	NA	NA	No	Yes	No	Yes	Yes	Yes	5/7
Bober et al., 2020 [30]	Yes	NA	NA	No	Yes	Yes	Yes	Yes	Yes	6/7

*JBI* Joanna Briggs Institute, *NA* not applicable, *RCT* randomized controlled trial

### Outcomes

Findings of individual studies according to bladder, vaginal, sexual, psychological function, quality of life, and PFM function, along with considerations for interpretation, are presented in Supplementary Information 3. Only three studies out of the 12 included studies (25%) reported data on adverse events related to the TG intervention. No adverse events were found for an intervention including PFM training, yoga poses, manual therapy, and dilator use [26], and an internet-based intervention with in-person counselling sessions [24]. Two adverse events, i.e., itching in the pubic area and abdominal cramping, were mentioned in a study on PFM therapy, although the authors described that they were unlikely to have been caused by the intra-vaginal pressure biofeedback device or the performance of PFM exercises [27].

#### Bladder Function

Two RCTs provided data on bladder function in breast cancer populations [22, 23]. One RCT had four arms (*n* = 422), with three different interventions, i.e., CBT (+ relaxation exercises), physical exercise (aerobic), and CBT (+ relaxation exercises) + physical exercise, which were compared with usual care [22]. Interventions of interest (CBT and CBT + physical exercise) improved bladder function (BFLUTS), with the strongest effect shown at 3-months post-intervention follow-up from the CBT alone arm [22]; although the sample size calculation was based on the primary outcome of endocrine symptoms. However, the study did not achieve the a priori calculated sample size for the follow-up timepoints [22]. One RCT (*n* = 297) compared two types of an education intervention and found no between-group differences for bladder function which was assessed using a 5-point subscale (Breast Cancer Prevention Trial Symptom Checklist) [23]; however, no sample size calculation was provided, no primary outcome was specified, and no data were provided. It is also possible that the similarities between the intervention arms and the potentially low treatment dosage prevented the detection of significant between-group differences (TG: workbook + three counselling sessions vs CG: workbook and optional counselling sessions initiated by participant) [23].

Two studies in which PFM therapies were provided to breast cancer populations reported results of single-group pre-post comparisons, and both studies observed positive results [26, 27] despite neither being powered for bladder outcomes. One study (*n* = 54) investigated prevalence and burden (frequency, severity, and impact) of urinary incontinence (ICIQ-UI SF) and found an improvement in all outcomes following a PFM therapy (active) intervention delivered by physiotherapy-supervised telehealth and supported with home-based biofeedback [27]. The other study (*n* = 30) examined a PFM therapy (active + passive) + yoga intervention and reported a significant improvement in bladder function (UDI-6) in 80% of participants [26].

#### Vaginal Function

One RCT provided data on vaginal function in a breast cancer population (*n* = 57) [18]. No improvement was found following a PFM therapy (passive) + education intervention compared with usual care on vaginal irritation (BESS—gynecologic irritation subscale) and vaginal pH [18]; however, dosage of therapy was unclear, no data were provided, and adherence to the intervention was not documented. A larger RCT (*n* = 297) compared two types of an education intervention and assessed vaginal irritation on a subscale (Breast Cancer Prevention Trial Symptom Checklist) [23], but results were not described.

#### Sexual Function

Five RCTs reported data on sexual function in breast cancer populations, of which three provided education-based therapies [19–21, 23], one provided CBT (+ relaxation exercises) and CBT (+ relaxation exercises) + physical exercise [22], and one provided PFM therapy (passive) + education [18]. Two out of the three former RCTs, with sample sizes of *n* = 118 [19] and *n* = 169 [20, 21], found an improvement in the overall sexual function (FSFI) after psychosexual counselling [19] and internet-based CBT [20, 21] compared with wait-list; the latter study listed sexual function outcomes as primary and was powered for these outcomes [20, 21]. The study with the largest sample size (*n* = 297) did not measure a change in sexual function [23]. Although this study could have been underpowered for this outcome, similarities between the intervention arms and the potentially low treatment dosage, as mentioned previously for this study in the bladder outcome section, may not have allowed the detection of differences [23]. Based on FSFI subscales and other scales (SAQ, LSSQ, SQOL-F, FSDS-R) that were used in these studies [19–21, 23], education-based therapies had no effect on sexual satisfaction [19–21], and data are very limited to draw conclusions for other sexual function outcomes. The RCT (*n* = 422) that provided CBT + physical exercise demonstrated more positive results for sexual habit and pleasure (SAQ) for CBT alone [22]; although sexual function was not the primary outcome (endocrine symptoms were) and the study was not designed to demonstrate the superiority of one intervention over another. The RCT on PFM therapy (passive) + education (*n* = 57) designated overall sexual function (FSFI) as the primary outcome, however found no between-group differences on this outcome, nor sexual interest (MSIQ) and dyspareunia (BESS), but showed a reduction in sexual distress (FSDS-R) [18]. The dosage for dilator therapy was unclear and no data on participant adherence to the intervention were reported [18]. An RCT in a mixed cancer population (*n* = 58) reported an improvement in overall sexual function (FSFI), in addition to sexual interest (MSIQ) when in-person counselling sessions were added to an internet-based intervention [24]; however, data were not presented and it is unclear if the study was powered for these outcomes, even though sexual function (FSFI) was identified as the primary outcome.

In contrast to results of RCTs on education-based therapies described above, a recent non-RCT with two groups and a smaller sample size (*n* = 80) did not find an improvement in sexual function (FSFI) but did observe an improvement in sexual satisfaction (LSSQ) following education sessions which included information on PFM exercises [25]. This study included in the between-group comparison only those who attended all sessions [25], corresponding to a per-protocol analysis rather than an intention-to-treat analysis as used in previous RCTs, which likely contributed to larger differences between groups. Four studies presented the findings of single-group pre-post comparisons, of which two provided education-based therapies [29, 30] and two provided PFM therapies (active + passive) [26, 28]. All studies found an improvement in overall sexual function (FSFI); although studies had relatively small sample sizes (*n* = 32 for [29], *n* = 20 for [30], *n* = 25 for [28], and *n* = 30 for [26]), no study was powered for sexual function outcomes, and only one indicated sexual function as the primary outcome [26]. Data as measured by FSFI subscales and other scales (VAS-DYS, SAQ, LSSQ) were limited or inconsistent to draw further conclusions [28–30].

#### Psychological Function

Four RCTs assessed education-based therapies [20–24], of which one was conducted in a mixed cancer population [24]. From these studies, one compared internet-based CBT with wait-list (*n* = 169) [20, 21], one compared fixed counselling sessions in addition to a workbook with optional counselling sessions and the workbook (*n* = 297) [23], one measured the additional benefits of in-person counselling sessions when added to an internet-based intervention (*n* = 58) [24], and one investigated CBT and CBT + physical exercise (*n* = 422) [22]. Although results from the latter were non-interpretable [22], the other three RCTs found no improvement in psychological function (HADS, BSI-18) following education-based therapies [20, 21, 23, 24]; although no study identified psychological function as the primary outcome, and one provided a sample size calculation but for another outcome [20, 21].

Two studies reported results of single-group pre-post comparisons [28, 30]. One study (*n* = 25) involved PFM therapy (active + passive) and showed improvement in anxiety but not in depression (HADS) [28]. The other study (*n* = 20) involved an education-based therapy and showed improvement in anxiety and global severity but not in somatization and depression (BSI-18) [30]. However, these studies were small, neither was powered for these outcomes, and no primary outcome was specified.

#### Quality of Life

The four RCTs on education-based therapies that evaluated psychological function also assessed quality of life [20–24], and included a cancer-specific quality of life measure (FACT-ES, FACIT-Sp, QLACS, FACT-B vs SF-36). Three RCTs observed no improvement compared to CG [20, 21, 23, 24]; no study had quality of life as the primary outcome, and none were powered for this outcome. The RCT comparing wait-list to CBT and CBT + physical exercise (*n* = 422) [22] reported mixed findings, with improvements observed for physical functioning, vitality, mental, and endocrine subscales (short-term) and endocrine subscale (medium-term) for both TGs, and CBT + physical exercise provided further improvements on emotional (short- and medium-term) and mental subscales (medium-term) [22]. No effect on bodily pain was observed [22]. The primary outcome of this study was endocrine symptoms, and a sample size calculation was provided [22].

One single-group study assessed the effect of a PFM therapy (active + passive) on quality of life (FACT-B, FACT-ES) [28]. Improvements were observed in all quality of life outcomes [28]; although the sample size was small (*n* = 25), no sample size calculation was described, and no primary outcome was specified.

#### PFM Function

Three studies in breast cancer populations reported results of single-group pre-post comparisons [26–28]. All interventions were PFM therapies, all with an active component [26–28], two with an additional passive component [26, 28], and one also included yoga poses [26]. The two studies that measured PFM strength using vaginal palpation [26] and a manometer [27] appear to suggest an improvement in this parameter; although samples were of medium sizes (*n* = 30 [26] and *n* = 54 [27]), and no studies were powered for this outcome. Despite the fact that the results are unclear and no sample size calculation was provided, one study (*n* = 25) reported a potential reduction in intra-vaginal pressure at rest and during a relaxation task following a PFM therapy (active + passive); however, no changes in PFM activity using surface intra-vaginal electromyography were observed [28].

### Evidence Summary

Table 5 presents the summary of the findings with level of evidence [17] and recommendations for each outcome.

**Table 5:** Summary of Findings with Level of Evidence and Recommendations

Outcome	Summary; level of evidence; recommendations	
	Breast cancer populations	Mixed cancer populations
Bladder function	• **PFM (active or active + passive) therapies** o May improve bladder function (**level of evidence: 4 from 2 single-group trials**) • **Education-based therapies** o CBT may improve bladder function (**level of evidence: 2 from 1 RCT**); education-based therapies may not improve urinary incontinence (**level of evidence: 2 from 1 RCT**)	**No studies**
Vaginal function	• **PFM (passive) therapy + education** o May not improve vaginal function (**level of evidence:** **2 from 1 RCT**)	**No studies**
Sexual function	• **PFM (passive) + education therapies** o May not improve overall sexual function; may improve sexual distress (**level of evidence: 2 from 1 RCT**) • **PFM (active + passive) therapies** o May improve overall sexual function (**level of evidence:** **4 from 2 single-group trials**) • **Education-based therapies** o May (**level of evidence: 2 from 3 RCTs and 4 from 2 single-group trials**) or may not (**level of evidence: 2 and 3 from 1 RCT and 1 non-RCT**) improve overall sexual function and some aspects of sexual function o May not improve sexual satisfaction unless information on PFM exercises is given (**level of evidence: 2 from 3 RCTs**)	• **Education-based therapies** o May improve overall sexual function (**level of evidence: 2 from 1 RCT**)
Psychological function	• **PFM (active + passive)** o May improve some aspects of psychological function (**level of evidence: 4 from 1 single-group trial**) • **Education-based therapies** o May not improve psychological function (**level of evidence: 2 from 2 RCTs**) or may improve some aspects of psychological function (**level of evidence: 4 from 1 single-group trial**)	• **Education-based therapies** o May not improve psychological function (**level of evidence: 2 from 1 RCT**)
Quality of life	• **PFM (active + passive)** o May improve quality of life (**level of evidence: 4 from 1 single-group trial**) • **Education-based therapies** o May not improve quality of life (**level of evidence: 2 from 3 RCTs**); may improve some domains of quality of life, with further and longer improvement when combined with physical exercise (**level of evidence: 2 from 1 RCT**)	• **Education-based therapies** o May not improve quality of life (**level of evidence: 2 from 1 RCT**)
PFM function	• **PFM (active ± passive) therapies (+ other)** o May improve PFM strength (**level of evidence:** **4 from 2 single-group trials**) • **PFM (active + passive) therapies** o May improve PFM resting tone and relaxation (**level of evidence:** **4 from 1 single-group trial**)	**No studies**

*CBT* cognitive-behavioral therapy, *PFM* pelvic floor muscle.

Oxford Centre for Evidence-Based Medicine (OCEBM) 2011 Levels of Evidence framework [17]

Level 1: ‘Systematic review of randomized trials or n-of-1 trials’.

Level 2: ‘Randomized trial or observational study with dramatic effect’.

Level 3: ‘Non-randomized controlled cohort/follow-up study’.

Level 4: ‘Case-series, case–control studies, or historically controlled studies’.

## Discussion

### Summary

This systematic review has summarized the evidence for the effectiveness of conservative therapies, namely PFM and education-based therapies, on bladder, bowel, vaginal, sexual, psychological function, quality of life, and PFM function in breast cancer populations. For bladder function, data suggest that PFM therapies (active) may be beneficial and that education-based therapies in the format of CBT may improve function. No study reported effectiveness of therapies on bowel function. For vaginal function, findings were inconclusive due to lack of data and significant issues with intervention dosage and compliance. Sexual function was the most frequently reported outcome, and data suggest that PFM therapies (active > passive) may be beneficial, and education-based therapies are more likely than not to improve sexual function, although they may not improve sexual satisfaction unless they include information on PFM exercises. For psychological function, PFM therapies (active + passive) may be beneficial, and education-based therapies are more unlikely than likely to improve function. For quality of life, PFM therapies (active + passive) may be beneficial, and education-based therapies are more unlikely than likely to improve quality of life, although combination with physical exercise may provide further improvement. Only studies on PFM therapies (active ± passive) assessed PFM function, and data suggest that they may improve PFM function (e.g., strength, resting tone, and relaxation).

### Interpretation of Results Per Outcomes

#### Pelvic Floor Outcomes (Bladder, Vaginal, PFM, and Bowel Function)

As the bladder and vagina are pelvic organs whose function is influenced by the PFMs, interventions aimed at improving bladder or vaginal function would typically target those structures [9, 31]. In this review, both RCTs [22, 23] that measured bladder function provided education-based therapy. Inconsistent findings were observed, with the larger study [22] delivering a more intensive education component reporting an improvement in urinary incontinence, although the mechanism of improvement remains unclear. Both single-group trials [26, 27] which tested PFM therapies provided encouraging results that they may improve bladder function, which is in line with the available evidence from gynecological cancer populations [10]. Data from single-group trials in breast cancer populations reported improvement in PFM function following PFM therapies [26–28], also in line with data from gynecological cancer populations [10], and could be potential treatment mechanisms. Given the paucity of data in breast cancer females, more research is needed to establish the effectiveness of conservative therapies on bladder function in this population. In this review, vaginal irritation and vaginal pH did not improve following PFM therapy (passive) + education [18]. Females treated for breast cancer may experience iatrogenic menopause and associated genitourinary syndrome which involves lower urinary tract, pelvic floor, and vulvovaginal tissue changes. These changes could increase the risk of urinary symptoms and dyspareunia in this population. Based on available data in non-cancer populations [9], it is worth considering the inclusion of a PFM therapy component, preferably an active component [10], in future RCTs that aim to improve bladder or vaginal function in breast cancer populations.

No studies on conservative therapies for bowel function were identified in this review. Bowel dysfunction may occur in 18% [32] to 24% [33] of females following breast cancer treatment and cause significant distress and impact [33]. Therefore effectiveness of PFM or education-based therapies on bowel function remains an important and under-researched area.

#### Sexual Function

Sexual function was the most frequently reported outcome in the studies included in this review, and most studies investigated education-based therapies. Across all study designs, mixed findings were reported regarding the effectiveness of these therapies to improve sexual function [19–21, 23–25, 29, 30], and they may or may not improve sexual satisfaction [19–21, 25]. This finding is in line with a Cochrane review which examined the evidence for multiple interventions targeting sexual dysfunction in females with cancer and did not find clear evidence of the impact of interventions [34]. In addition to the potential lack of statistical power which could explain this inconsistent finding, the heterogeneity in participant characteristics (e.g., age, types of breast cancer treatment, time since cancer treatment, types and severity of sexual dysfunction at baseline) and intervention content could be contributing factors. Although it is unclear if the mode of delivery could play a role in the effectiveness of education-based therapies, a few elements suggest that their effectiveness could be enhanced by a more personalized and goal-oriented intervention (rather than giving general information to all), higher dosage, higher participant compliance with recommendations, and providing more opportunities for interaction with a healthcare provider. The review of Seav et al. [35] concurs with the importance of having a specific therapeutic goal with education-based therapies. Findings of the current review differ from theirs, primarily due to the differences in study eligibility criteria. In this review, studies were included if the interventions had a plausible explanation for influencing pelvic floor function, i.e., if they targeted sexual dysfunction of a hypothesized source involving dysfunction of the pelvic floor, and regardless of the presence of sexual dysfunction at baseline.

Findings of studies investigating PFM therapies in this review were limited and with mixed results [18, 26, 28]. However, the study reporting that the intervention did not improve overall sexual function was an RCT in which PFM therapy (passive) + education was provided, with unclear dosage, and no data on adherence to the intervention was reported [18]. This is not surprising as it has been suggested that intervention dosage, adherence, and supervision are critical for optimal effectiveness in cancer populations [10]. In addition, a recent systematic review highlighted the contribution of the PFMs in sexual function/response in females, and a statistically-significant moderate association between PFM strength and sexual function was found [36]. This suggests that PFM therapy (passive) may be insufficient to improve sexual function, whereas PFM therapy (active), as used in the single-group trials which showed an improvement in sexual function, should be considered [26, 28].

Overall, interpretation of our findings underlines that sexual dysfunction in breast cancer populations may arise from multiple sources, making it critical to identify the most likely sources using a biopsychosocial lens to target them effectively with evidence-based components and appropriate dosages. Available evidence suggests that PFM therapies (active) and combined, multimodal therapies deserve further investigation in more robust and adequately powered studies [10, 26, 28, 36]. Future studies may need to be flexible, allowing for tailoring when applied to a heterogenous population to enhance effectiveness. The importance of adopting this patient-centered approach is further reinforced by the fact that sexual satisfaction is a complex construct with a definition that is unique for each individual [37].

#### Psychological Function

Based on the evidence from RCTs, education-based therapies are not effective for improving psychological function in breast cancer populations [20, 21, 23, 24]. Although TG interventions were well reported, they had potentially low or inadequate dosage and adherence. Both single-group trials reported improvement in anxiety but not in depression [28, 30]. Although these studies investigated different types of intervention (PFM therapy vs education-based therapy) and had small sample sizes, it is possible that dosage was sufficient for the included participants [28, 30]. It is worth noting that none of the studies in this review were designed or powered to specifically address psychological dysfunction, therefore it may be unsurprising that the majority, and all RCTs, found no improvement in this non-primary outcome.

#### Quality of Life

According to the evidence from three RCTs, education-based therapies are not effective for improving quality of life [20, 21, 23, 24]. Similarly, as described in the psychological function section, these studies, which also assessed psychological function and found no effect, were not designed or powered to improve quality of life. However, an improvement in several quality of life domains was observed following CBT and when CBT was combined with physical exercise provided further and longer-term effects. A recent review concluded that physical exercise improves quality of life in breast cancer populations [38], and therefore if the goal of therapy is to improve quality of life, including physical exercise as a component of conservative therapies should be considered. One single-group trial reported an improvement in quality of life following PFM therapy (active + passive), although it is unclear which domains beside endocrine symptoms were improved.

### General Observations and Recommendations for Future Research

Compared with the recent review on conservative therapies in gynecological cancer populations [10], most therapies in this review were education-based with a direct or indirect intent of improving sexual function, among other outcomes. This general direction in research is not surprising given the prevalence of sexual dysfunction in breast cancer populations and the low resource requirements to deliver education. The quality reporting of TG interventions in this review was superior on average when compared with studies in the other review [10]. However, this may be explained by the simplicity of describing a unimodal intervention, such as education-based therapies, where there are fewer elements to report. What seems critical for future studies, however, is design of an intervention that is likely to have an effect on the outcome, thorough recording and reporting of participant’s adherence to the intervention. This is essential to determine whether improvements are due to the application of these recommendations and to understand the mechanisms of effect.

This review highlighted several considerations for the design of future studies on education-based therapies, and also identified gaps in knowledge regarding PFM therapies and combined, multimodal therapies in this population. Available evidence indicates that it is worth considering PFM therapies and combined, multimodal therapies. Mechanistic and controlled studies are required to determine if this population or subgroups benefit from these therapies. Recommendations for future research are presented in the above sections per outcome. In addition, an observation that emerged from interpretation of findings related to psychological function and quality of life is that these outcomes were not the primary focus in the studies. This raises questions about their inclusion. Given that RCTs should prioritize meaningful outcomes for patients, and that quality of life is one of the most meaningful outcomes, future studies should consider include these outcomes if the study design or intervention is likely to impact these outcomes.

This review also identified several pitfalls in the design of included studies, emphasizing the importance of exercising caution when interpretating study results and the need for further research. The majority of studies had multiple outcomes without controlling for the potential bias of multiple comparisons, lacked an a priori sample size calculation, and did not identify a primary outcome. Additionally, some studies had small sample sizes, no CG, at least a moderate risk of bias, compared similar interventions, or may have included an heterogenous population who could have responded differently to therapy.

### General Recommendations for Clinical Practice

High-quality evidence, often from RCTs, should underpin clinical practice [39]. However, RCTs are costly, resource-intensive, and challenging to conduct in a short timeframe to enable rapid implementation in the clinical setting. Therefore, clinical practice should be guided by the best available evidence, including findings from non-RCTs when RCT data is lacking [40]. As previously discussed, breast cancer females often experience symptoms of sexual dysfunction and pelvic floor disorders. Although education-based therapies seem to offer advantages over PFM therapies and combined, multimodal therapies – such as versatile delivery modes for adaptability across different contexts and the possibility of involvement of various healthcare providers – evidence emphasizes that proper screening and identification of therapeutic goals are essential to determine the therapy components. Available data are encouraging for PFM therapies and combined, multimodal therapies in breast cancer populations, and these may be considered if evaluation reveals they can address the patient’s needs. Clinicians should consider personalized and goal-oriented therapies, with appropriate dosages, strategies to enhance participant adherence and opportunities for patient interaction with a healthcare provider. Given the scant knowledge to date of effectiveness of conservative therapies in this population, monitoring for adverse events is crucial, even when education only is provided, and clinicians are advised to use their clinical judgement to determine the best treatment strategy for optimal outcomes.

### Strengths and Limitations

Major strengths of this systematic review were the comprehensive search strategy, inclusion of various study designs and a wide range of outcomes. This allowed us to assign a level of evidence to the effect of different interventions on different outcomes important to patients. This approach also enabled us to identify key areas for future research and considerations for clinical practice. Two reviewers independently selected the studies, assessed the risk of bias and quality of studies, and extracted the data. The use of the TIDieR checklist ensured a standardized approach to extracting information related to the TG intervention and assessing quality reporting. Despite these strengths, some limitations warrant consideration when interpreting the findings. A meta-analysis was not feasible due to the limited number and heterogeneity of studies. While some results suggest effectiveness of specific conservative therapies in breast cancer populations, more research is required.

## Conclusions

This study is the first to review the evidence of effectiveness of PFM and education-based therapies in breast cancer populations. This review highlighted important gaps in knowledge, emphasizing the need for more studies in this field.

## Key References

- Cyr MP, Jones T, Brennen R, Colombage U, Frawley HC. Effectiveness of pelvic floor muscle and education-based therapies on bladder, bowel, vaginal, sexual, psychological function, quality of life, and pelvic floor muscle function in females treated for gynecological cancer: a systematic review. Curr Oncol Rep. 2024;26(11):1293–320.
  - This paper informed the search strategy of the current review.
- Campbell M, McKenzie JE, Sowden A, Katikireddi SV, Brennan SE, Ellis S, et al. Synthesis without meta-analysis (SWiM) in systematic reviews: reporting guideline. BMJ. 2020;368:l6890.
  - This document provides clear guidelines for synthesizing evidence narratively, enabling systematic and transparent reporting of findings and ensuring rigor and reliability of conclusions.
- Hoffmann TC, Glasziou PP, Boutron I, Milne R, Perera R, Moher D, et al. Better reporting of interventions: template for intervention description and replication (TIDieR) checklist and guide. BMJ. 2014;348:g1687.
  - This document provides a clear list of items that should be met for comprehensive and transparent reporting of interventions, facilitating replication and implementation of research findings. This process is critical for translating findings into practice.
- OCEBM Levels of Evidence Working Group. The Oxford 2011 Levels of Evidence. UK: Oxford Centre for Evidence-Based Medicine; 2011. https://www.cebm.ox.ac.uk/resources/levels-of-evidence/ocebm-levels-of-evidence.
  - This document provides a standardized framework for assessing the quality and strength of evidence across different studies, which ensures a consistent and objective evaluation of research findings, essential for drawing reliable and valid conclusions.

## Supplementary Information

Below is the link to the electronic supplementary material.

Supplementary file1 (DOCX 31 KB)

Supplementary file2 (DOCX 120 KB)

Supplementary file3 (DOCX 122 KB)

## Data Availability

No datasets were generated or analysed during the current study.
