# Supplementary material for: Effectiveness of Pelvic Floor Muscle and Education-based Therapies on Bladder, Bowel, Vaginal, Sexual, Psychological Function, Quality of Life, and Pelvic Floor Muscle Function in Females Treated for Breast Cancer: A Systematic Review
Source: Curr Oncol Rep. 2025 Jan 29;27(2):168–89. doi: 10.1007/s11912-024-01633-3 (PMC11861006; doi:10.1007/s11912-024-01633-3)
Supplement: Supplementary file 1 — Supplementary file1 (DOCX 31 KB) [file 11912_2024_1633_MOESM1_ESM.docx]

**Supplementary Information 1. Database Search Strategy.**

| **Database search strategy and related question** | **Database** | **Search terms for populations** | **Search terms for interventions** | **Other search terms** |
| --- | --- | --- | --- | --- |
| What is the evidence of the effectiveness of non-surgical, non-pharmaceutical, PFM and education-based therapies on any type of pelvic floor dysfunction in patients before, during, or after any type of treatment for any type of breast cancer? | CINAHL | “breast neoplasm*” OR (“breast” AND (“cancer*” OR “tumo#)) | “physiotherap*” OR “therap*” OR “*training” OR “exercise OR educat*” OR “dilator” OR “physical therap*” OR “rehabilitat*” OR “biofeedback” OR “conservative therapy” OR “behavio#ral therap*” OR “Kegel”  OR  ((“pain” OR “sexual” OR “psychological” OR “therapeutic” OR “psychosexual” OR “neuroscience” OR “patient”) AND “education”) OR (“behavio*” AND (“therapy” OR “intervention”)) OR “CBT” OR (“sex*” AND (“therapy” OR “rehabilitation”)) OR “mindfulness” | (“pelvic floor” OR “pelvic floor muscle”)  AND  (“urinary incontinence” OR “fecal incontinence” OR “anal incontinence” OR (“urge*” AND “urinary”) OR “genitourinary syndrome of menopause” OR “sexual dysfunction” OR “dyspareunia” OR “vaginismus” OR “pelvic organ prolapse”) |
|  | Cochrane Library | “breast neoplasm*” OR (“breast” AND (“cancer*” OR “tumo*)) | ““physiotherap*” OR “therap*” OR “*training” OR “exercise OR educat*” OR “dilator” OR “physical therap*” OR “rehabilitat*” OR “biofeedback” OR “conservative therapy” OR “behavio#ral therap*” OR “Kegel”  OR  ((“pain” OR “sexual” OR “psychological” OR “therapeutic” OR “psychosexual” OR “neuroscience” OR “patient”) AND “education”) OR (“behavio*” AND (“therapy” OR “intervention”)) OR “CBT” OR (“sex*” AND (“therapy” OR “rehabilitation”)) OR “mindfulness” | (“pelvic floor” OR “pelvic floor muscle”)  AND  (“urinary incontinence” OR “fecal incontinence” OR “anal incontinence” OR (“urge*” AND “urinary”) OR “genitourinary syndrome of menopause” OR “sexual dysfunction” OR “dyspareunia” OR “vaginismus” OR “pelvic organ prolapse”) |
|  | OVID Medline / PsycINFO / Embase / Emcare | “breast neoplasm*” OR (“breast” AND (“cancer*” OR “tumo?)) | “physiotherap*” OR “therap*” OR “*training” OR “exercise OR educat*” OR “dilator” OR “physical therap*” OR “rehabilitat*” OR “biofeedback” OR “conservative therapy” OR “behavio#ral therap*” OR “Kegel”  OR  ((“pain” OR “sexual” OR “psychological” OR “therapeutic” OR “psychosexual” OR “neuroscience” OR “patient”) AND “education”) OR (“behavio*” AND (“therapy” OR “intervention”)) OR “CBT” OR (“sex*” AND (“therapy” OR “rehabilitation”)) OR “mindfulness” | (“pelvic floor” OR “pelvic floor muscle”)  AND  (“urinary incontinence” OR “fecal incontinence” OR “anal incontinence” OR (“urge*” AND “urinary”) OR “genitourinary syndrome of menopause” OR “sexual dysfunction” OR “dyspareunia” OR “vaginismus” OR “pelvic organ prolapse”) |

PFM: pelvic floor muscle
