# Supplementary material for: Effectiveness of Pelvic Floor Muscle and Education-based Therapies on Bladder, Bowel, Vaginal, Sexual, Psychological Function, Quality of Life, and Pelvic Floor Muscle Function in Females Treated for Breast Cancer: A Systematic Review
Source: Curr Oncol Rep. 2025 Jan 29;27(2):168–89. doi: 10.1007/s11912-024-01633-3 (PMC11861006; doi:10.1007/s11912-024-01633-3)
Supplement: Supplementary file 2 — Supplementary file2 (DOCX 120 KB) [file 11912_2024_1633_MOESM2_ESM.docx]

**Supplementary Information 2. Treatment Group Interventions According to the TIDieR Checklist and as Reported by the Authors.**

(a) Summary TIDieR Checklist

| **Authors, year** | **Study design** | **Name of intervention** | **Rationale** | **Materials** | **Procedures** | **Provider** | **Mode of delivery** | **Location** | **Intervention dosage** | **Tailoring** | **Modifications** | **Planned intervention adherence & strategies for adherence** | **Actual intervention adherence** | **TIDieR score** |
| --- | --- | --- | --- | --- | --- | --- | --- | --- | --- | --- | --- | --- | --- | --- |
| **Breast cancer populations: 5 RCTs, 1 non-RCT with 2 groups, 5 non-RCTs with single group** | | | | | | | | | | | | | | |
| Advani et al., 2017 | RCT | 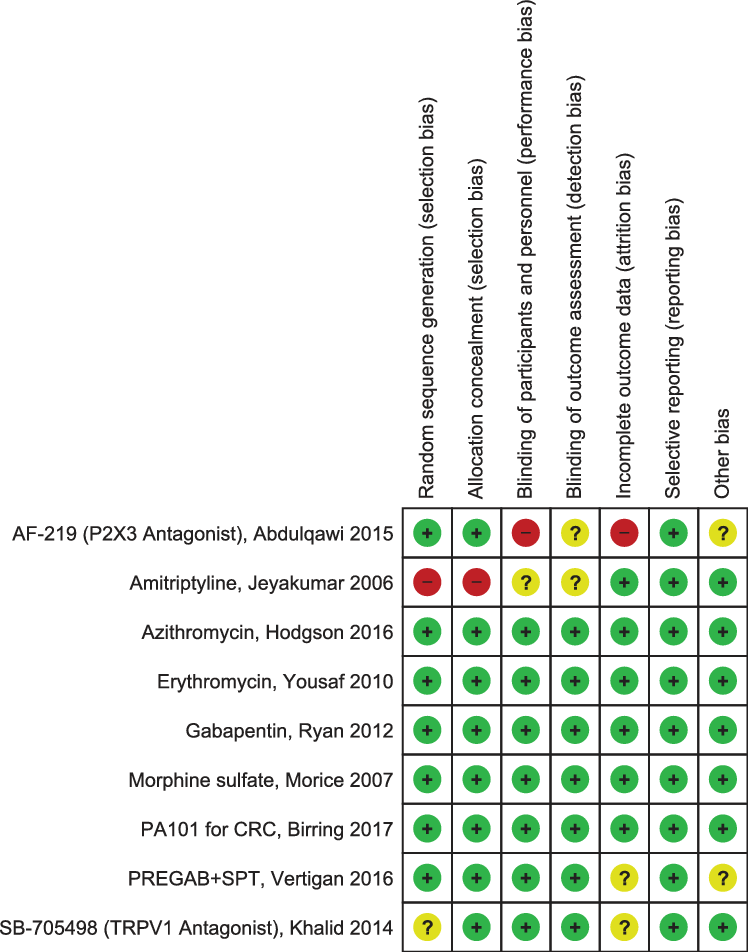 | 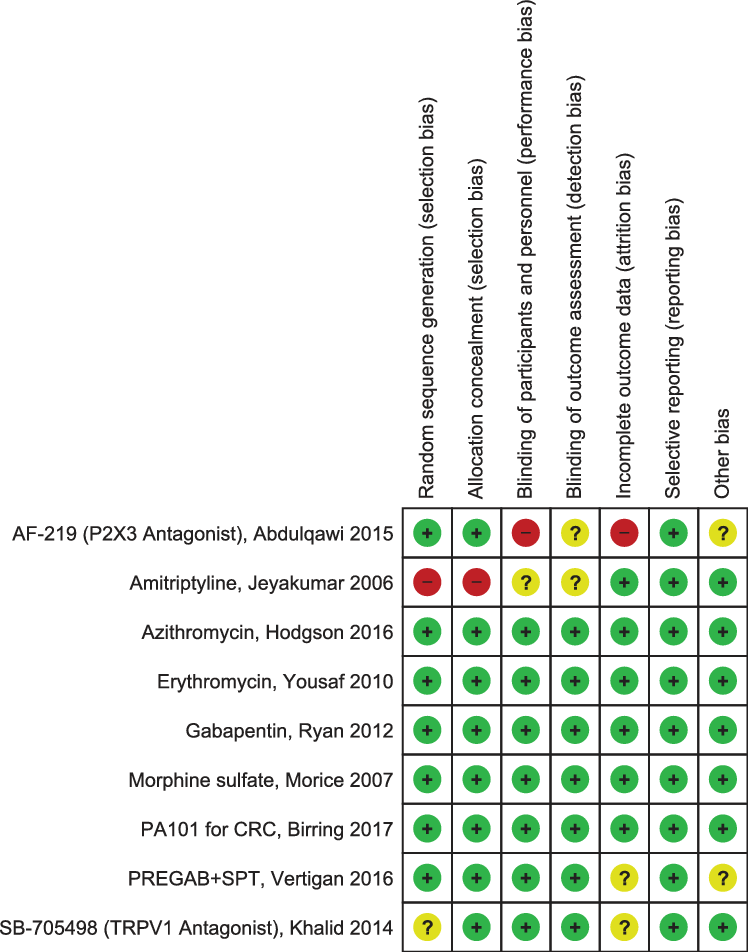 | 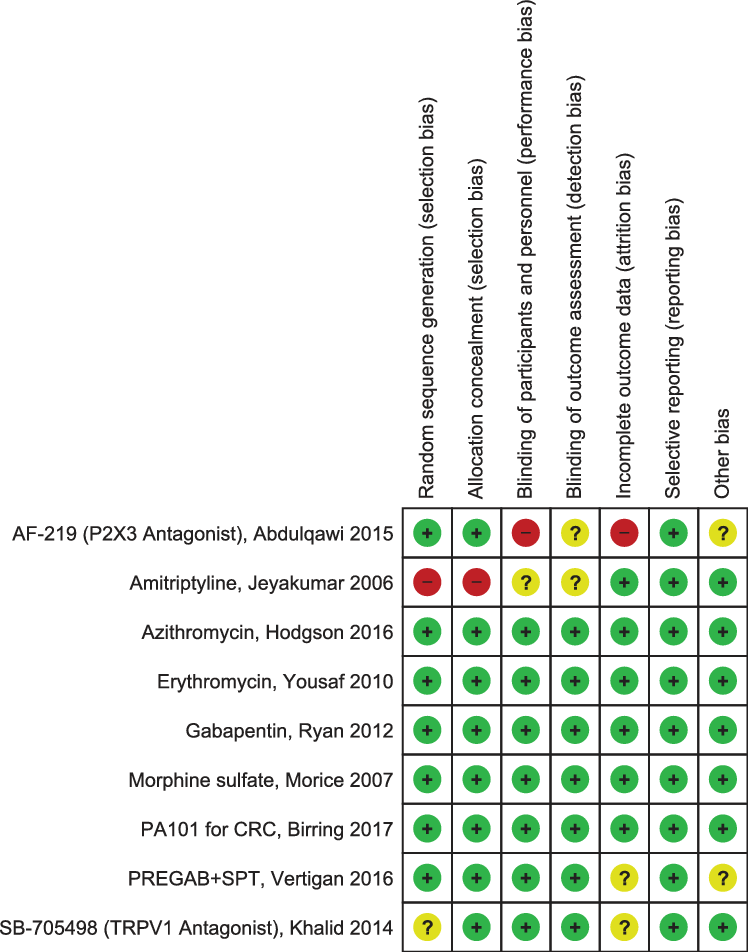 | 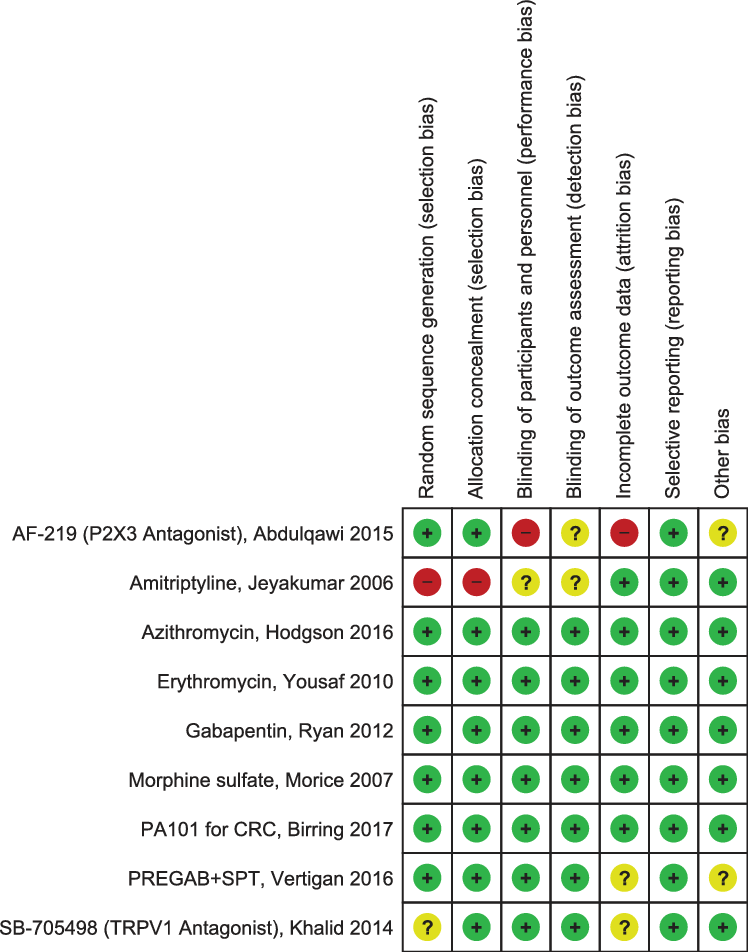 | 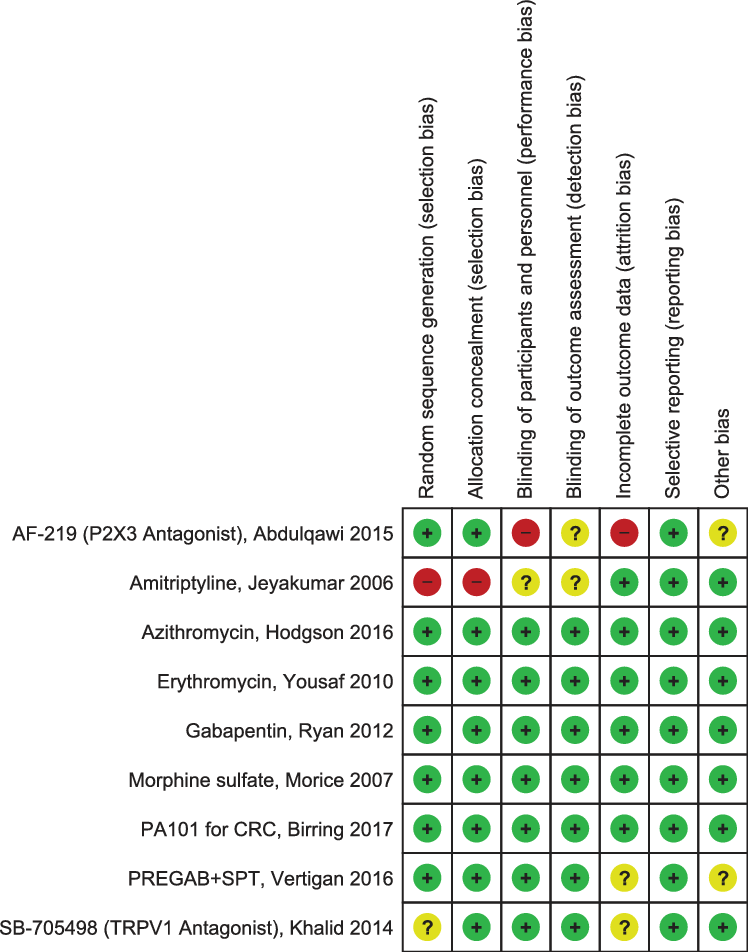 | 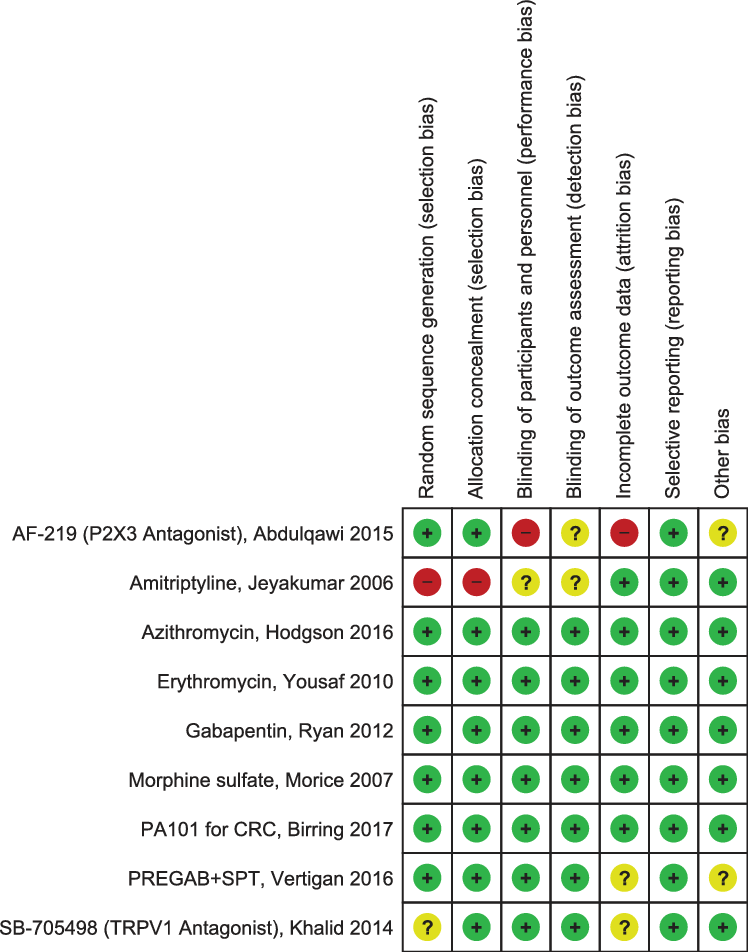 | 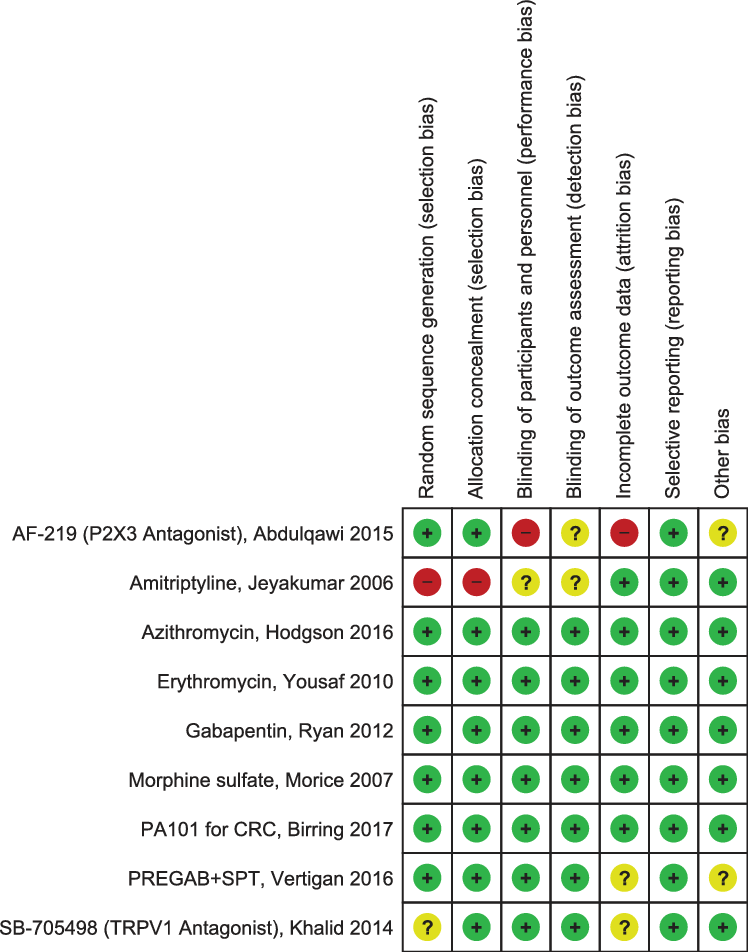 | 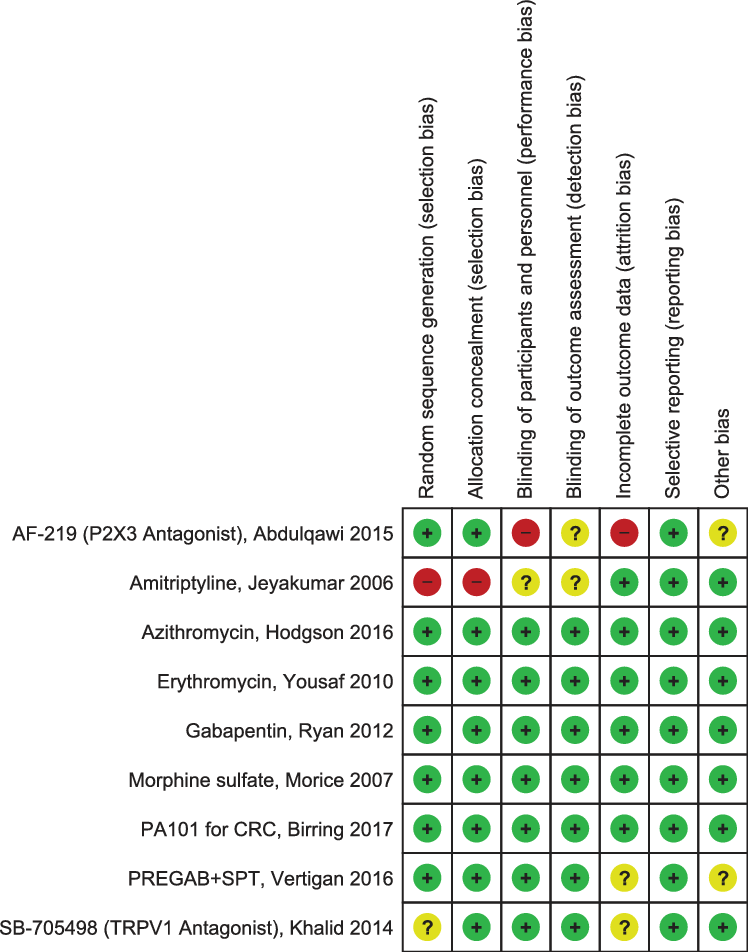 | 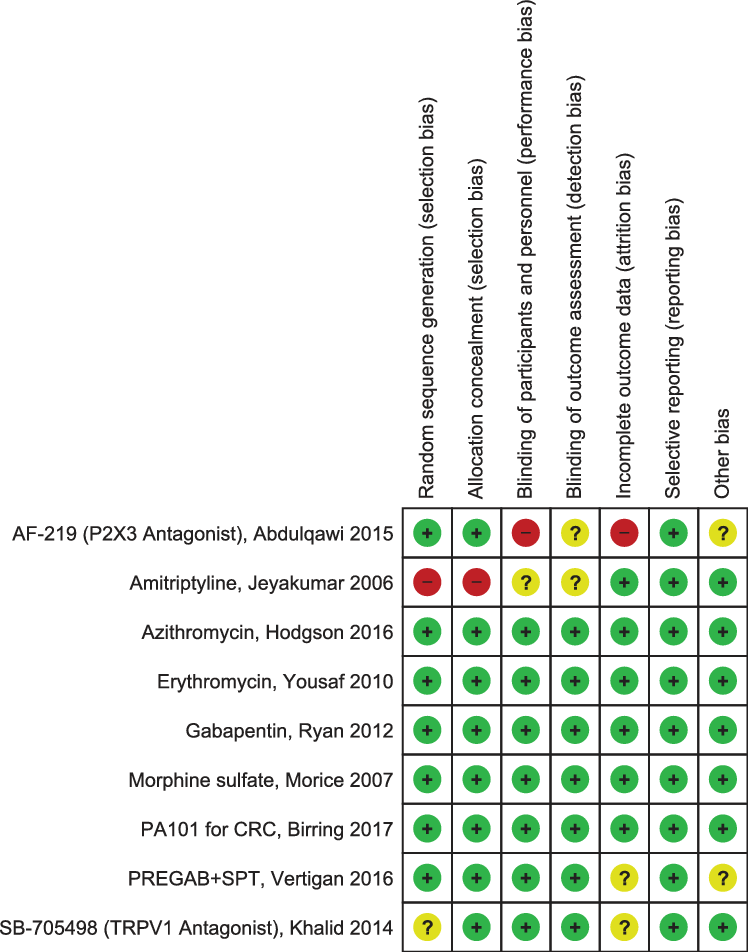 | 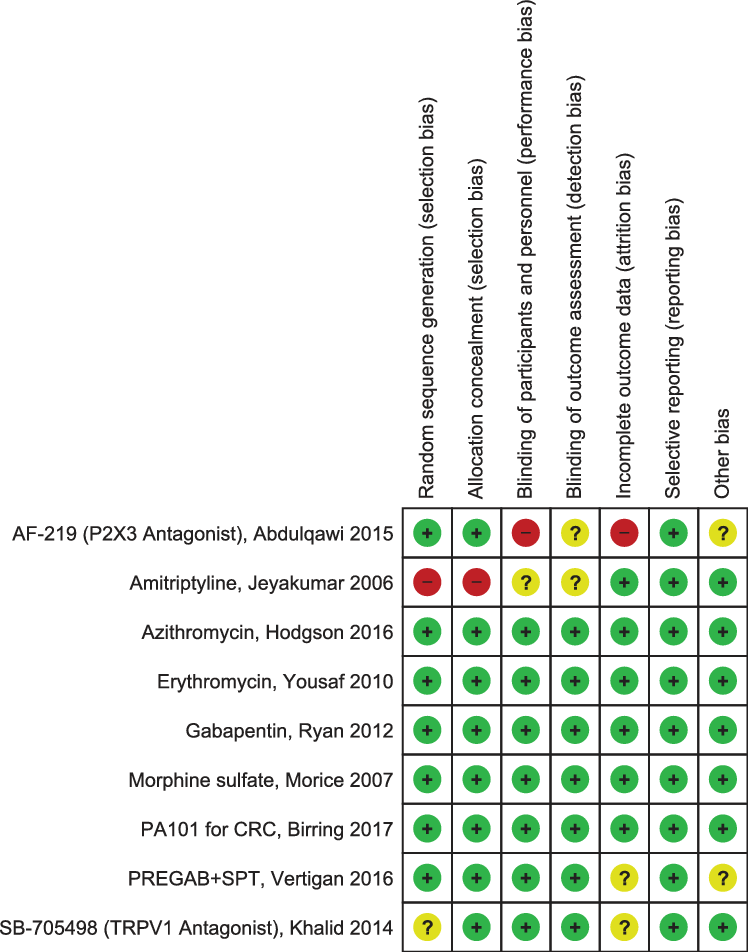 | 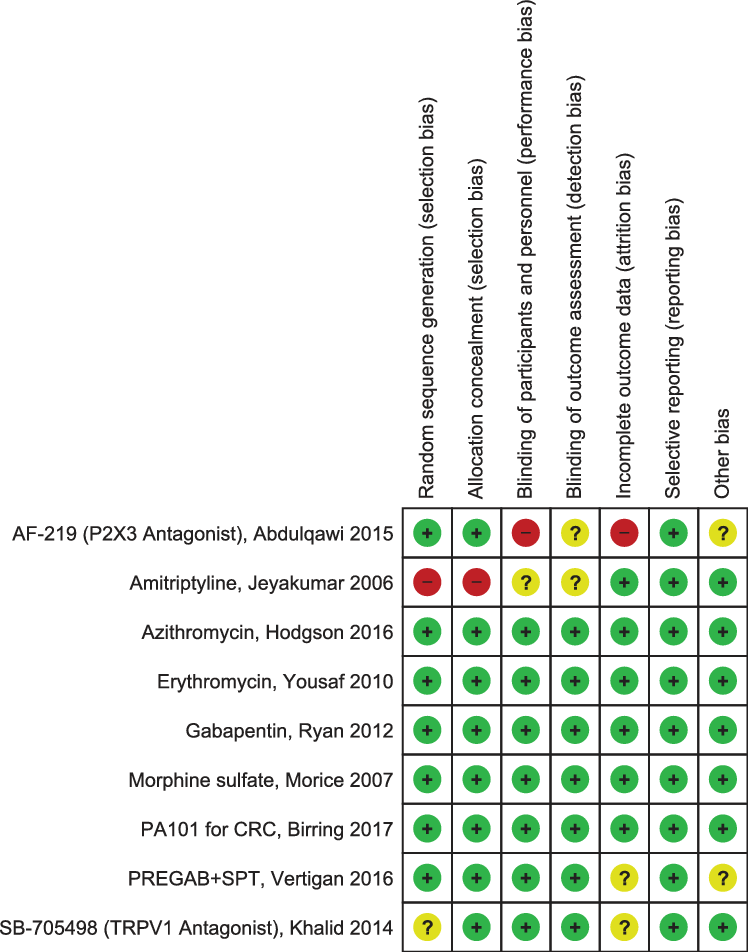 | 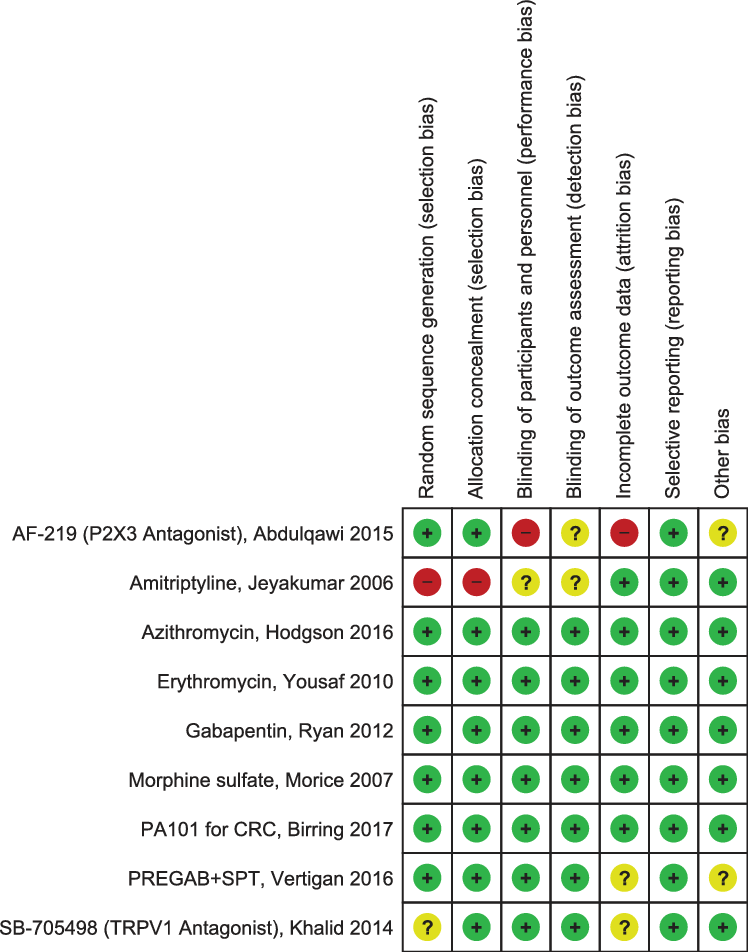 | 9 |
| Fatehi et al., 2019 | RCT | 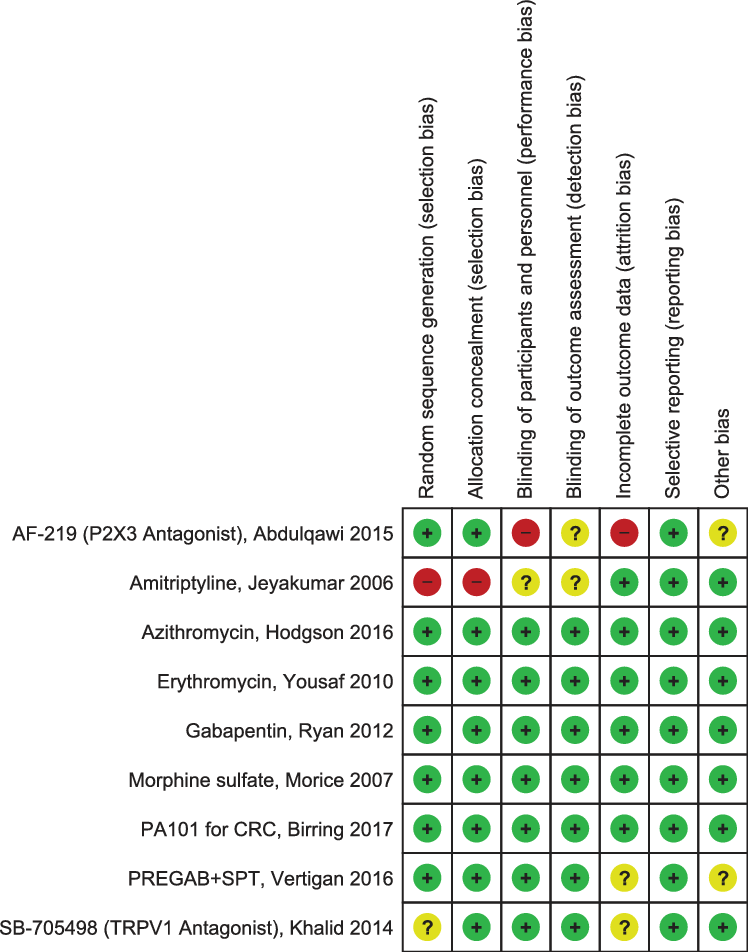 | 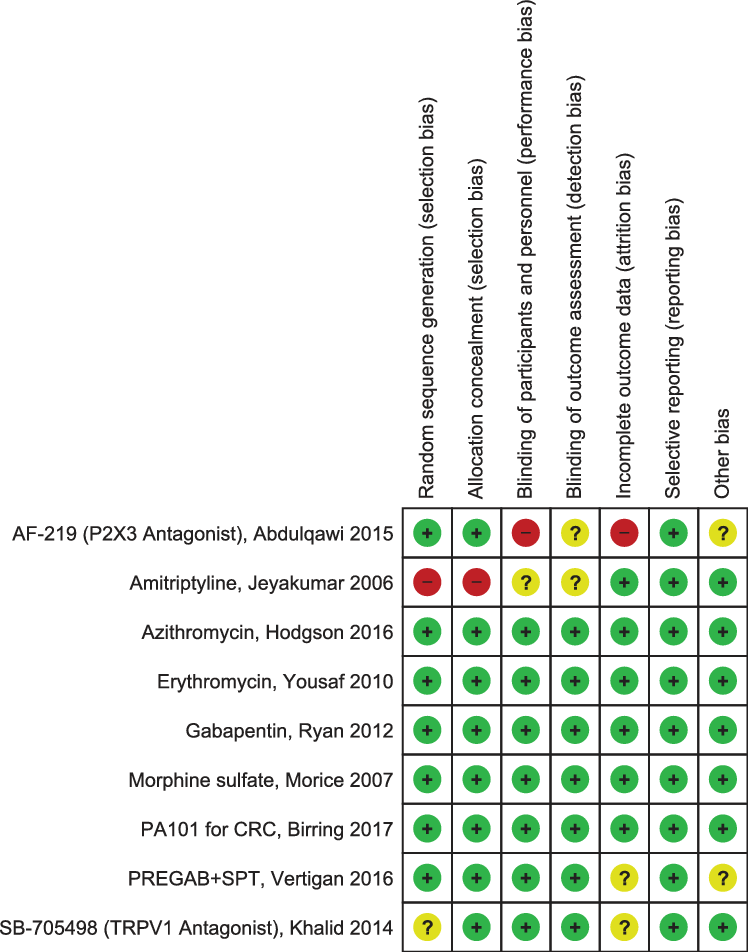 | 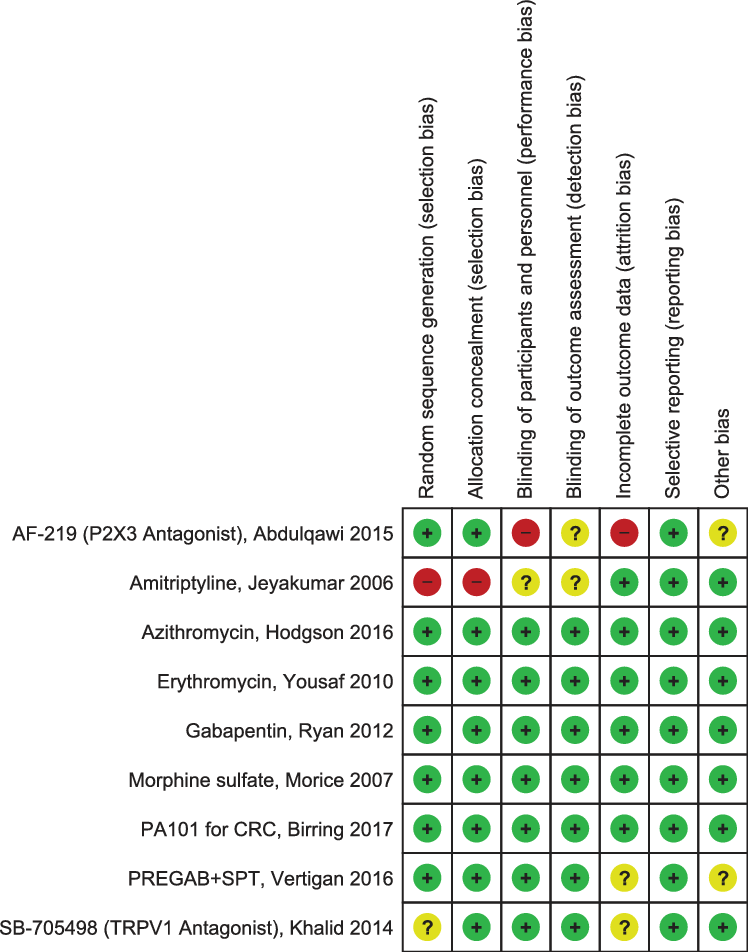 | 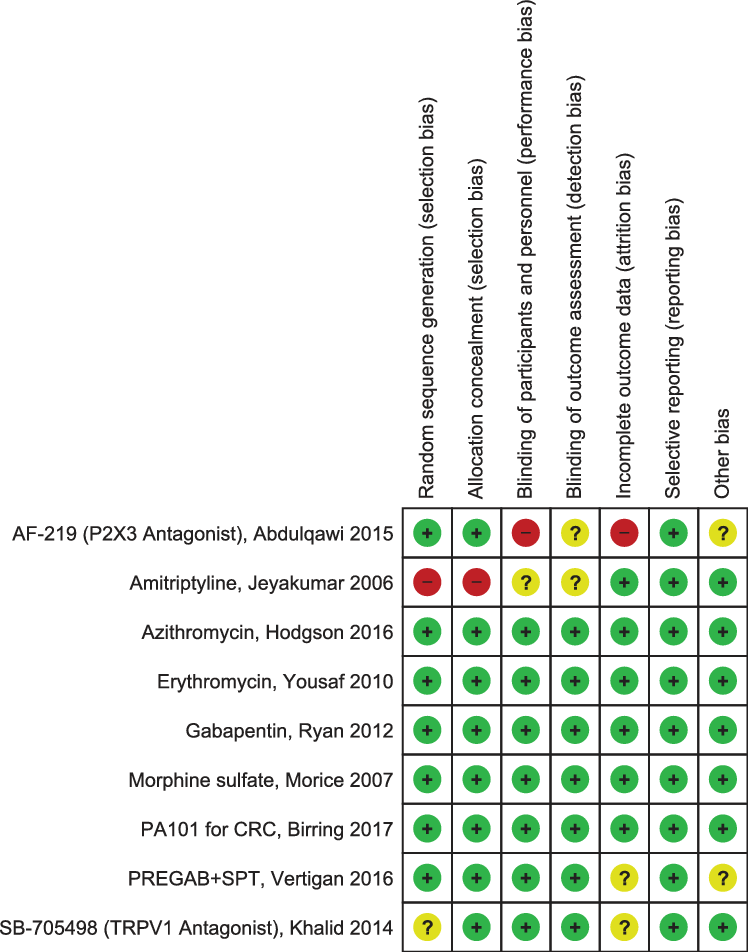 | 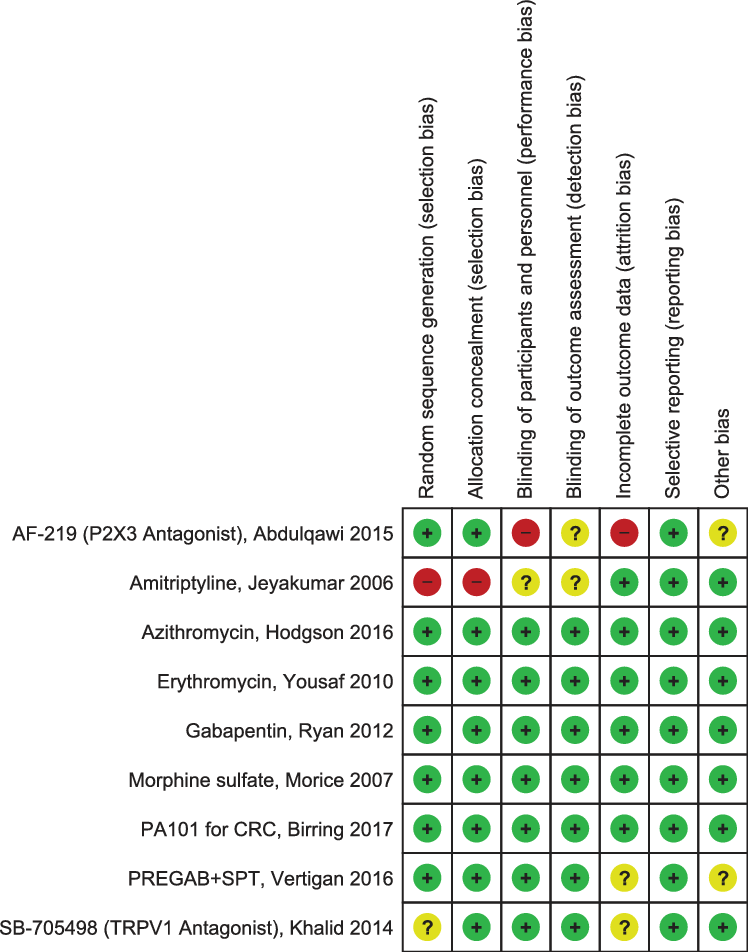 | 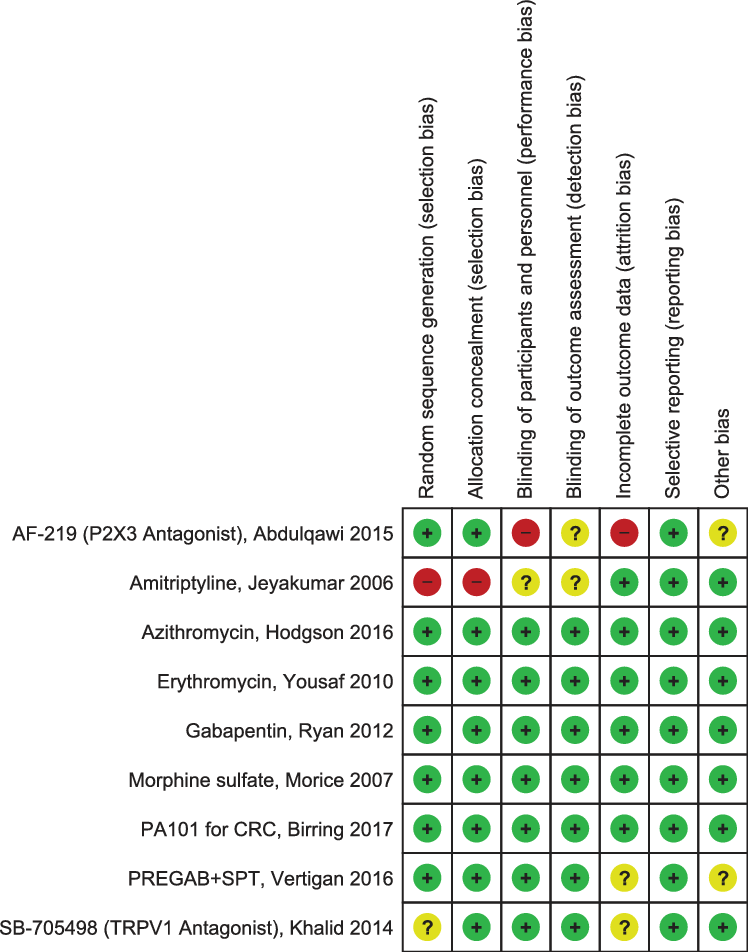 | 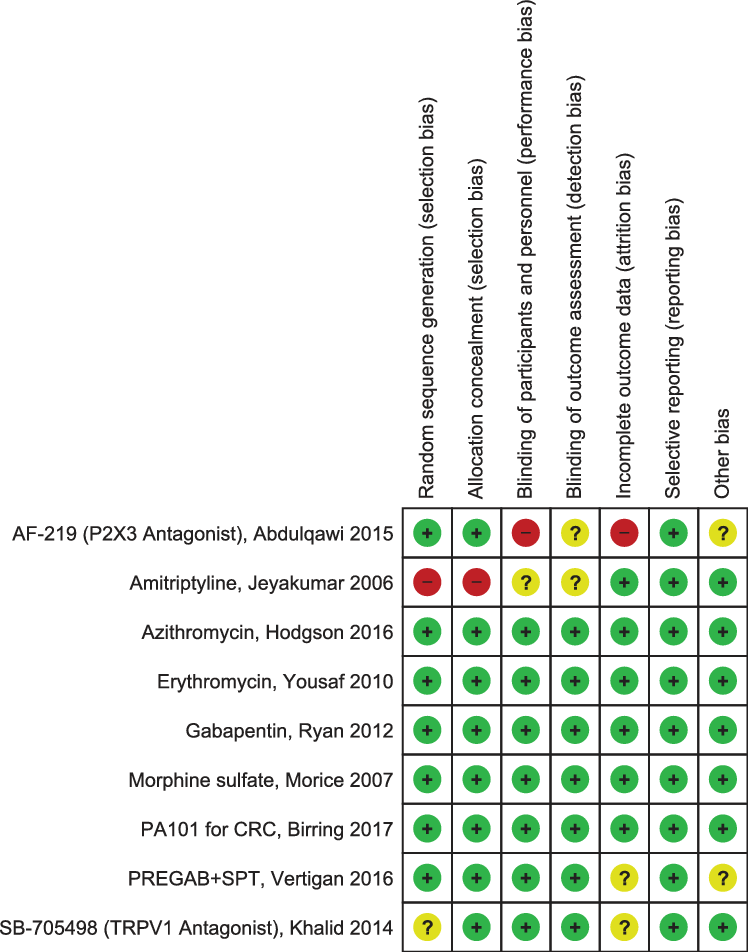 | 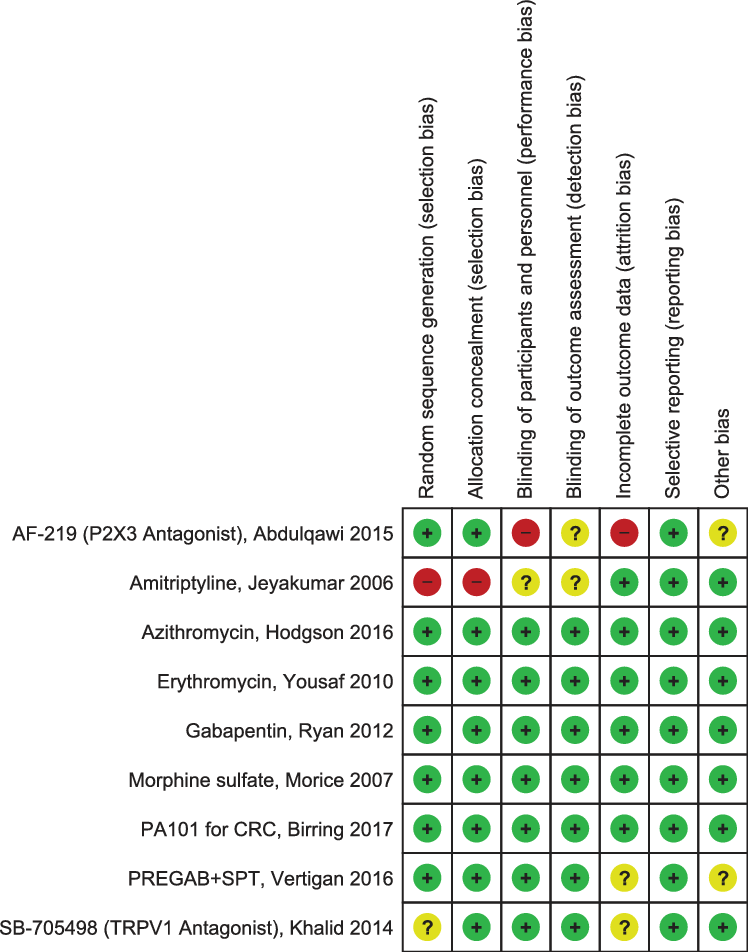 | 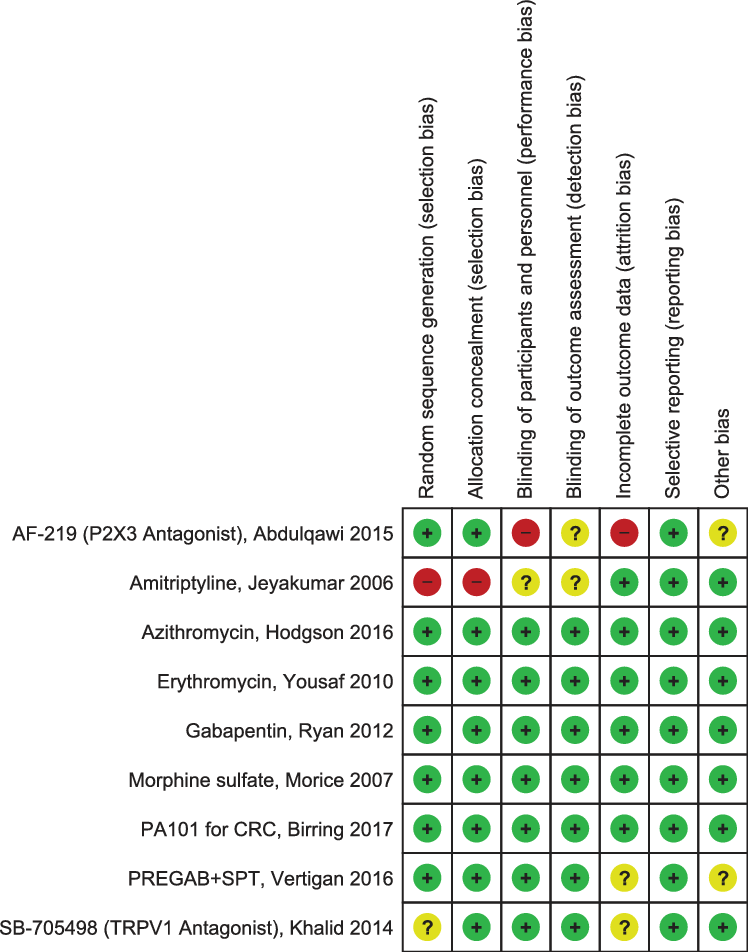 | 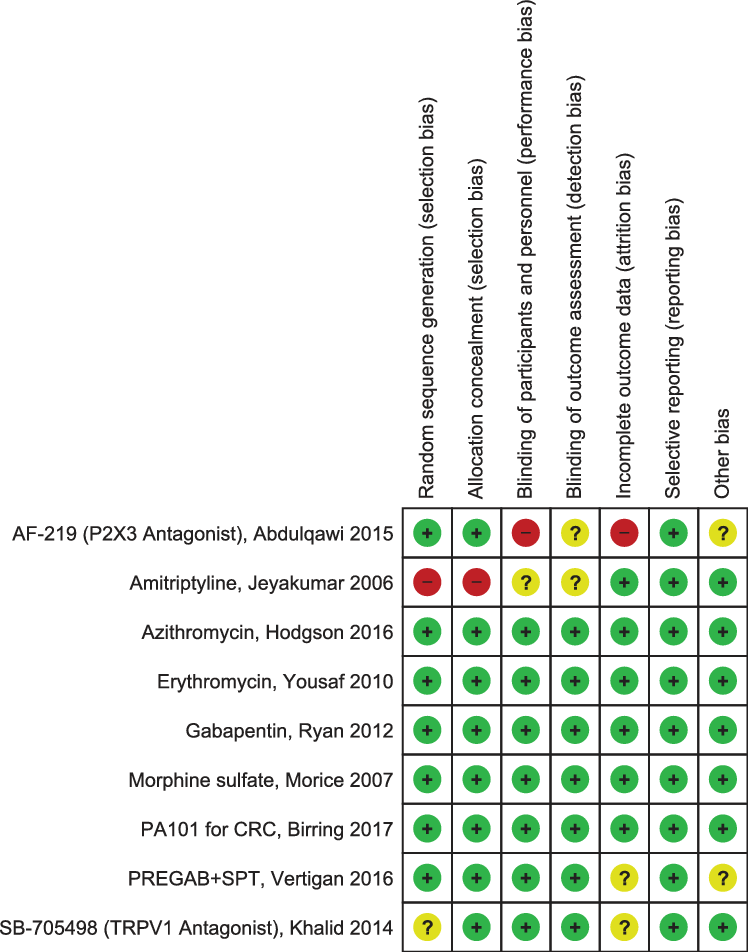 | 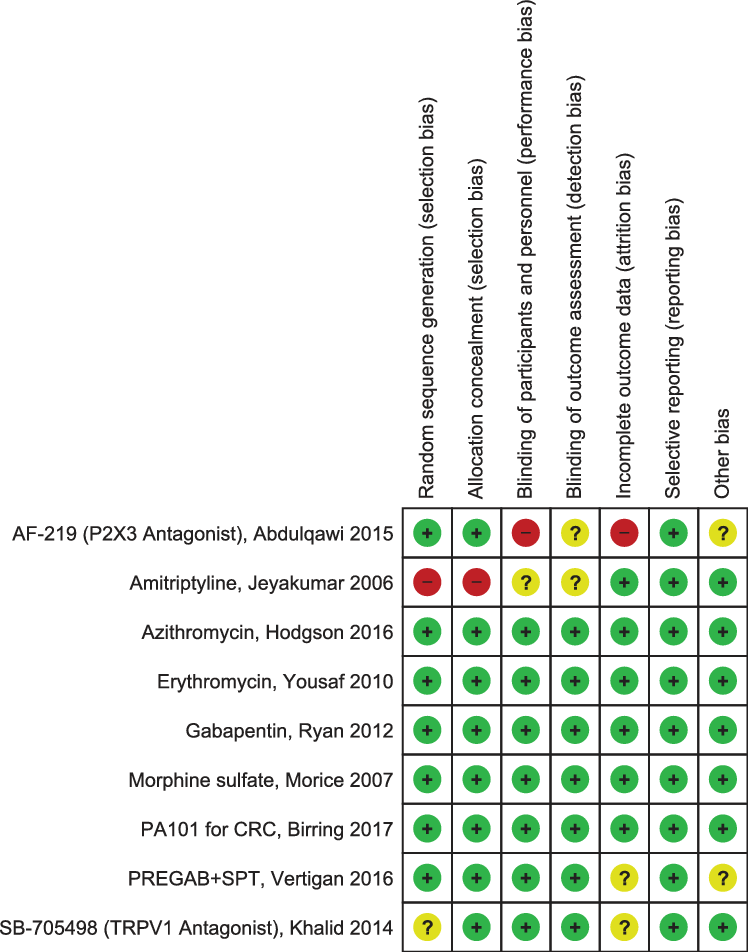 | 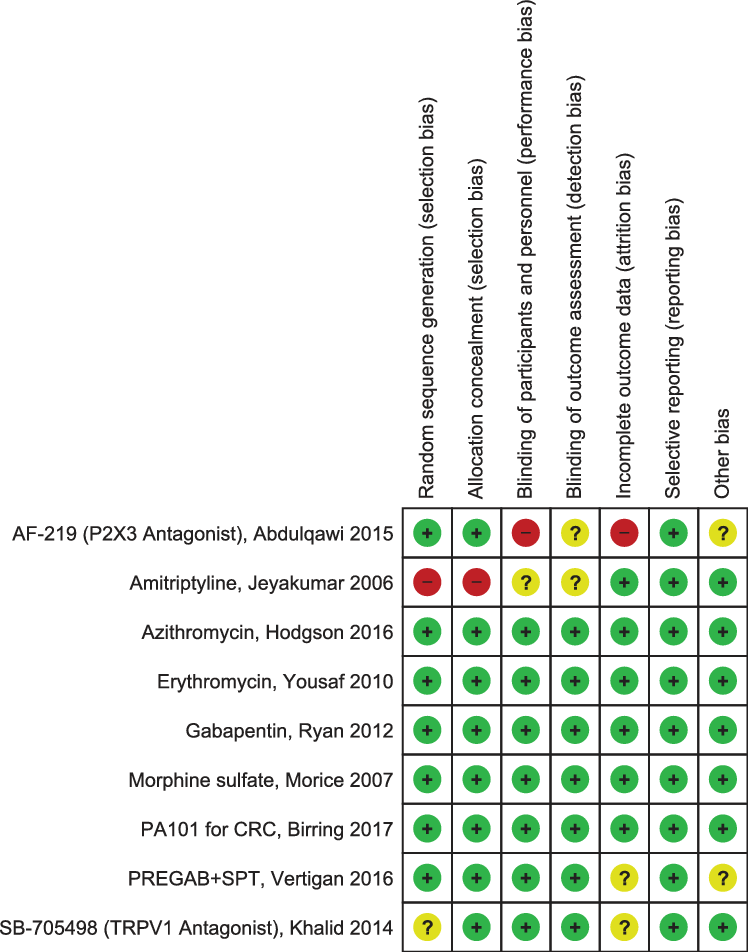 | 4 |
| Hummel et al., 2017; 2018 | RCT | 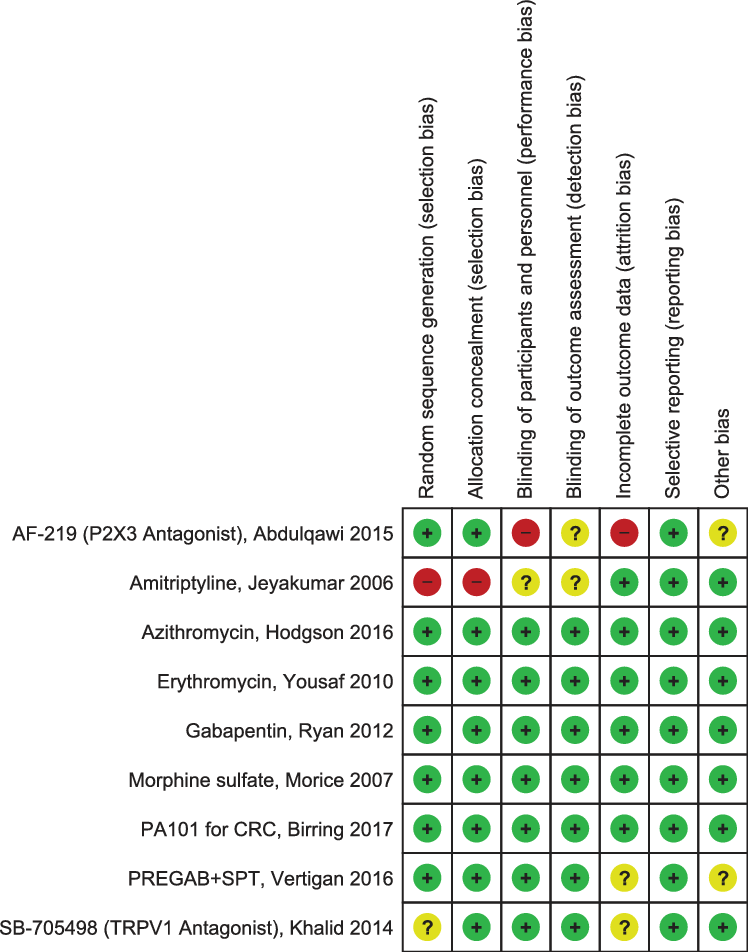 | 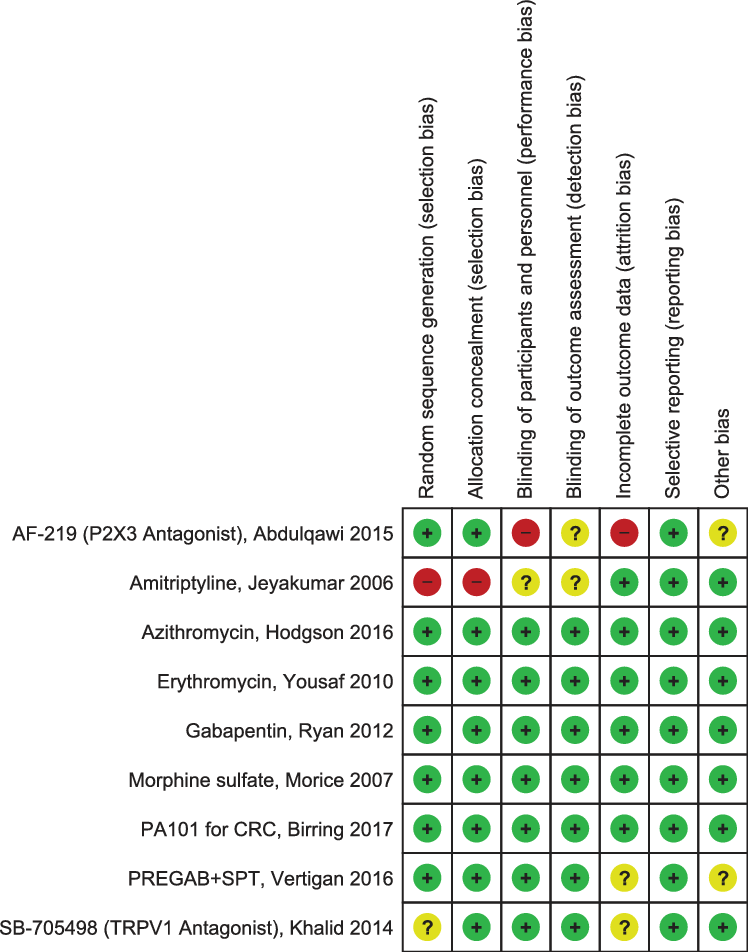 | 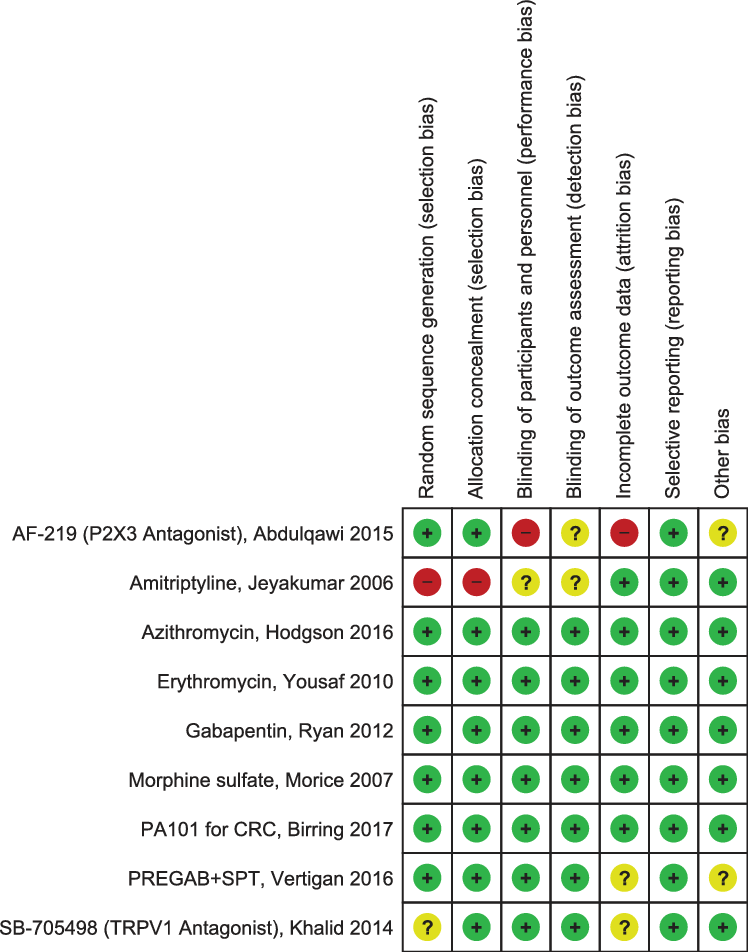 | 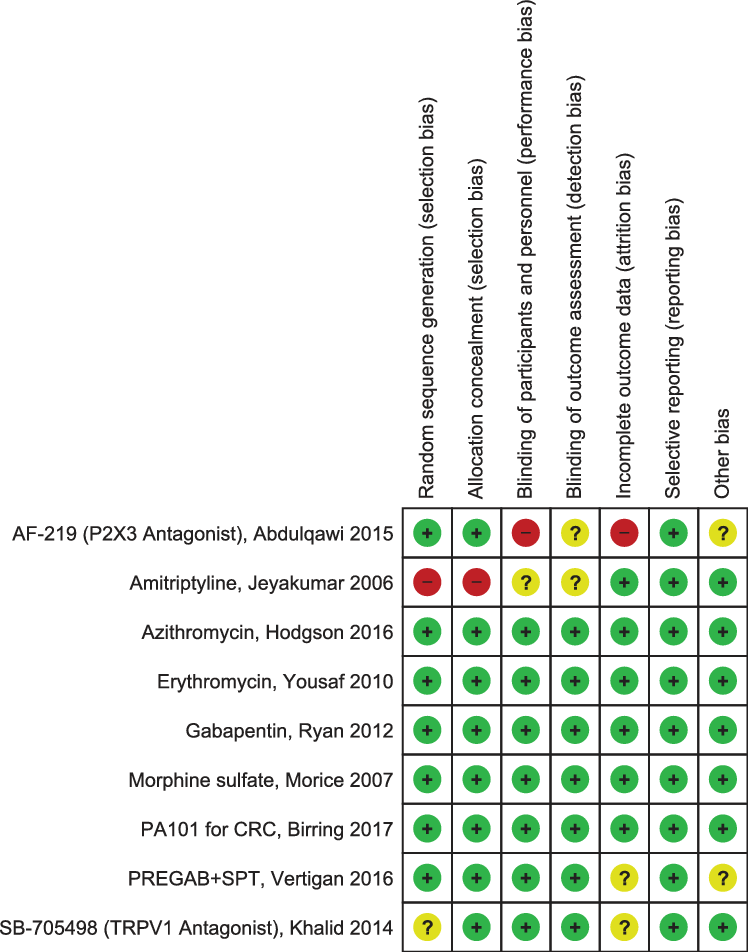 | 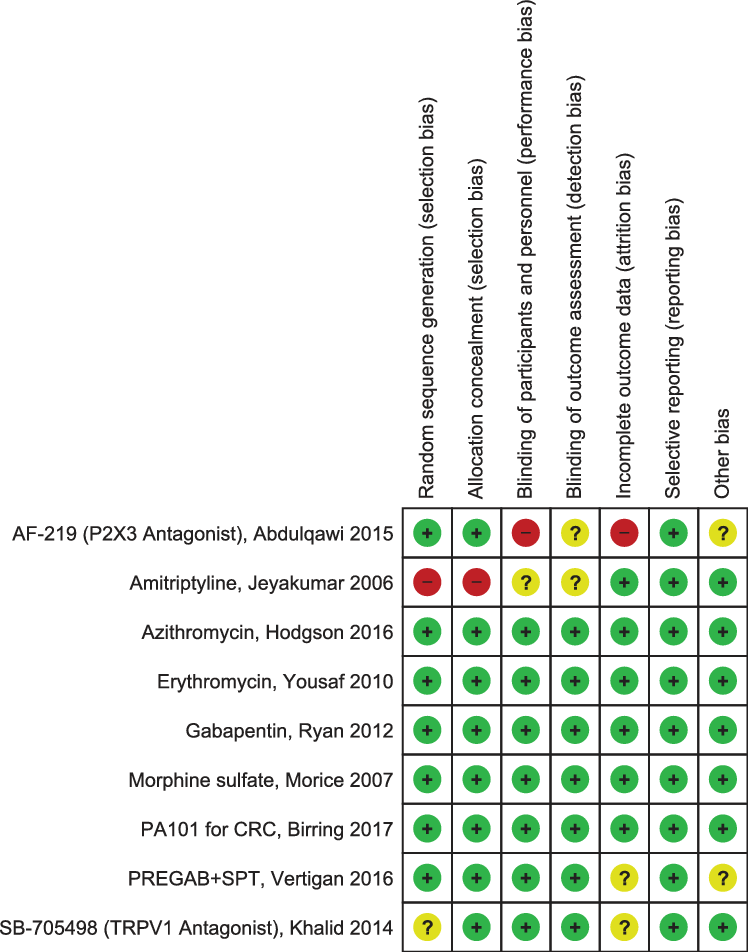 | 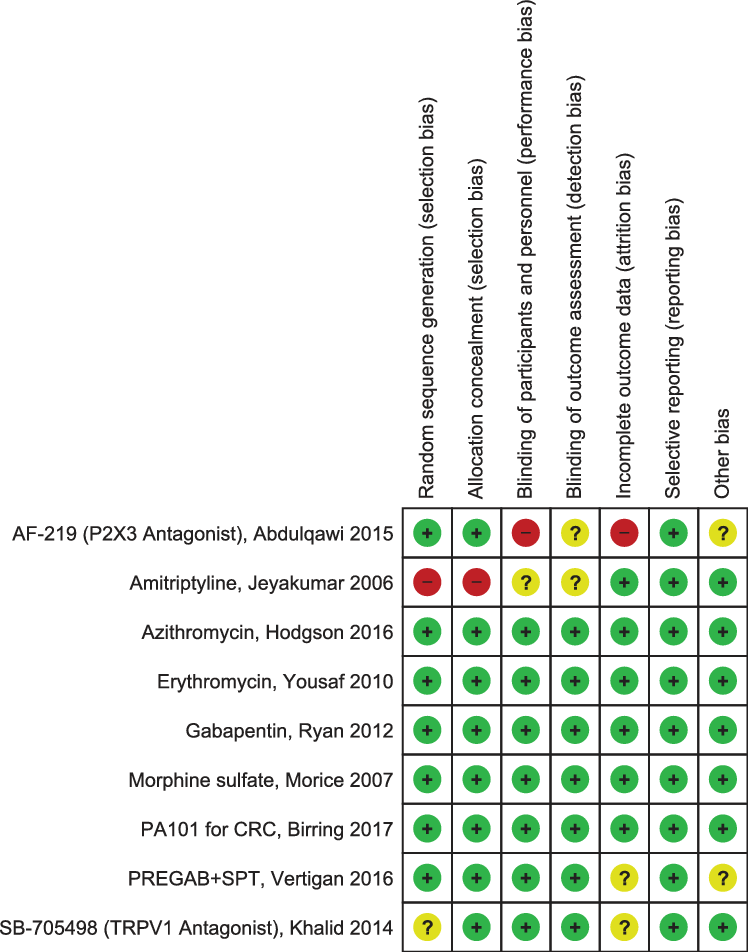 | 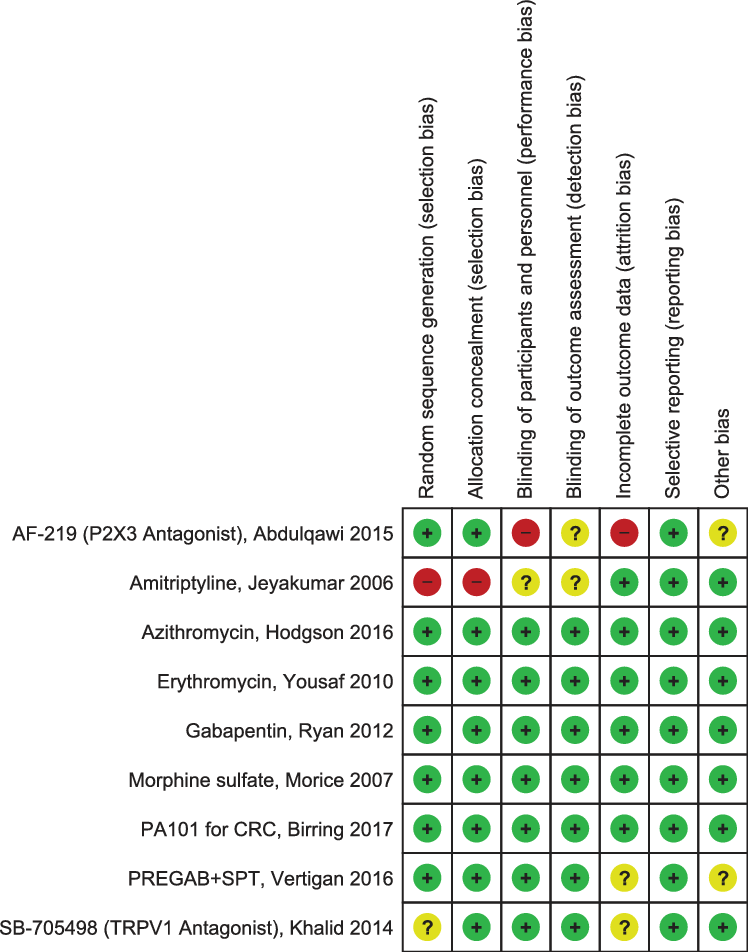 | 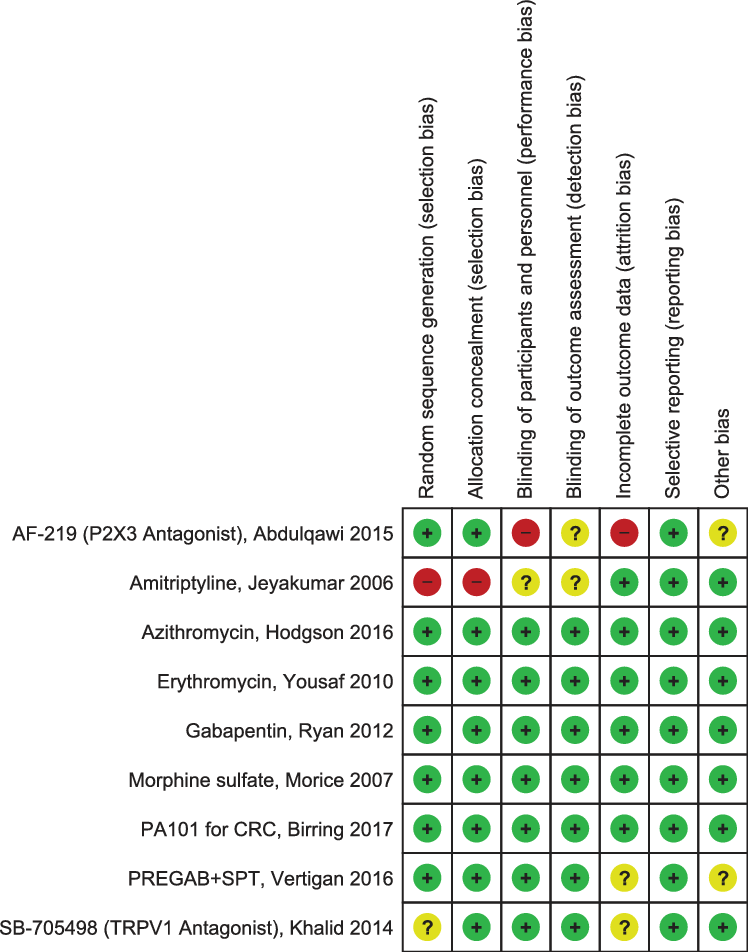 | 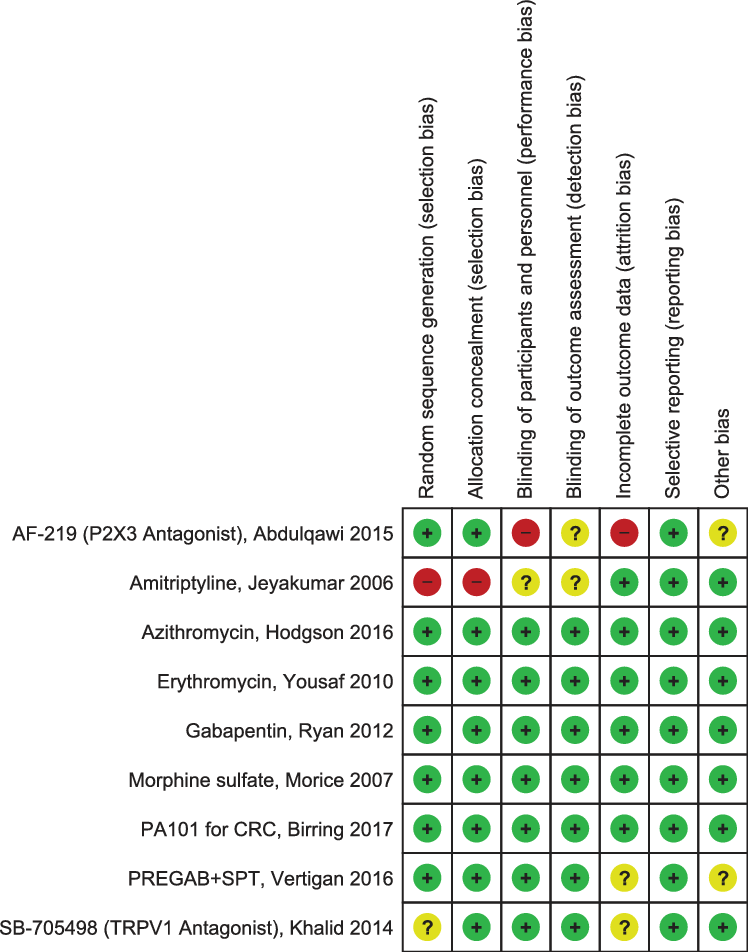 | 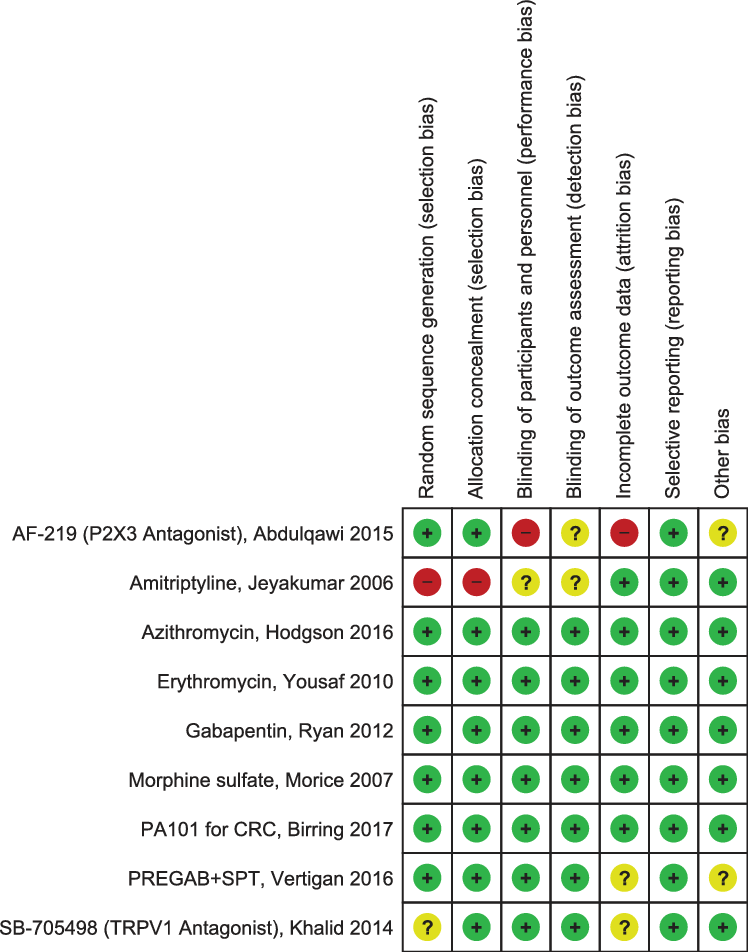 | 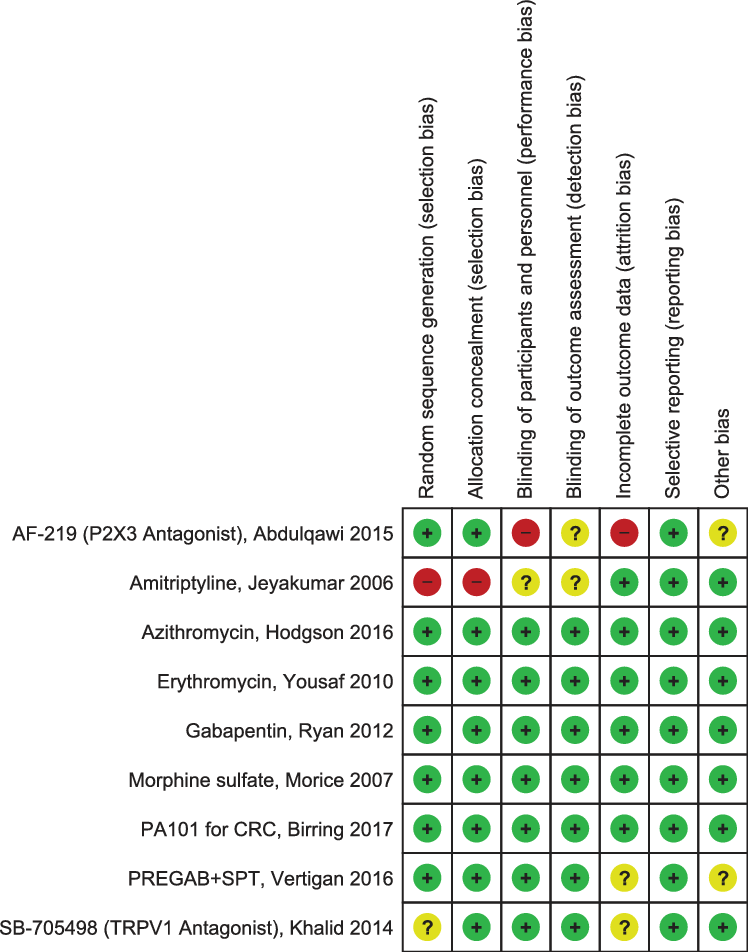 | 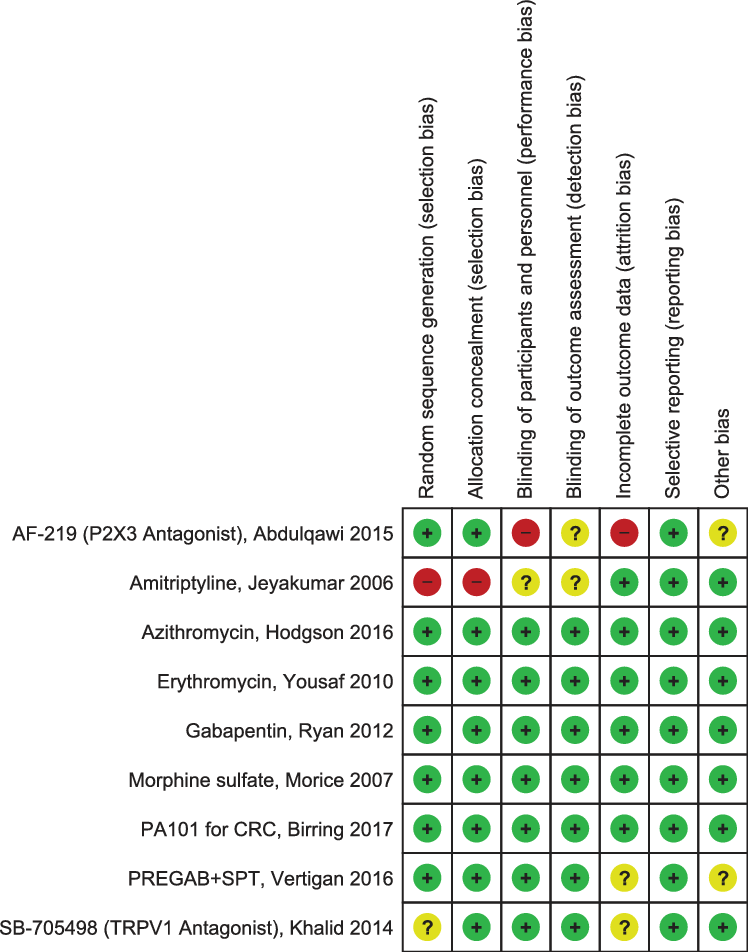 | 11 |
| Duijts et al., 2012 | RCT | 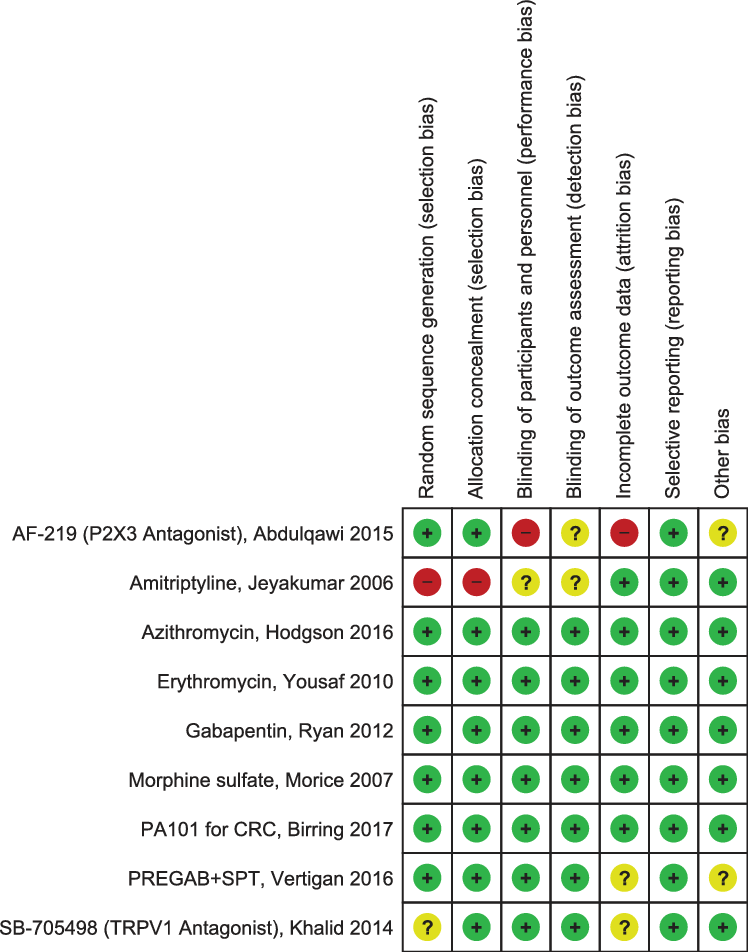 | 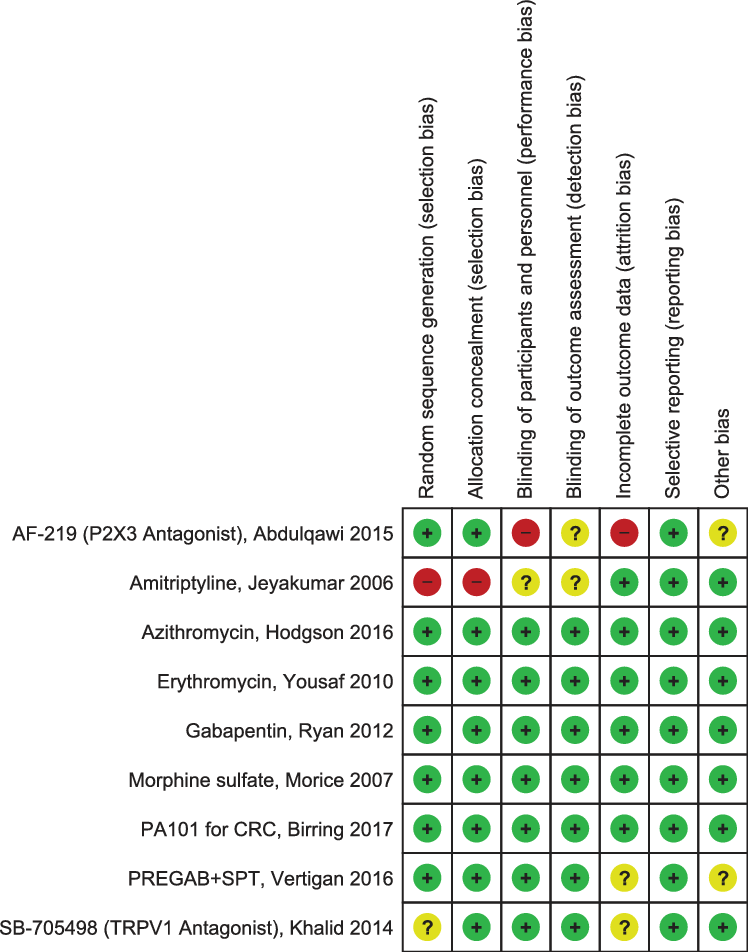 | 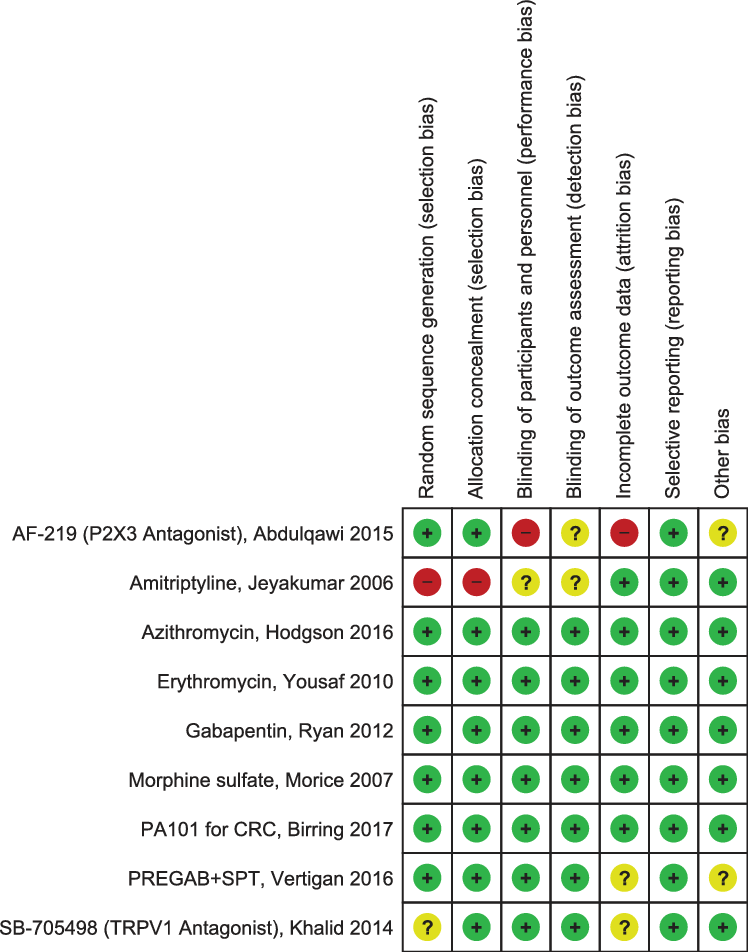 | 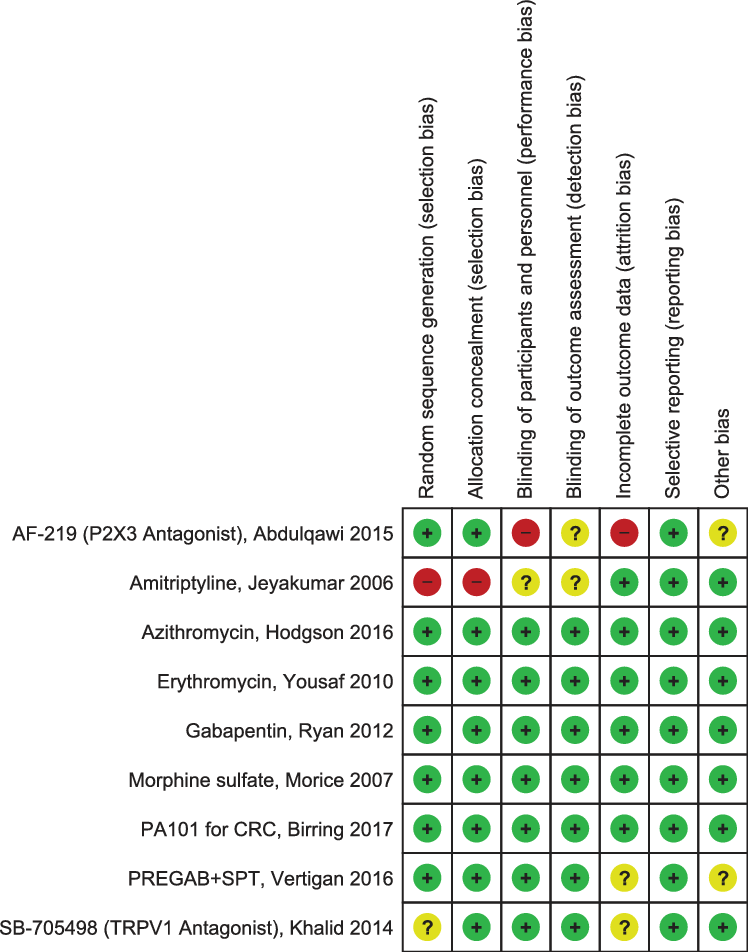 | 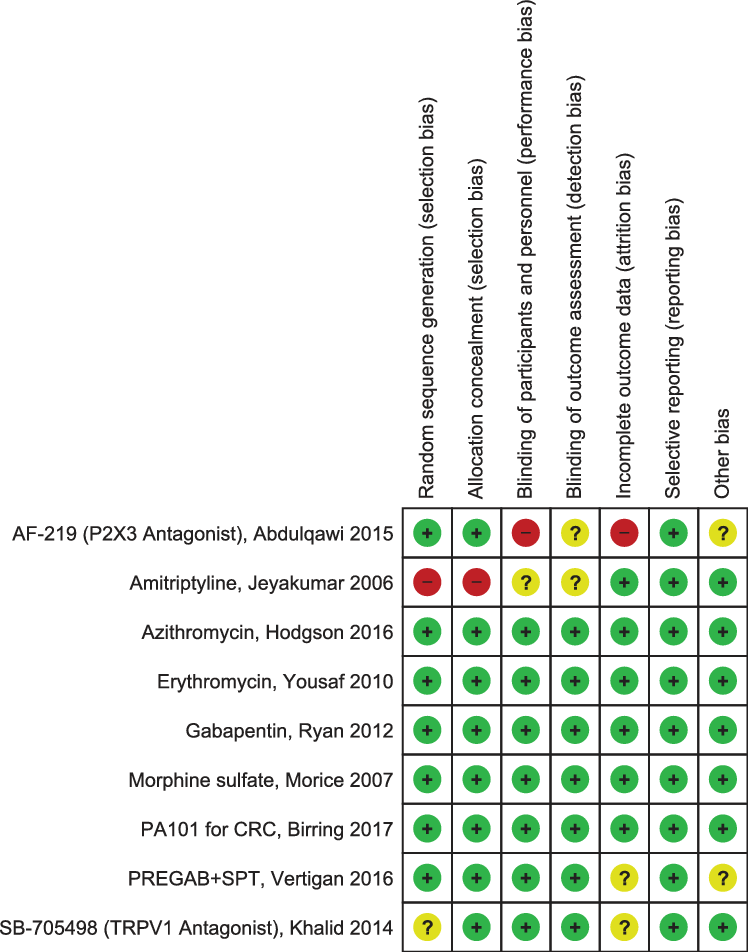 | 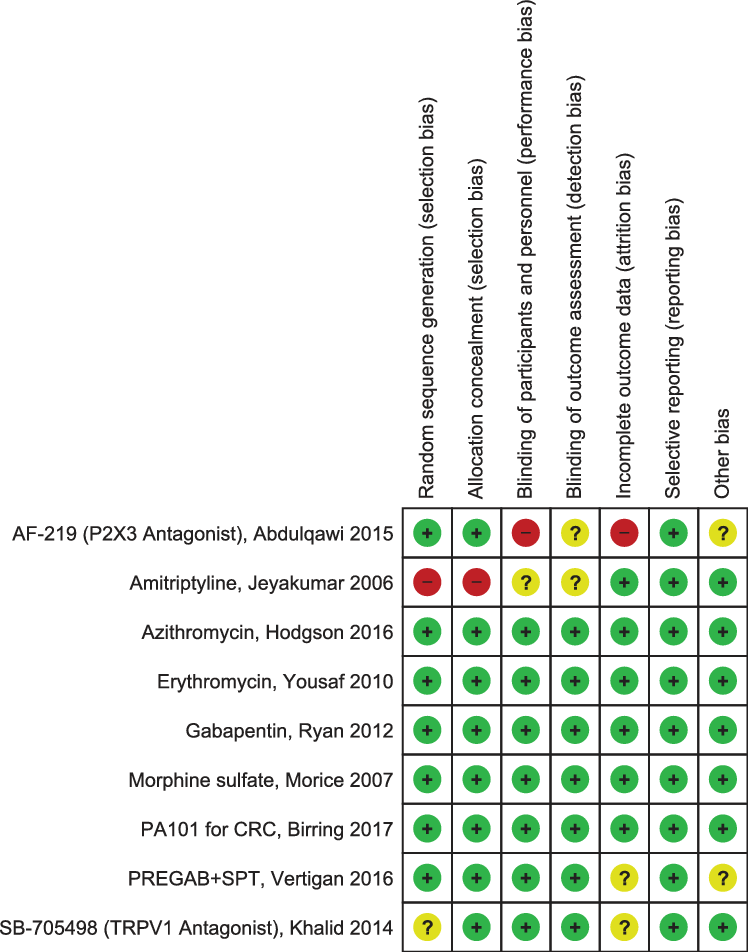 | 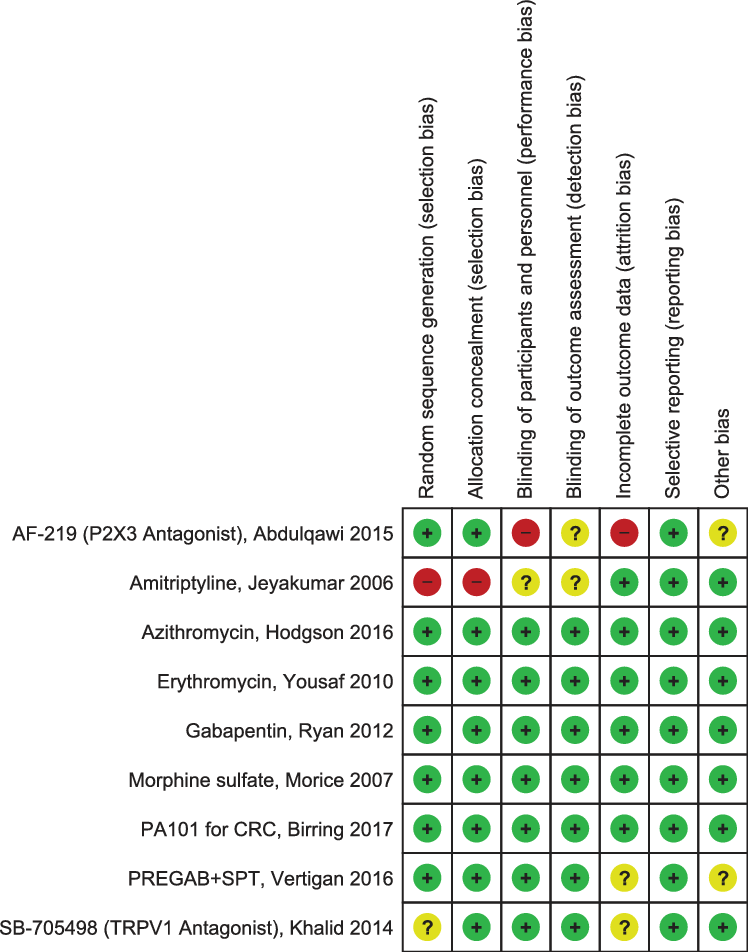 | 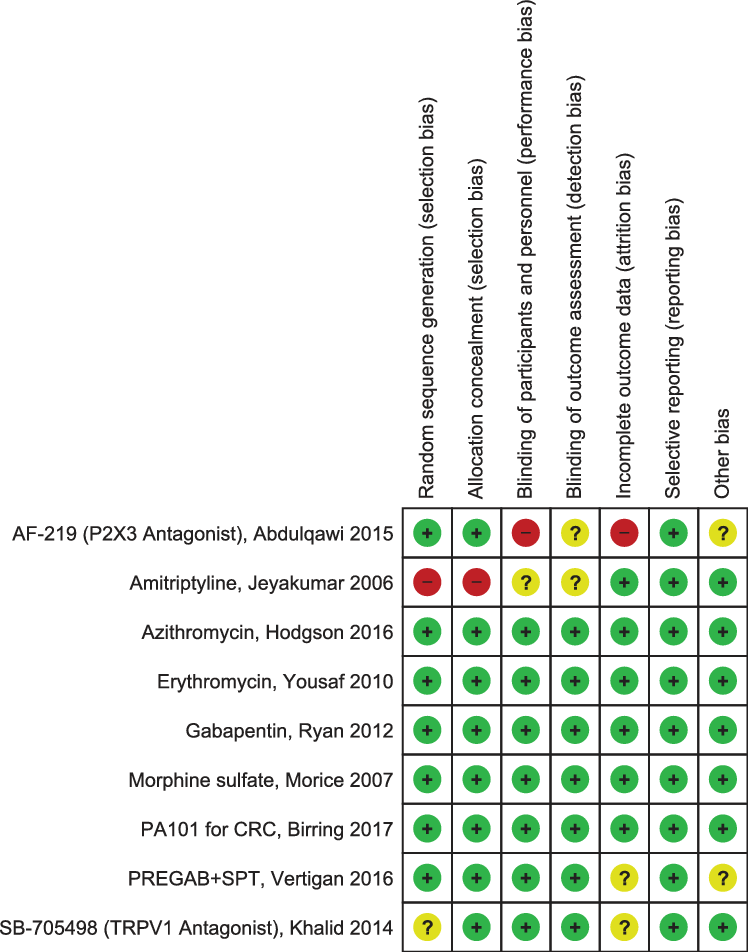 | 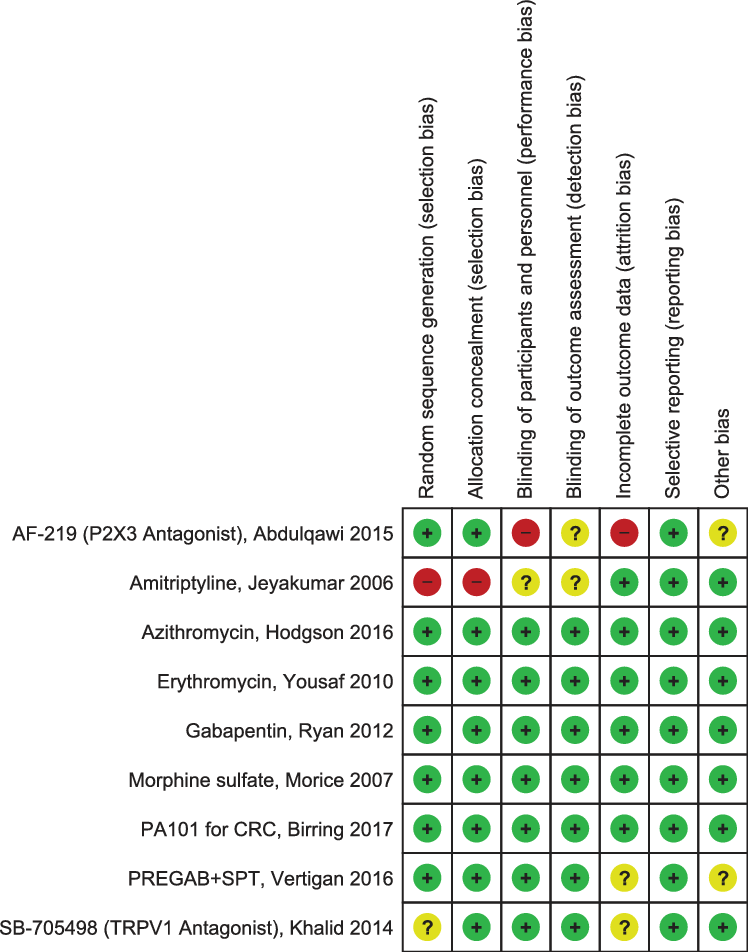 | 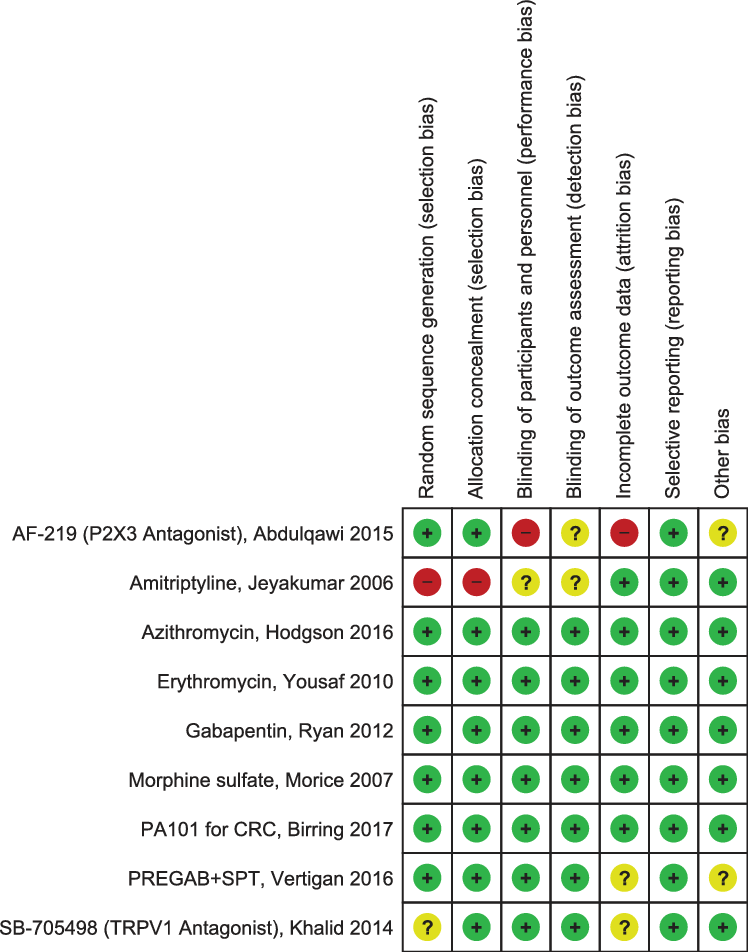 | 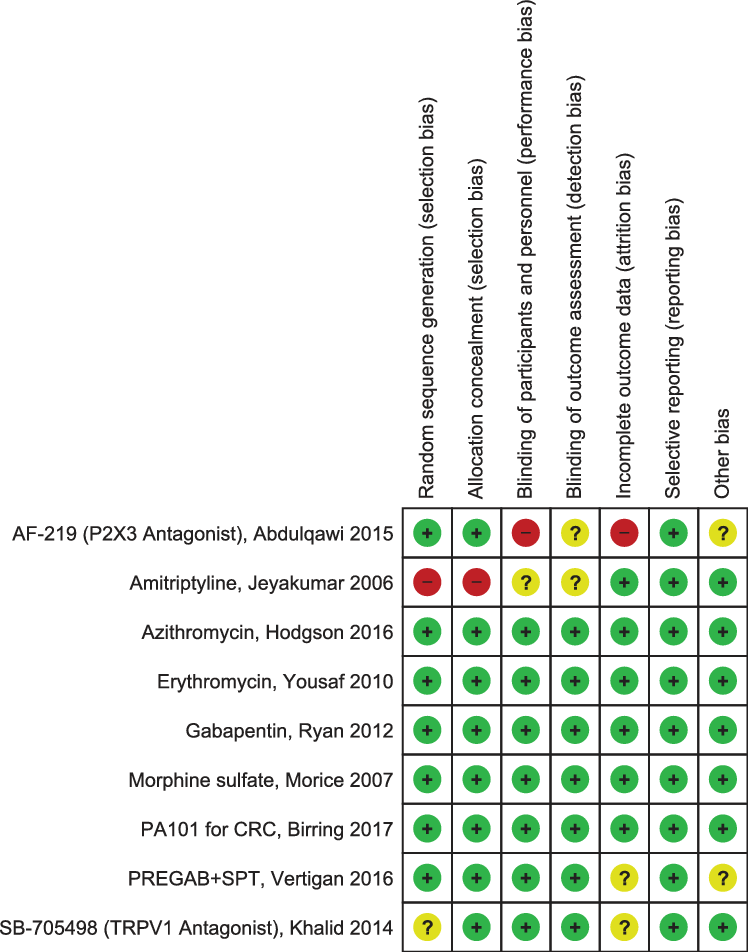 | 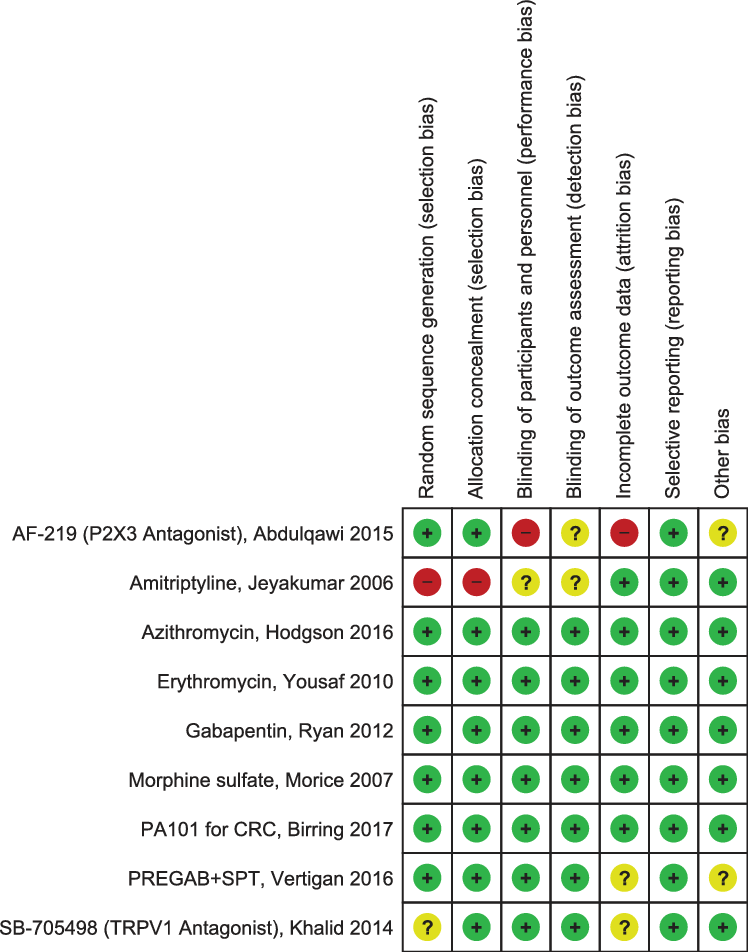 | 11 |
| Schover et al., 2011 | RCT | 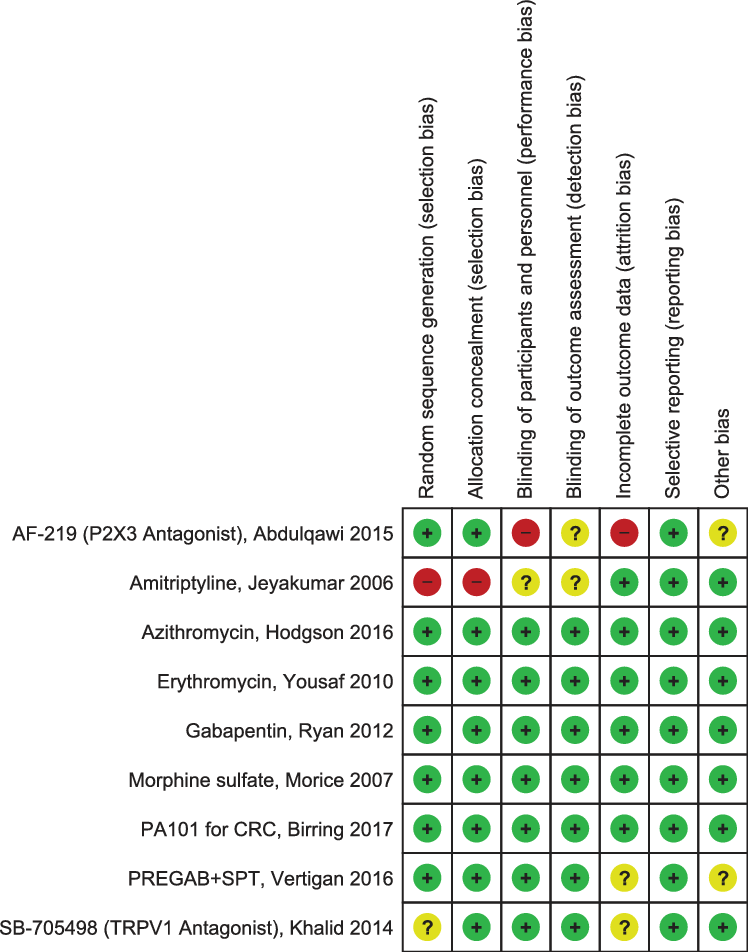 | 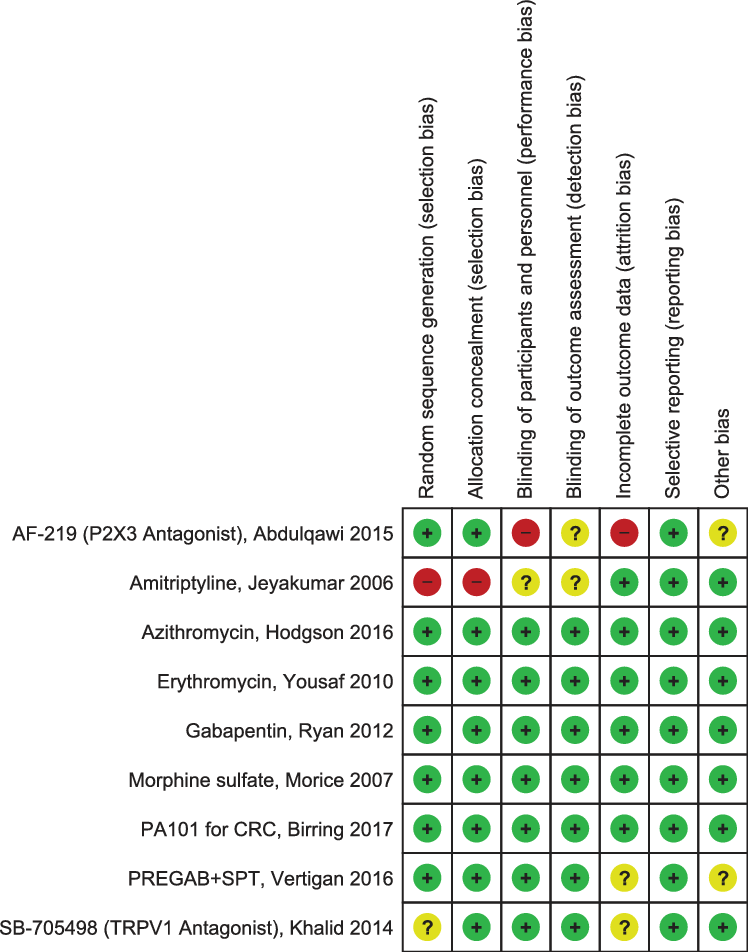 | 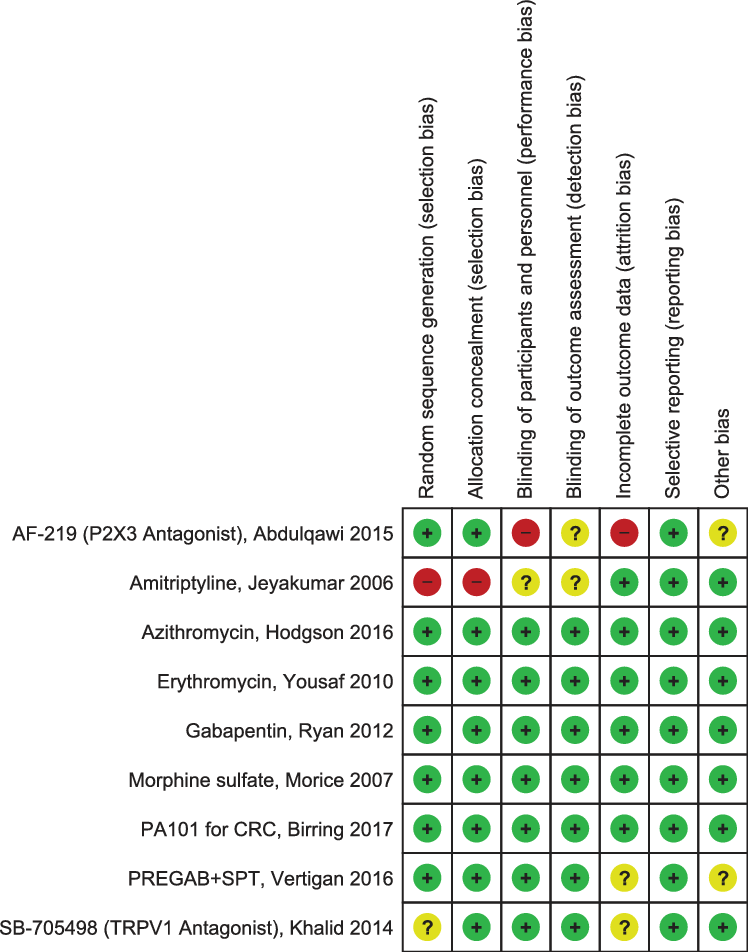 | 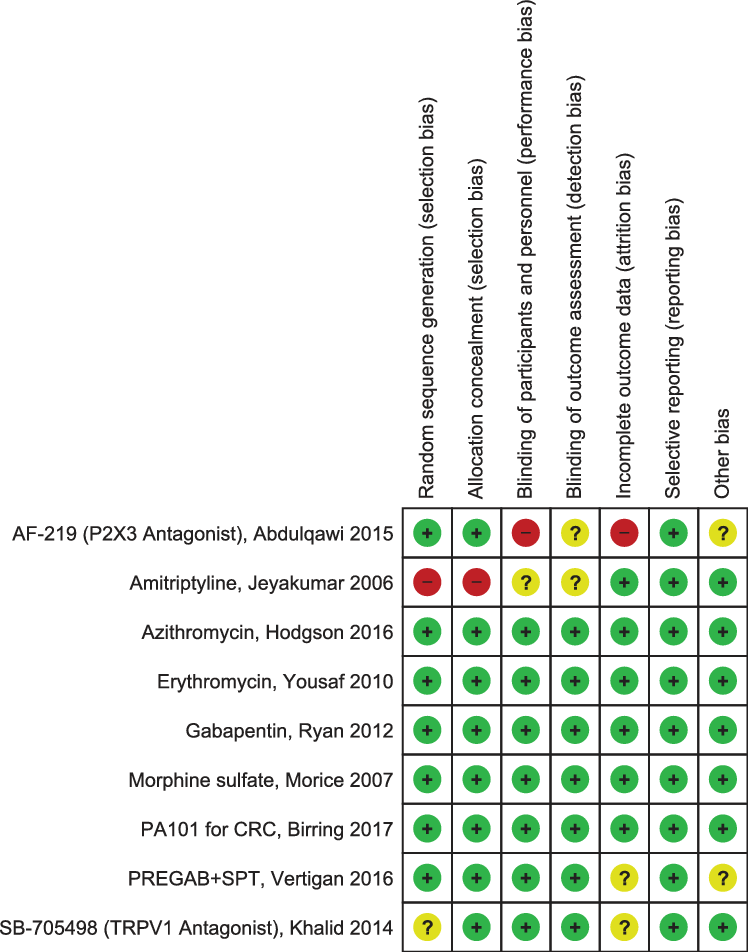 | 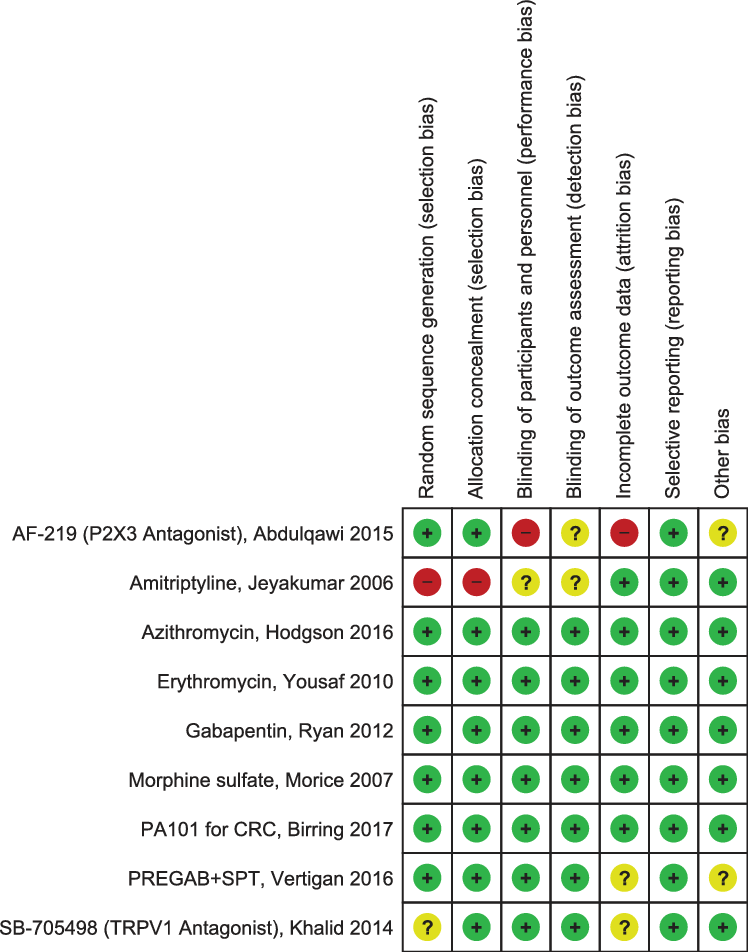 | 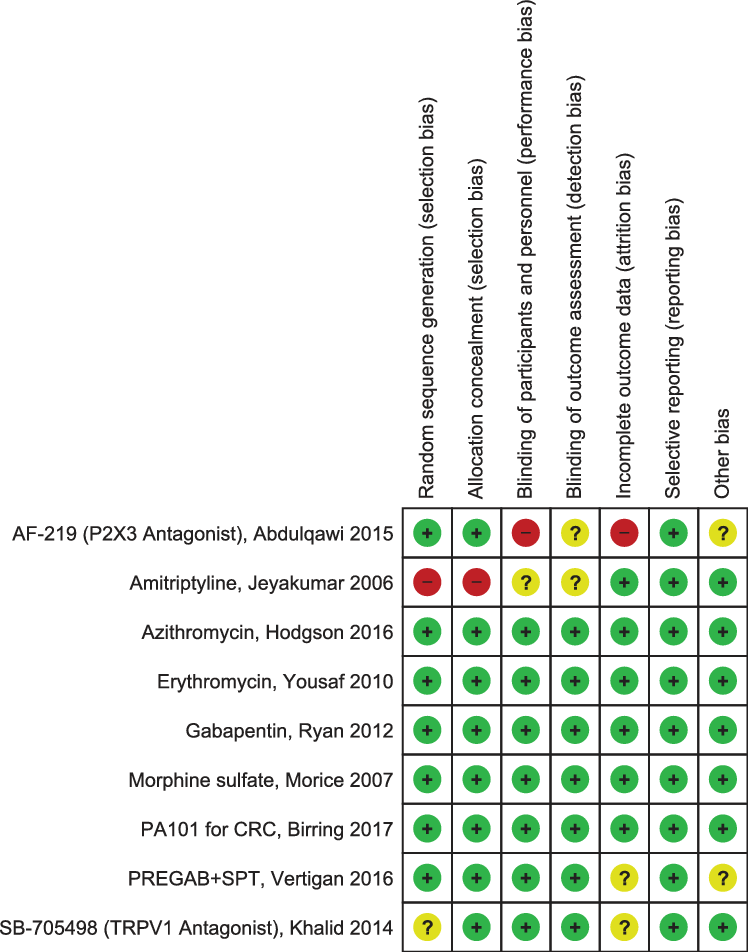 | 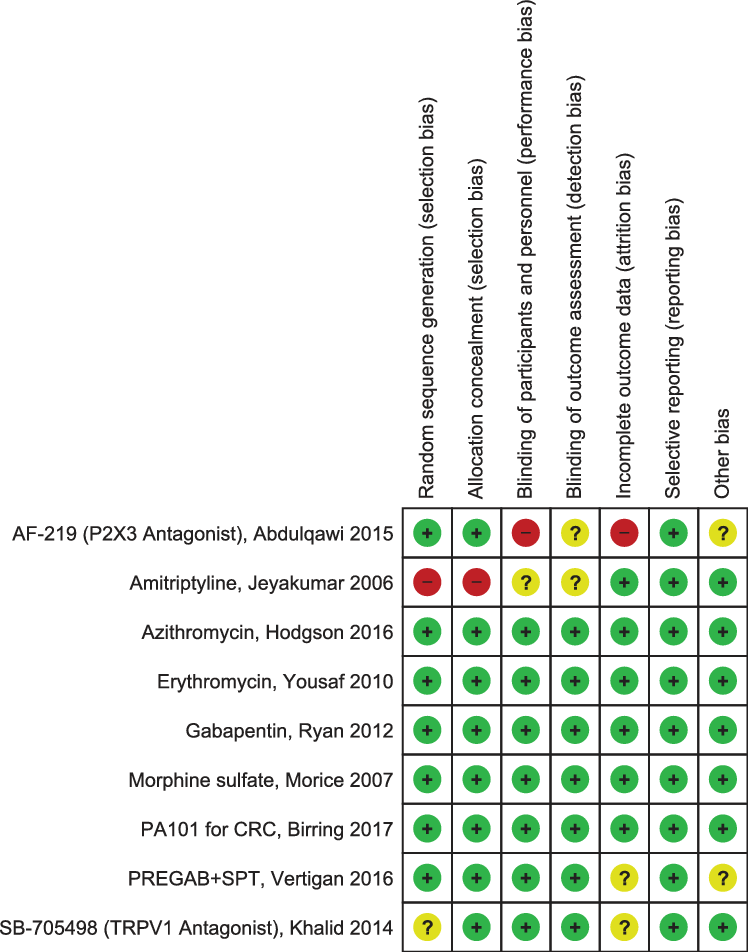 | 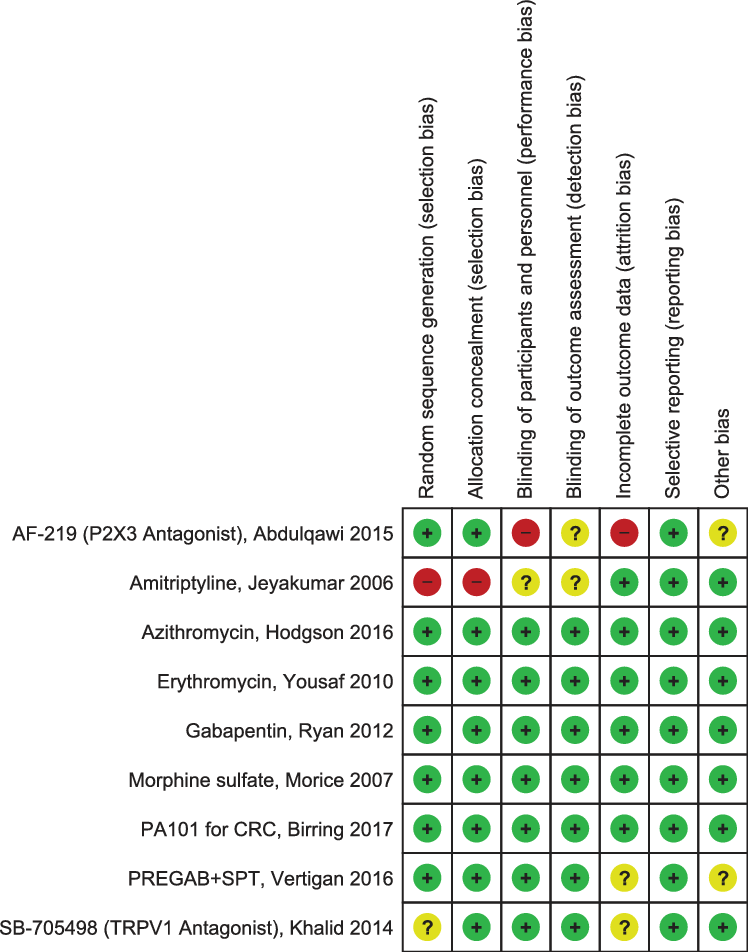 | 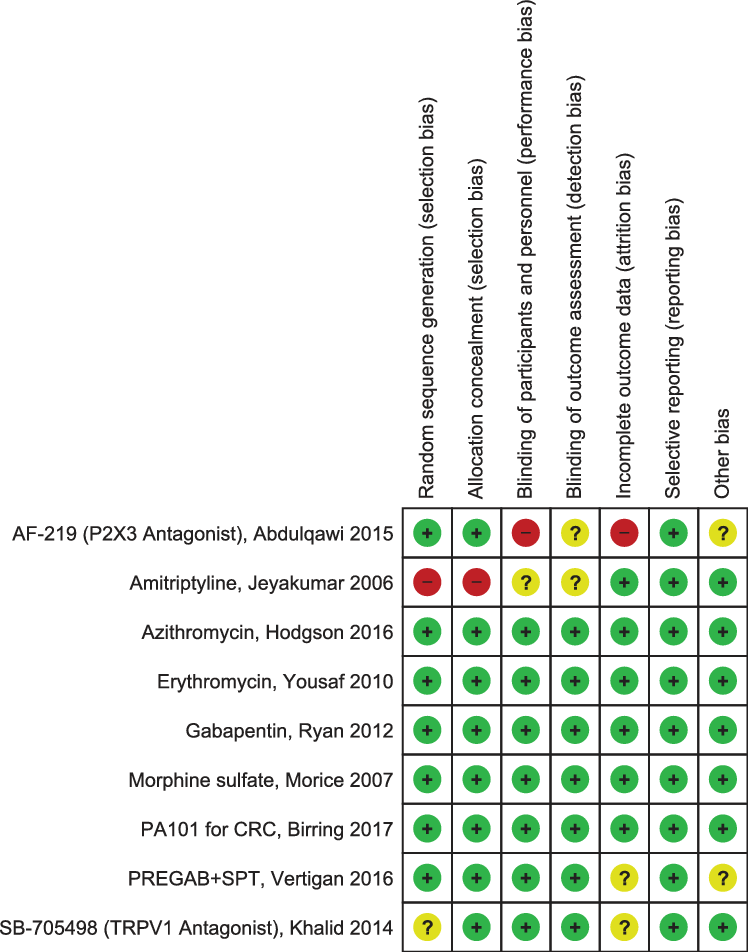 | 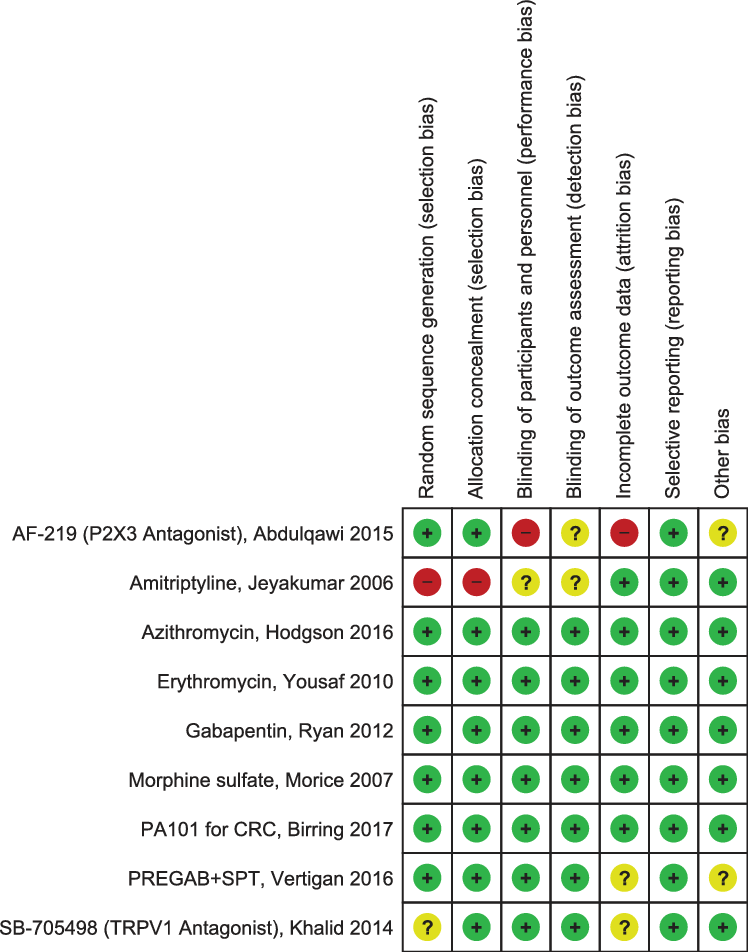 | 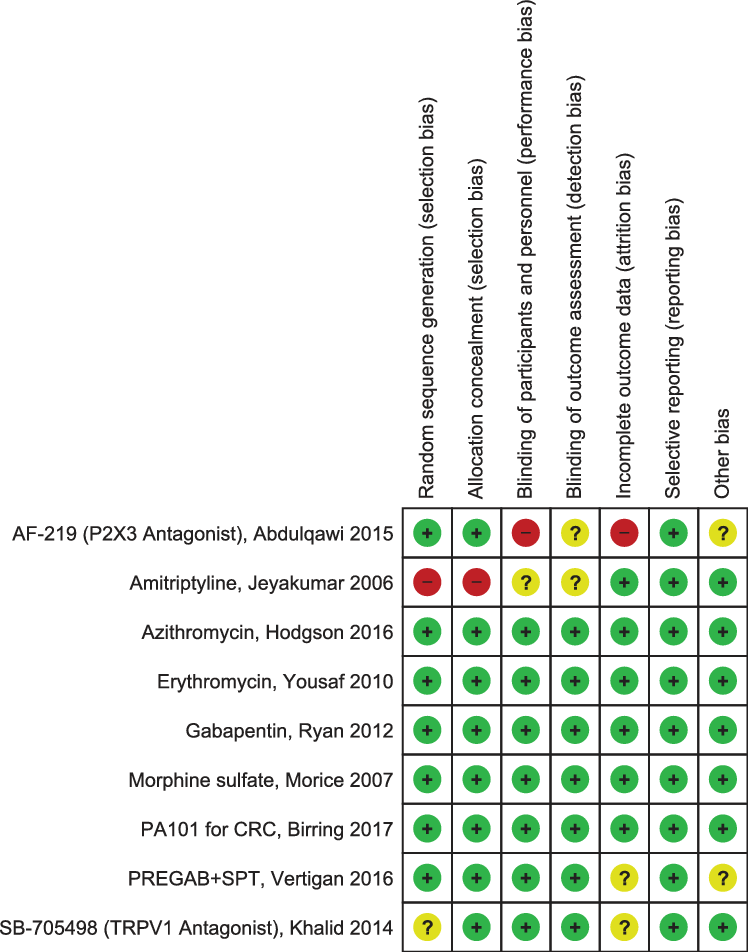 | 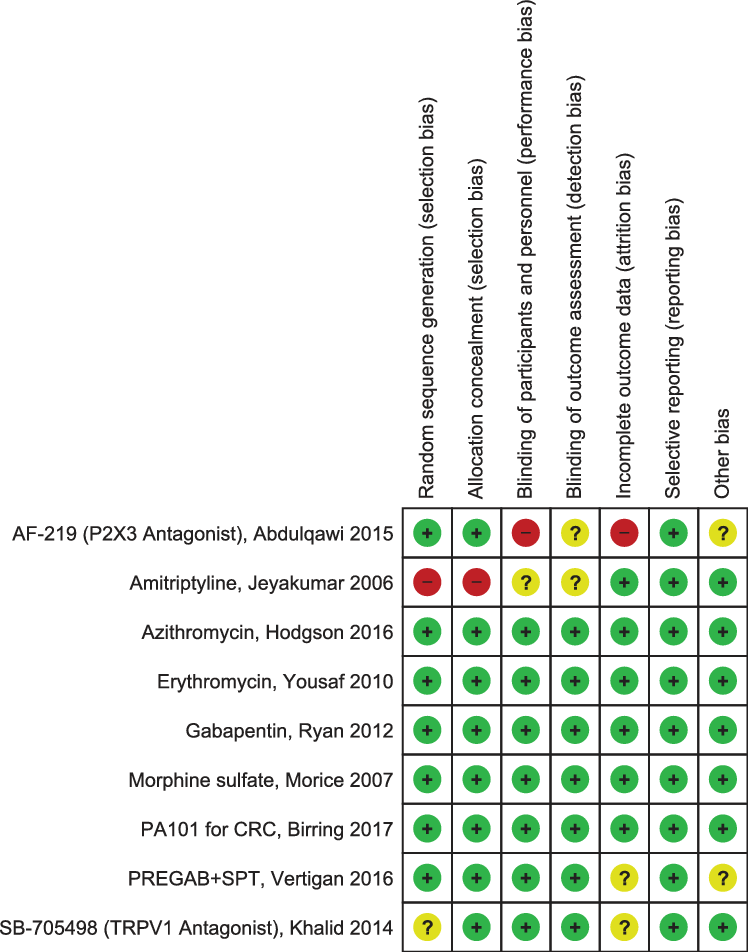 | 11 |
| Zangeneh et al., 2023 | Non-RCT | 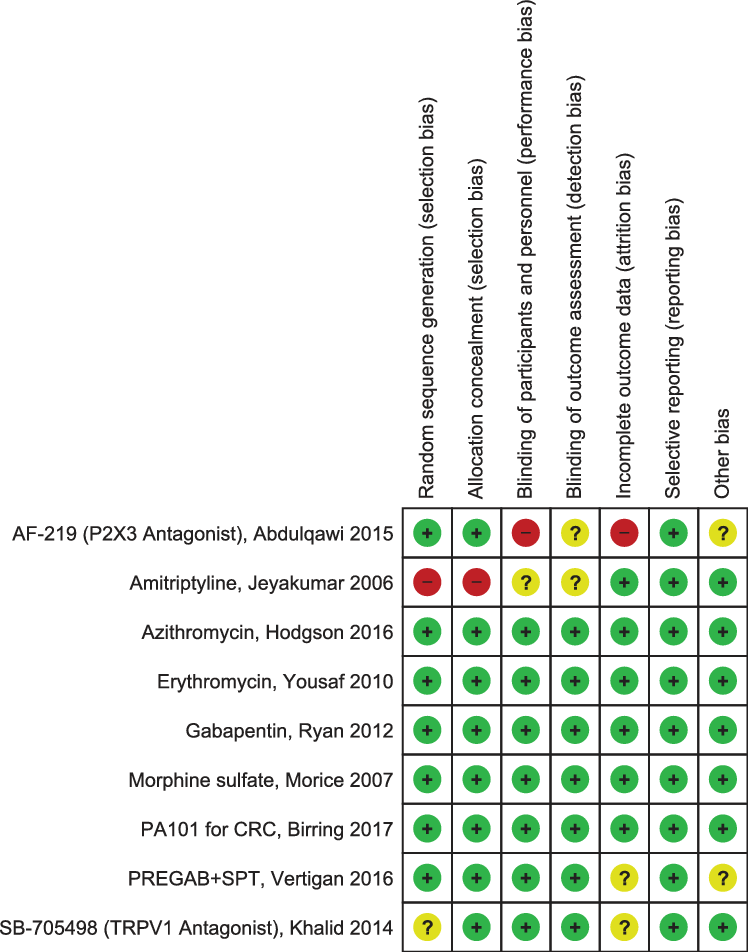 | 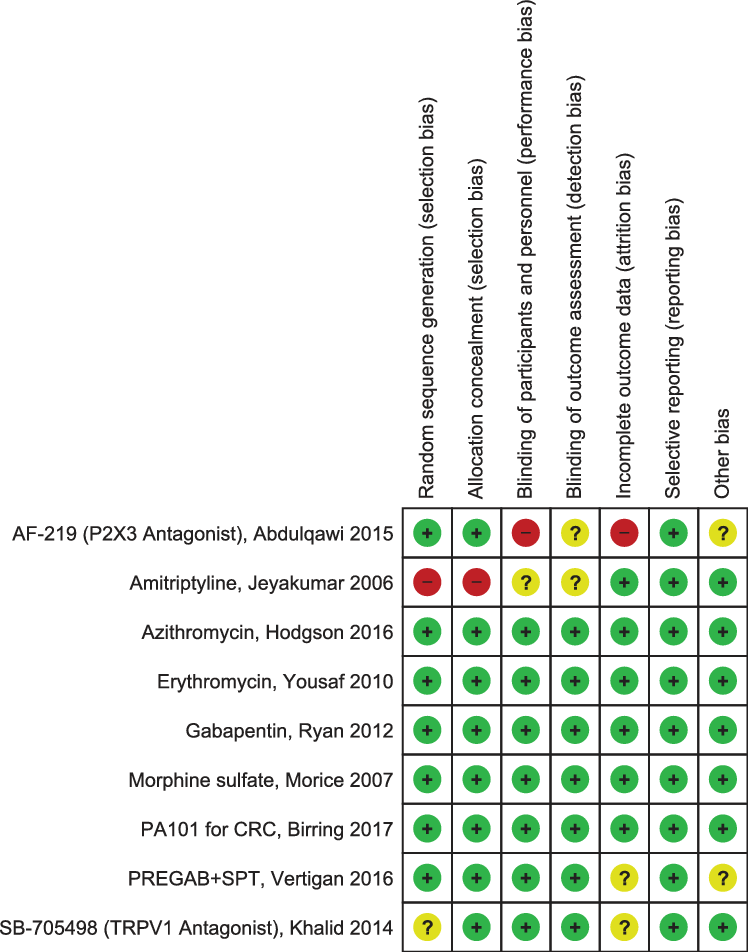 | 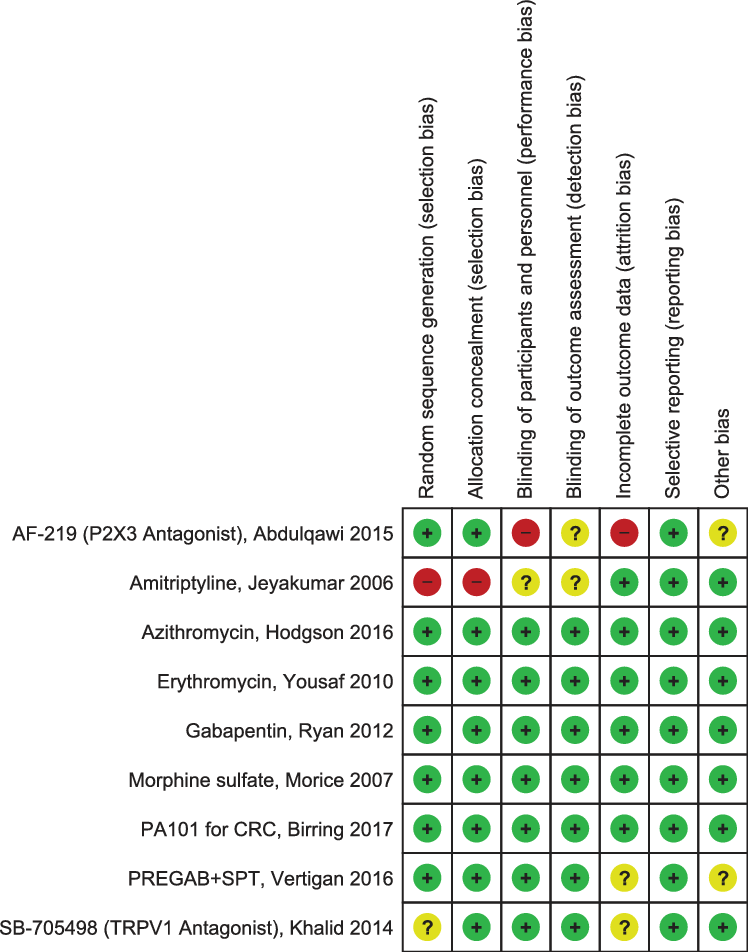 | 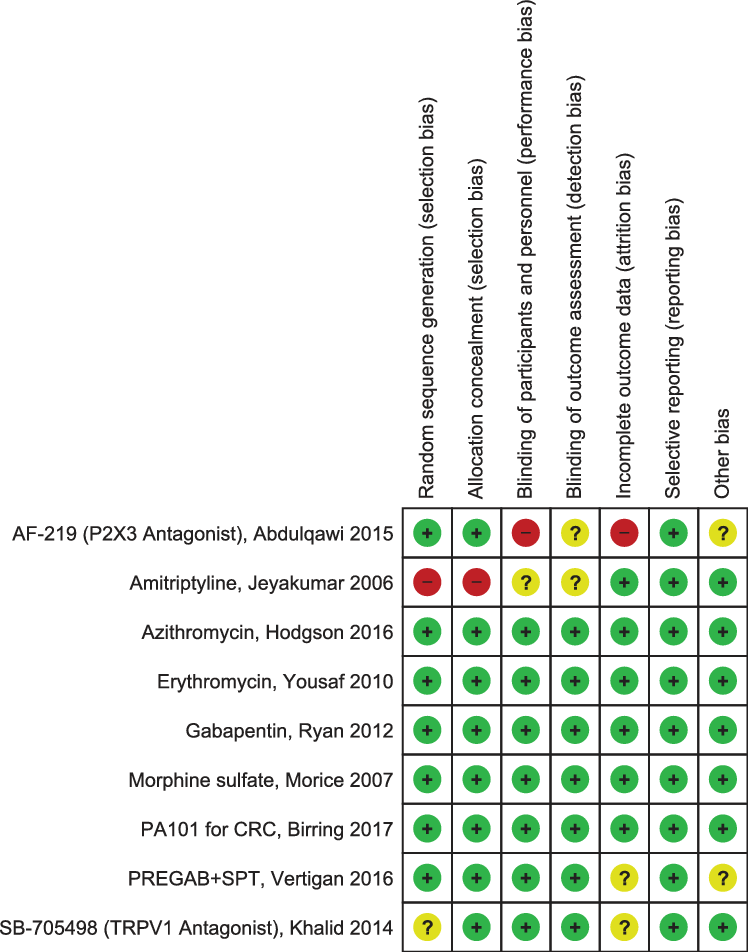 | 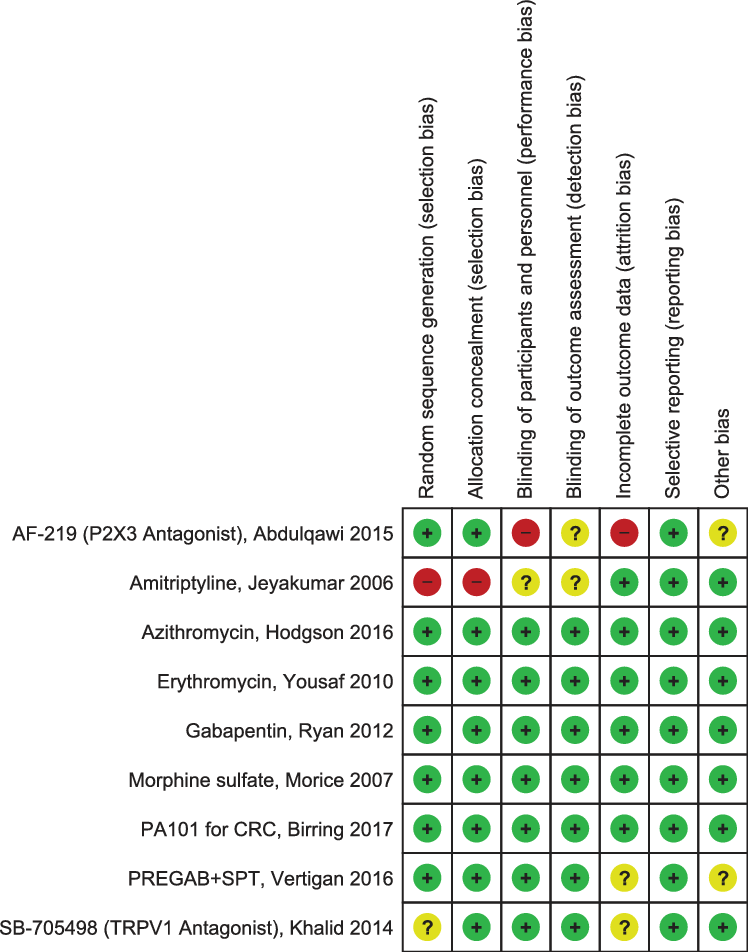 | 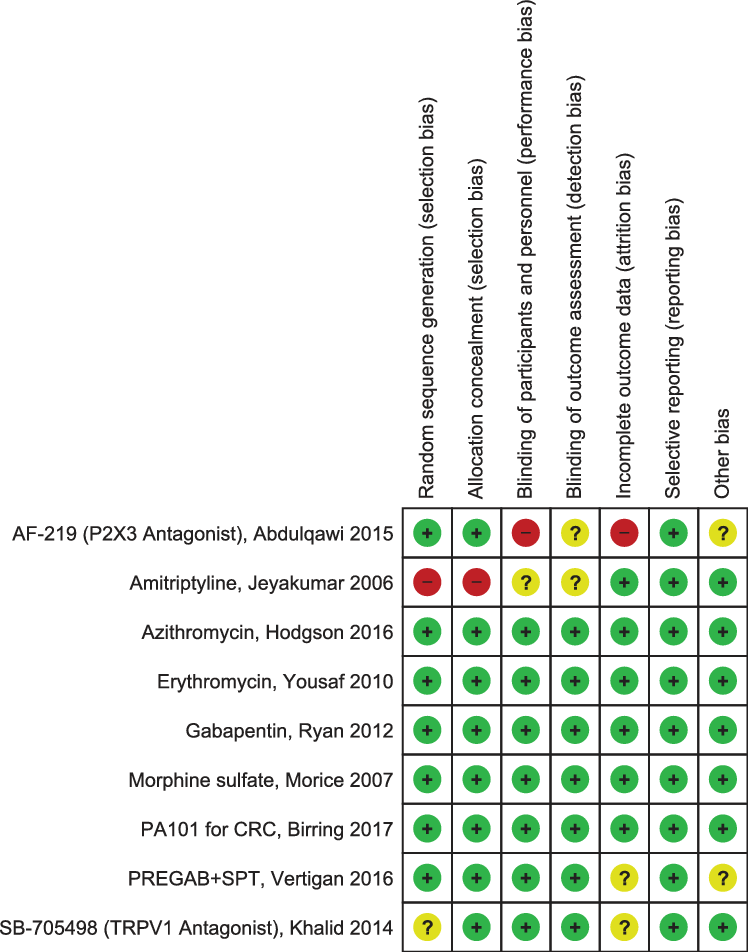 | 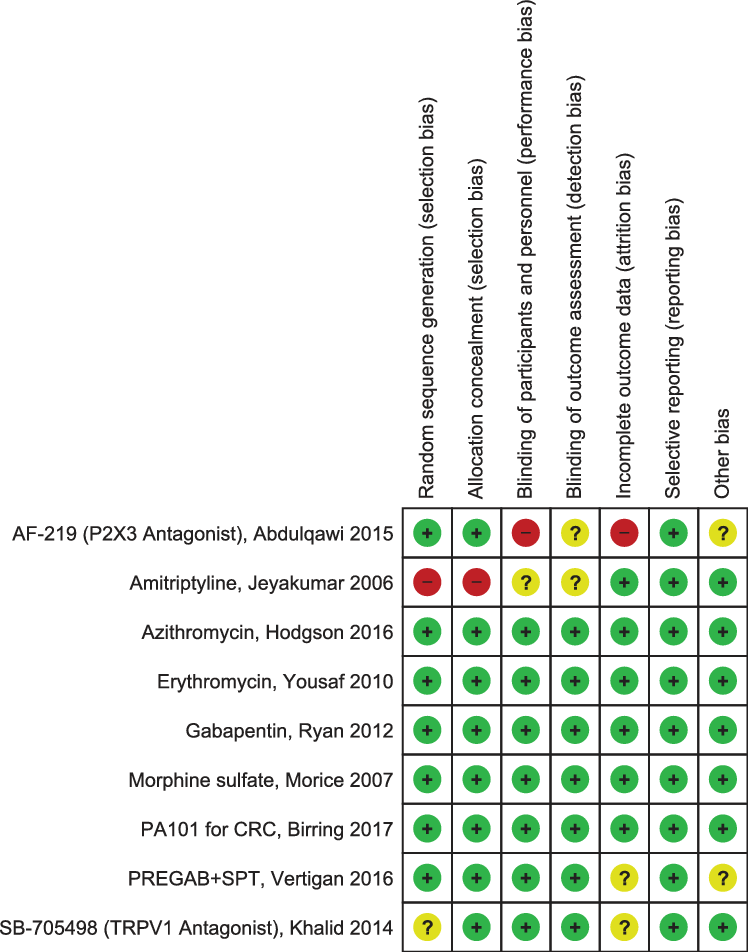 | 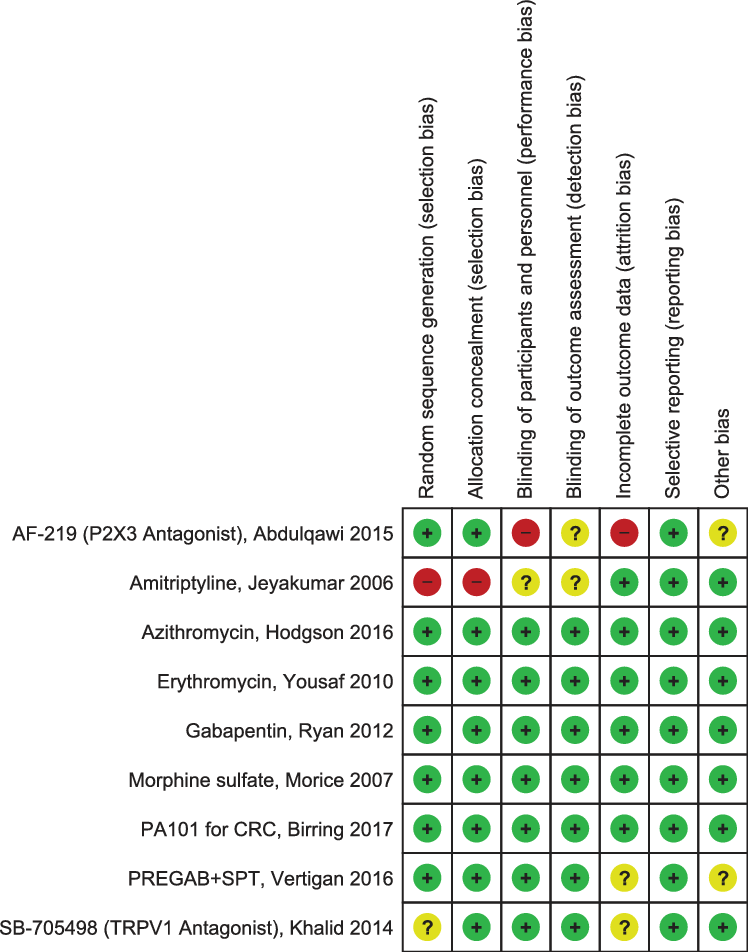 | 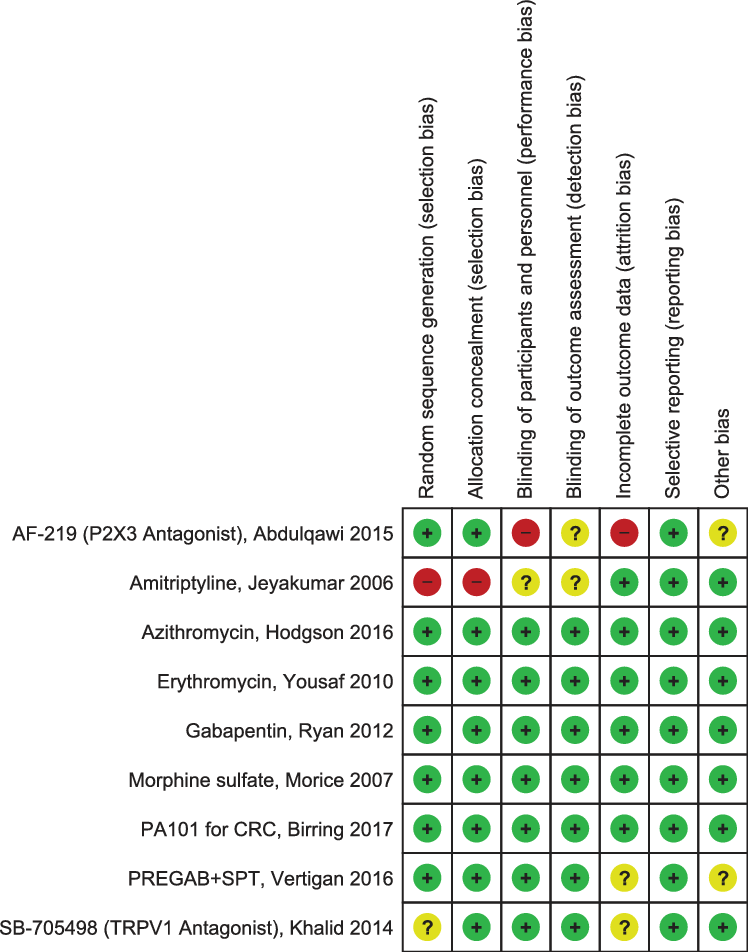 | 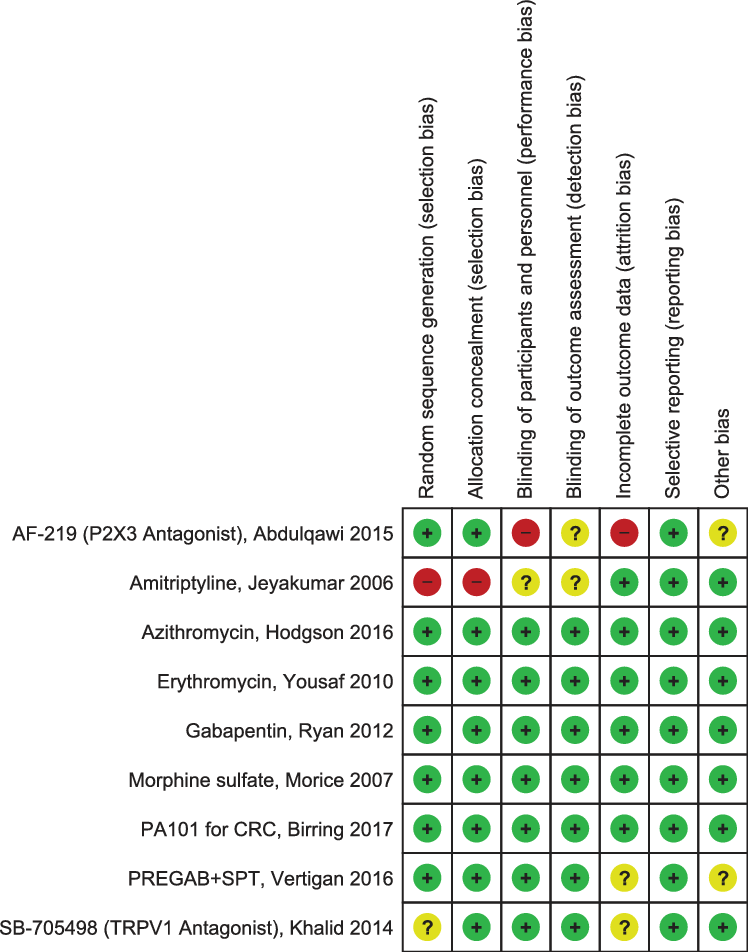 | 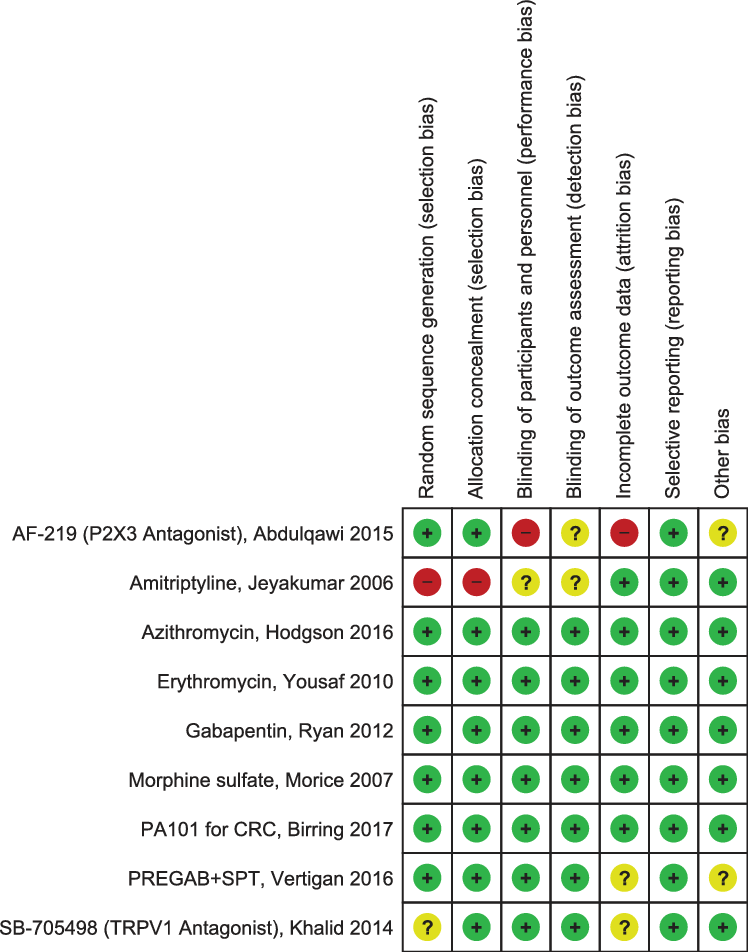 | 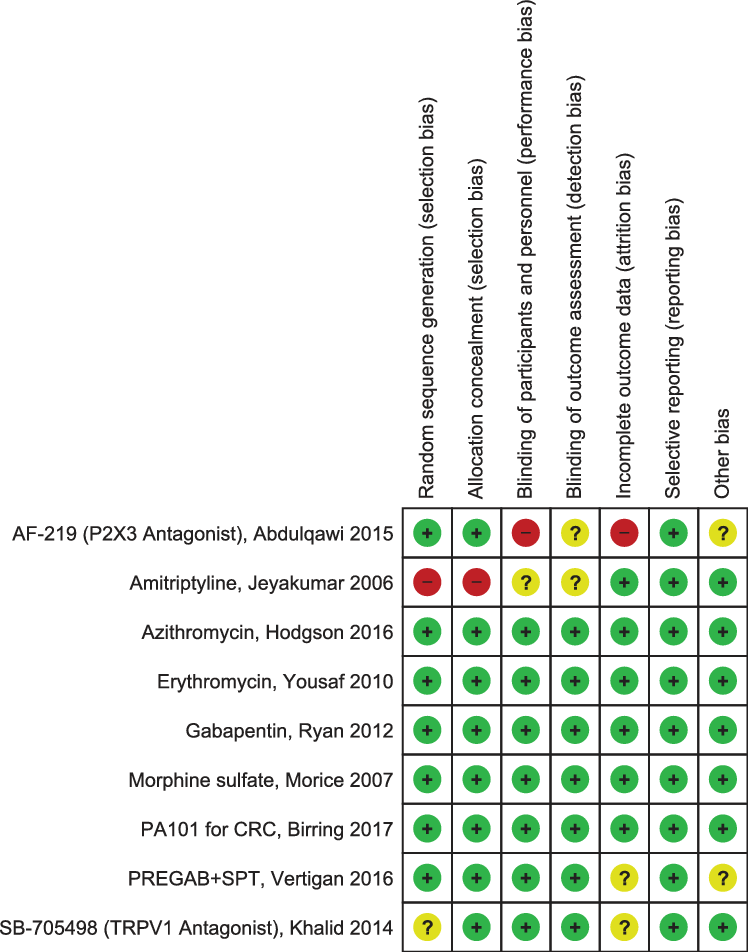 | 10 |
| Alfarra et al., 2022 | Non-RCT | 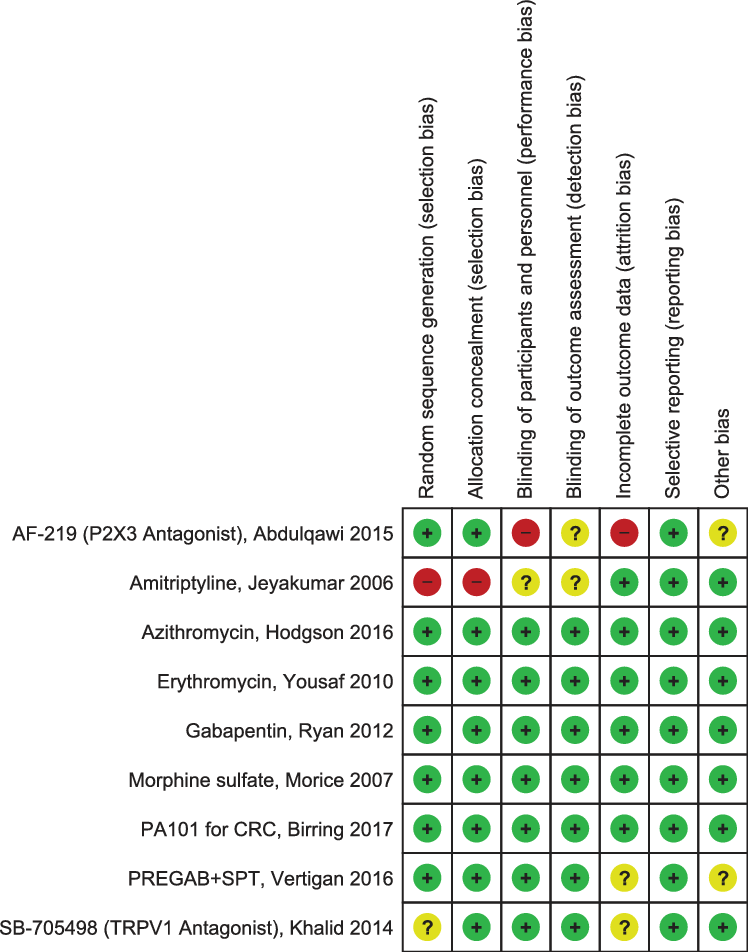 | 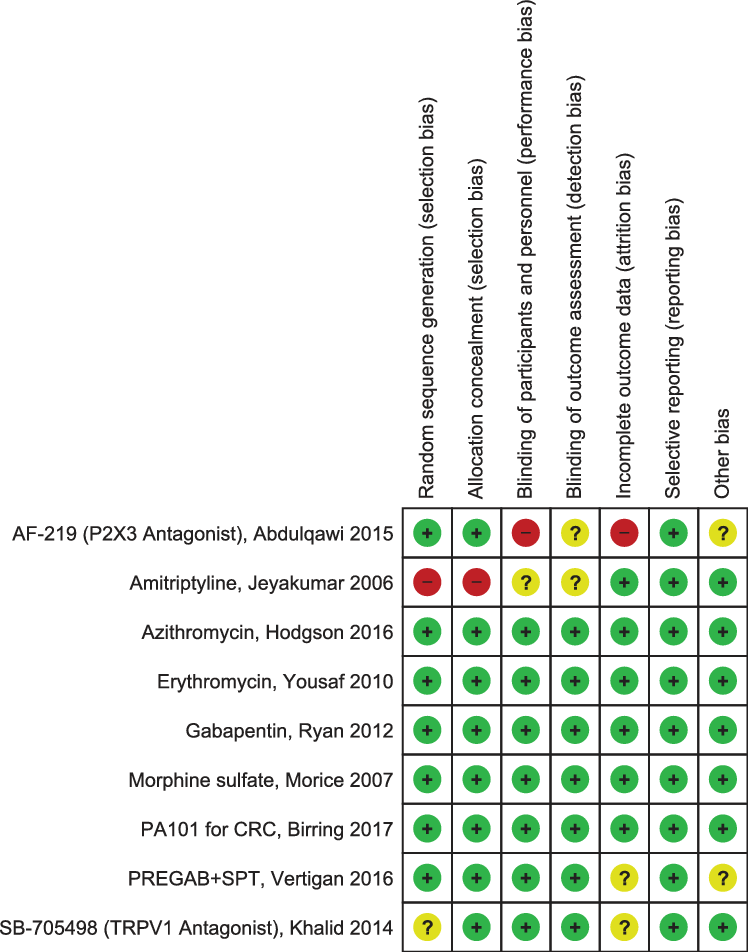 | 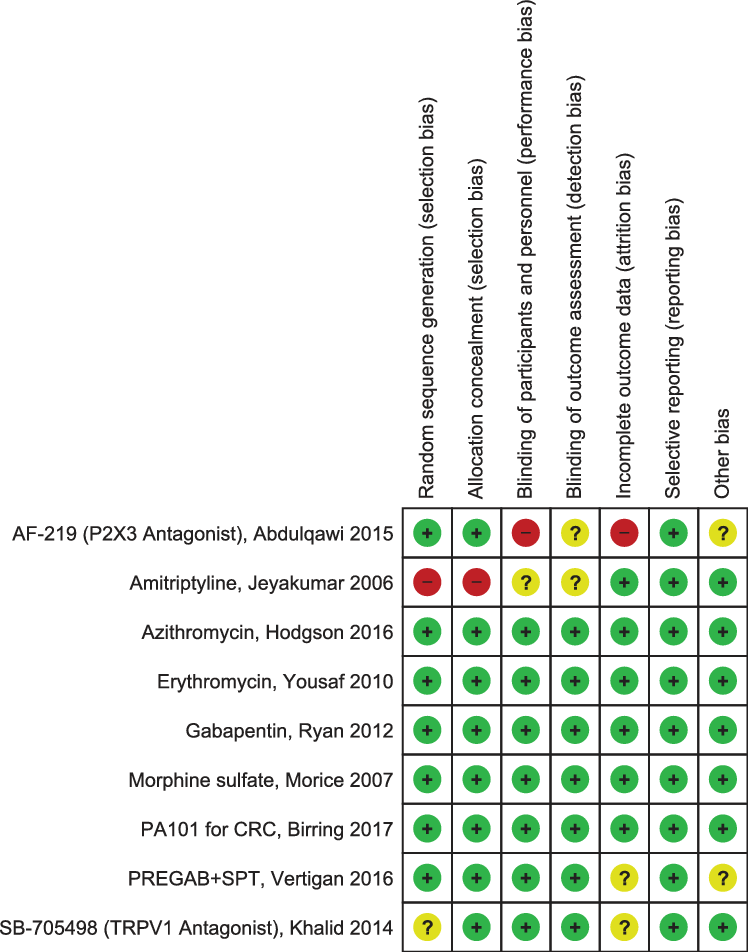 | 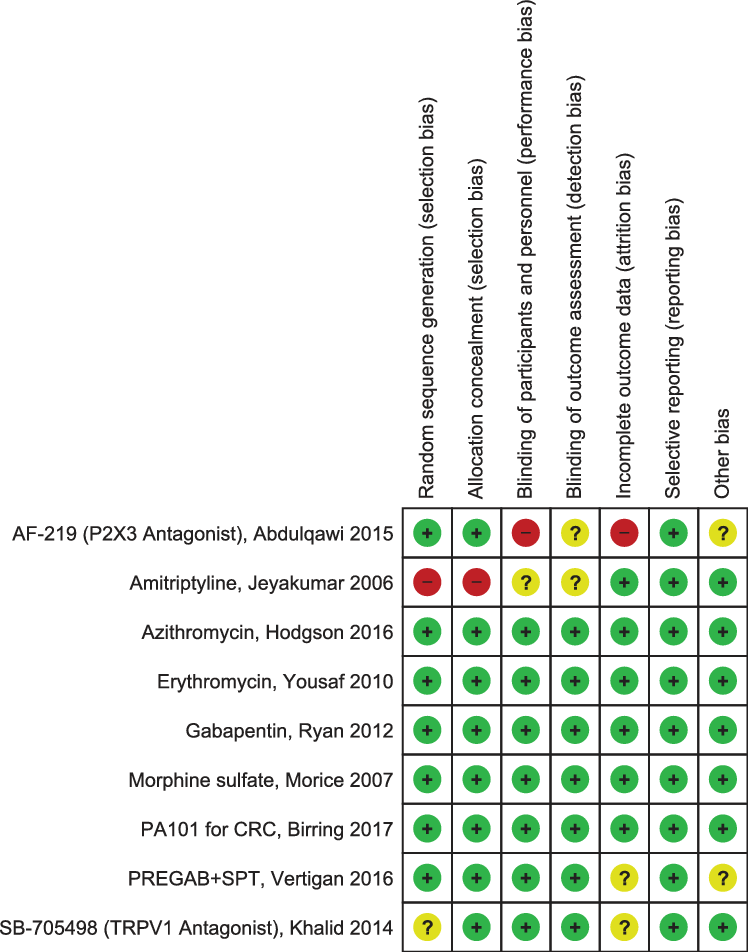 | 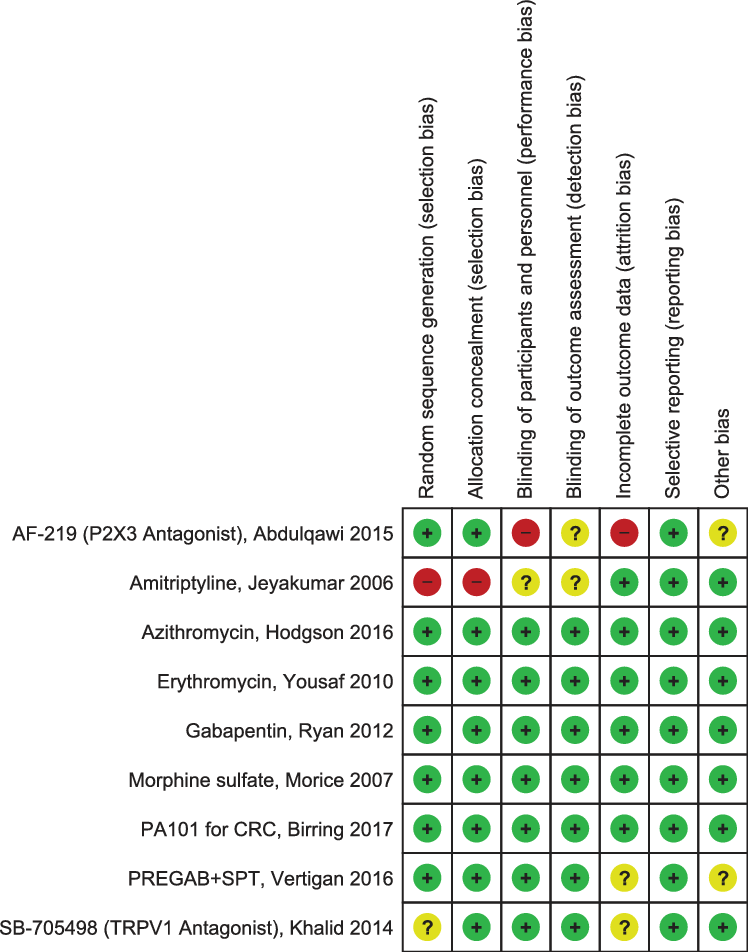 | 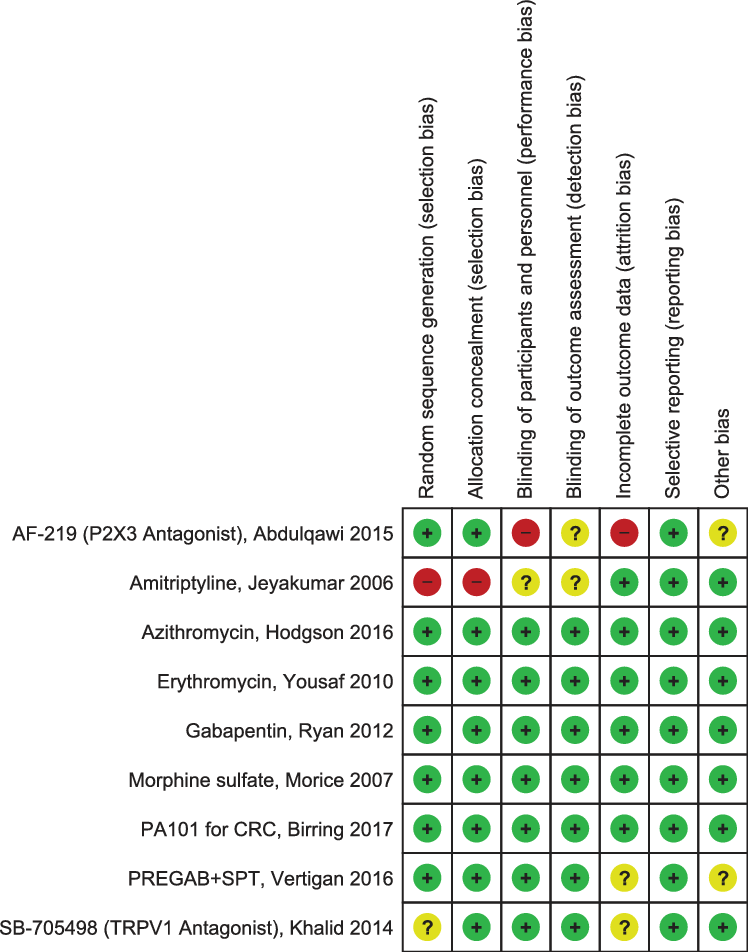 | 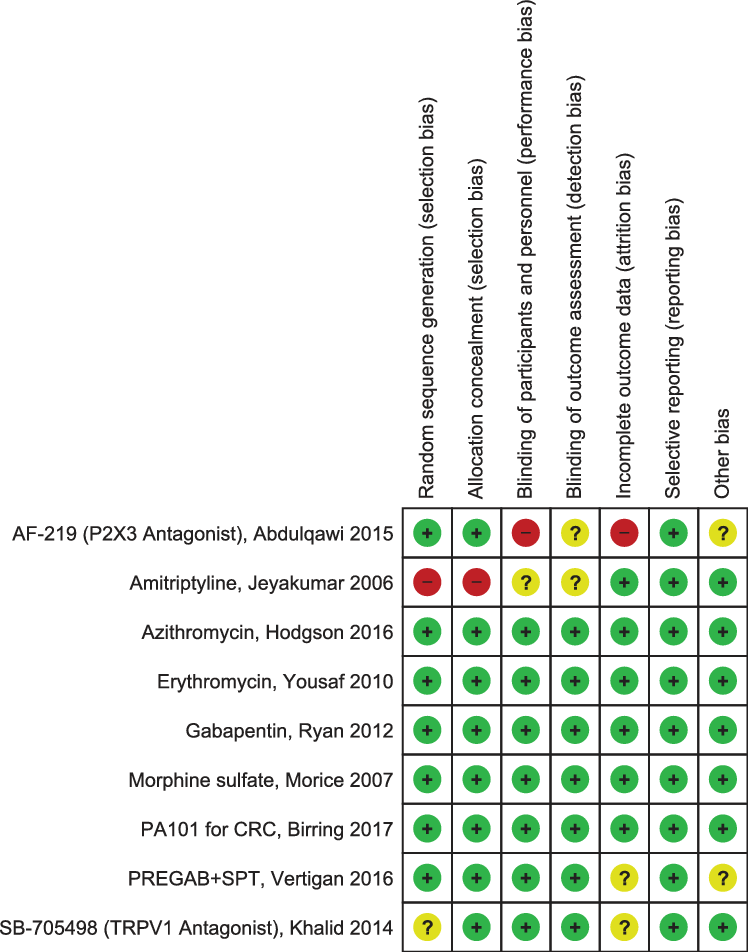 | 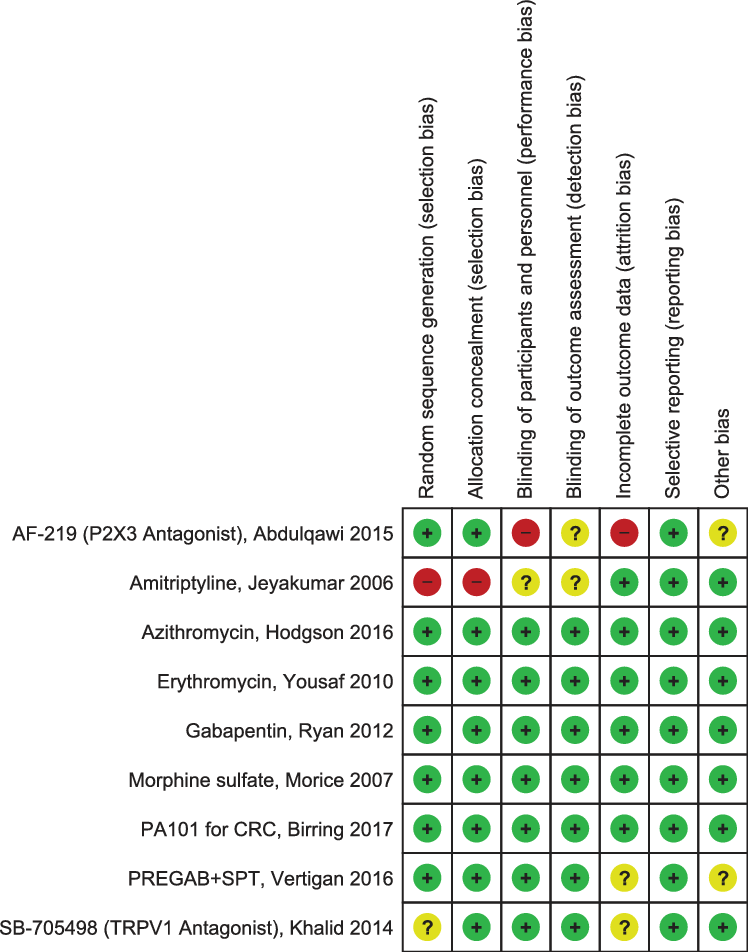 | 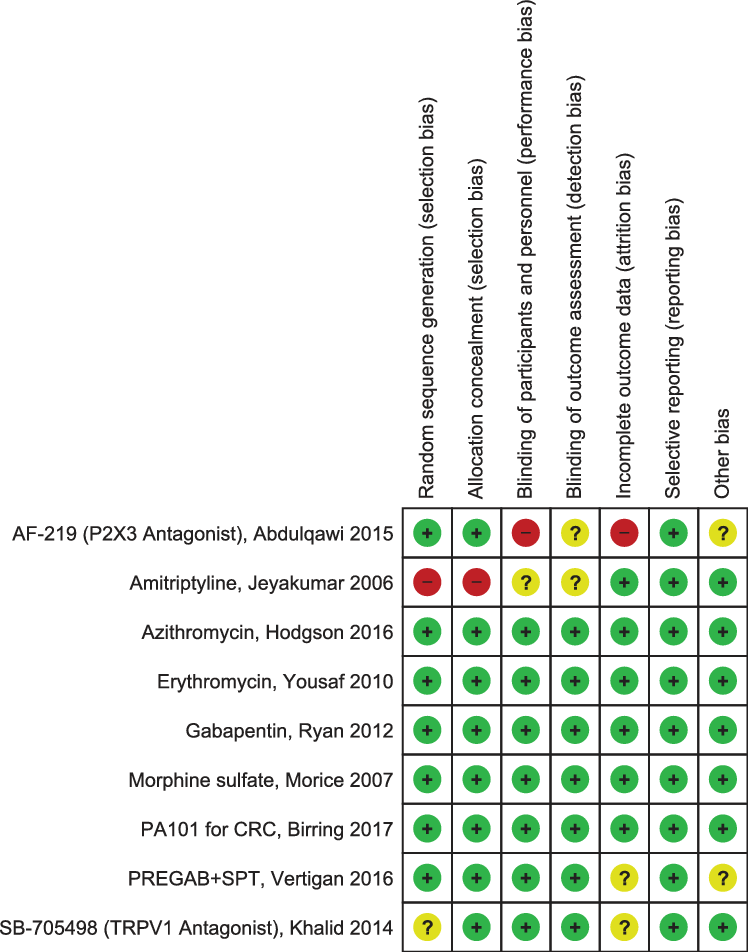 | 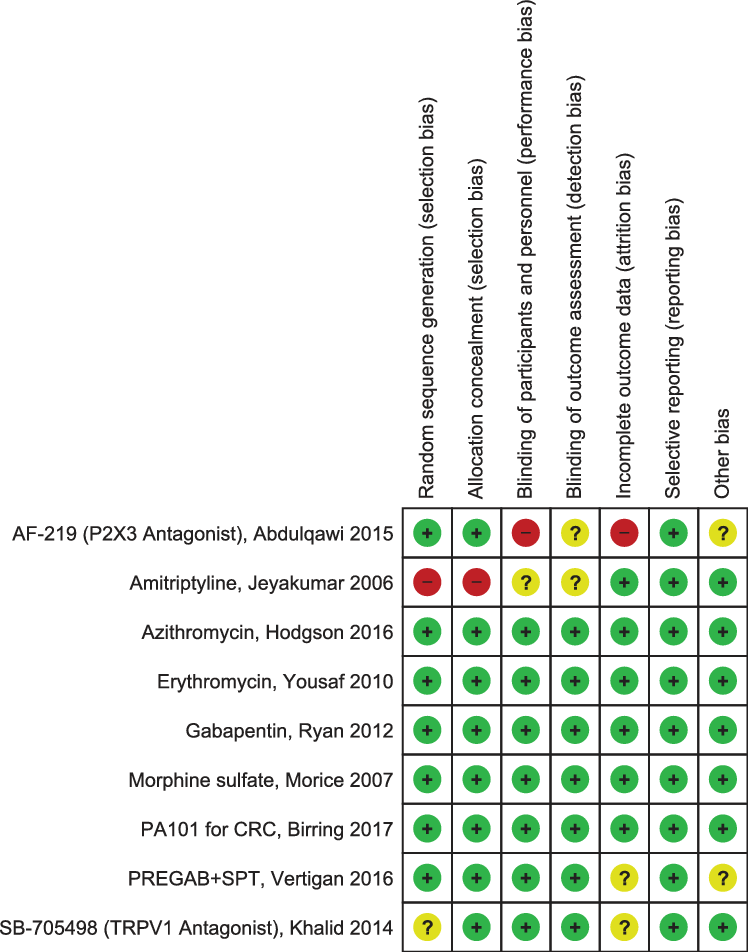 | 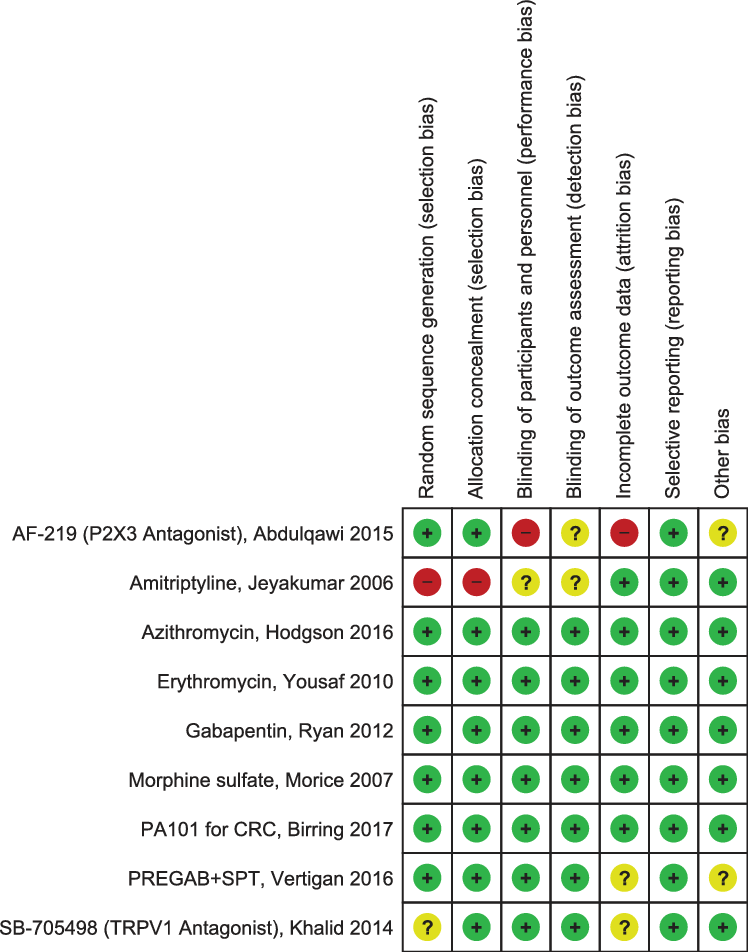 | 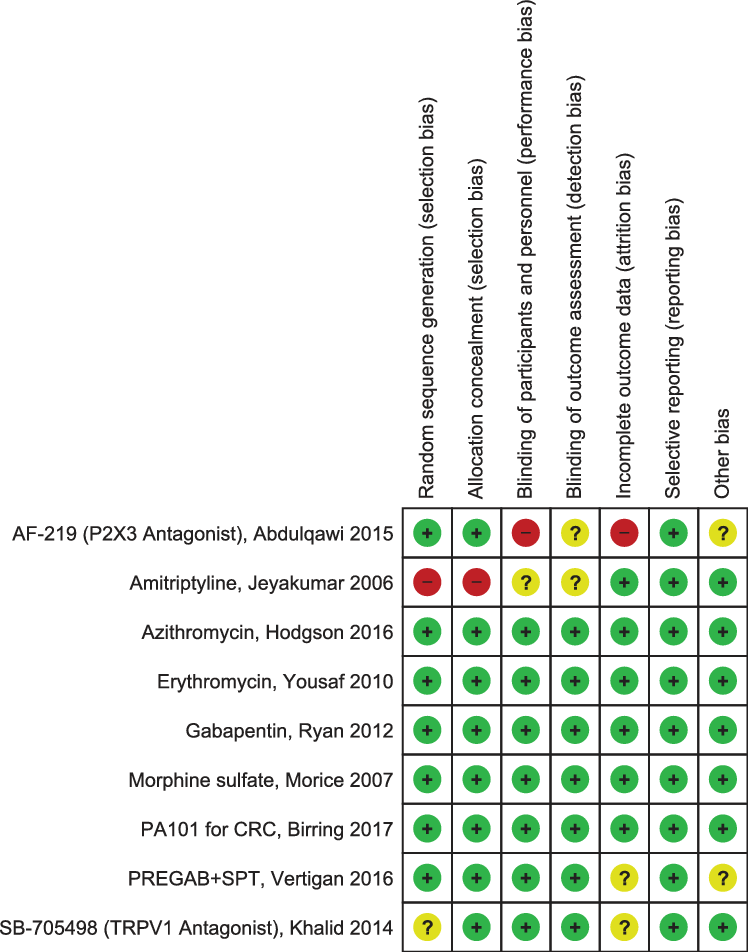 | 7 |
| Colombage et al., 2023 | Non-RCT | 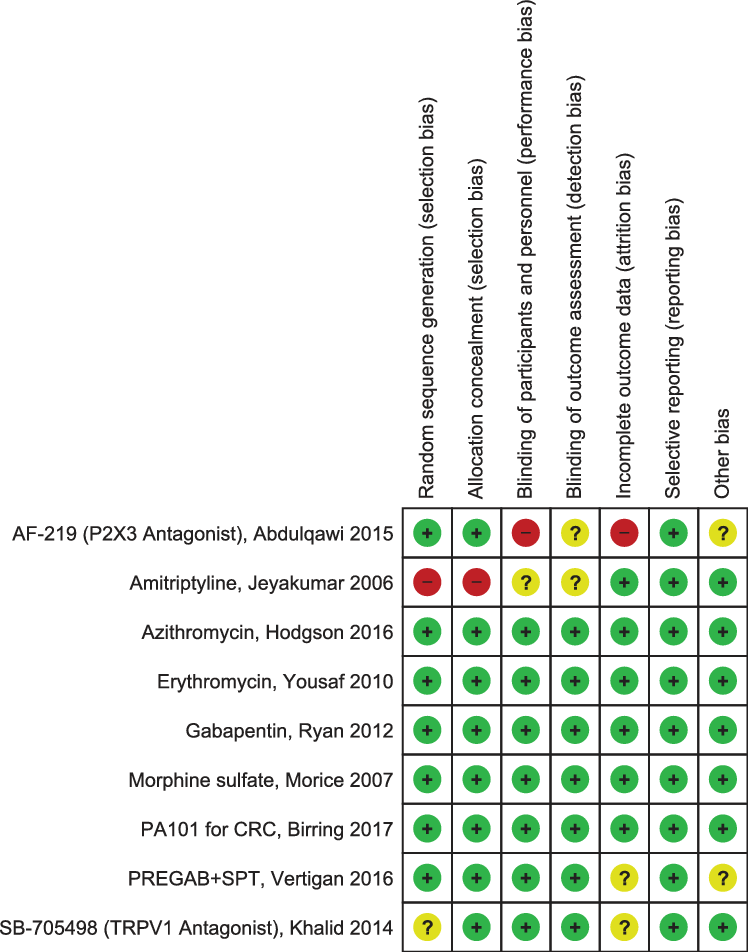 | 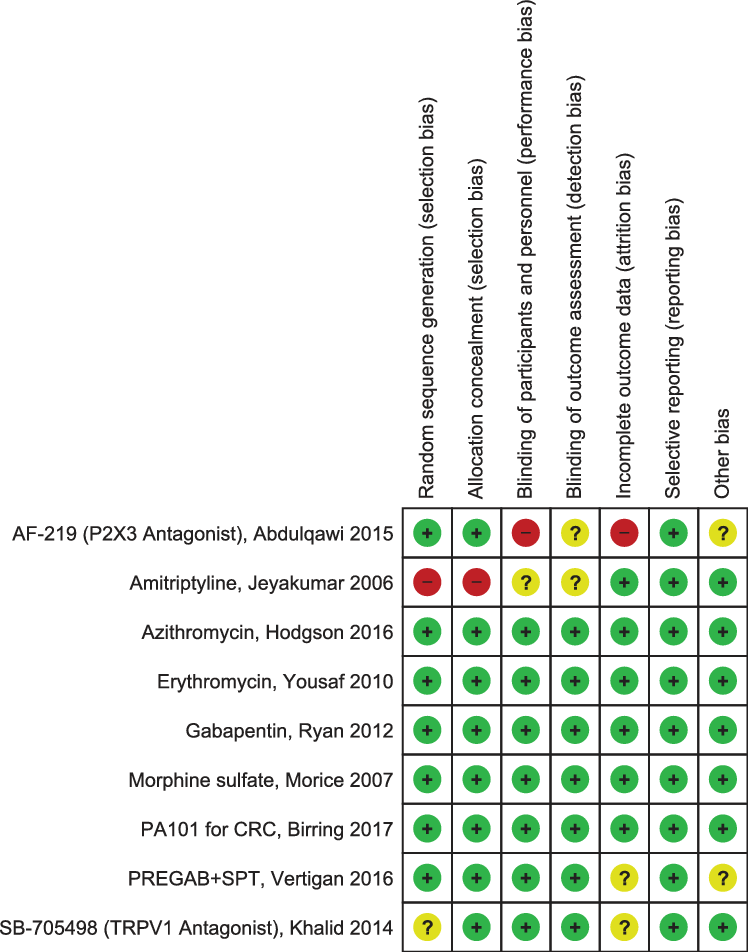 | 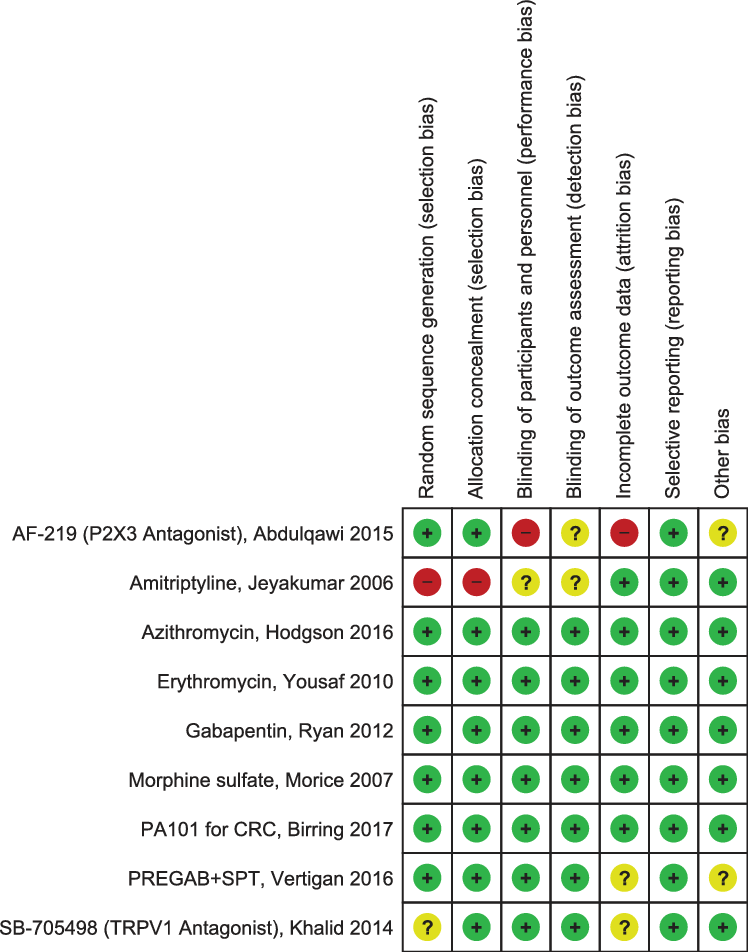 | 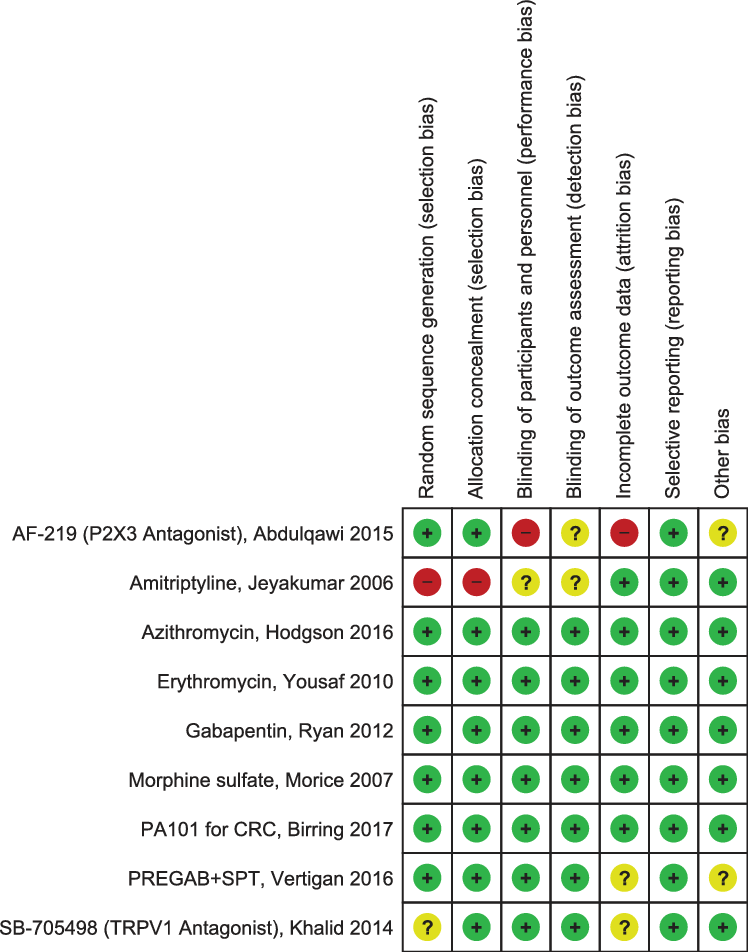 | 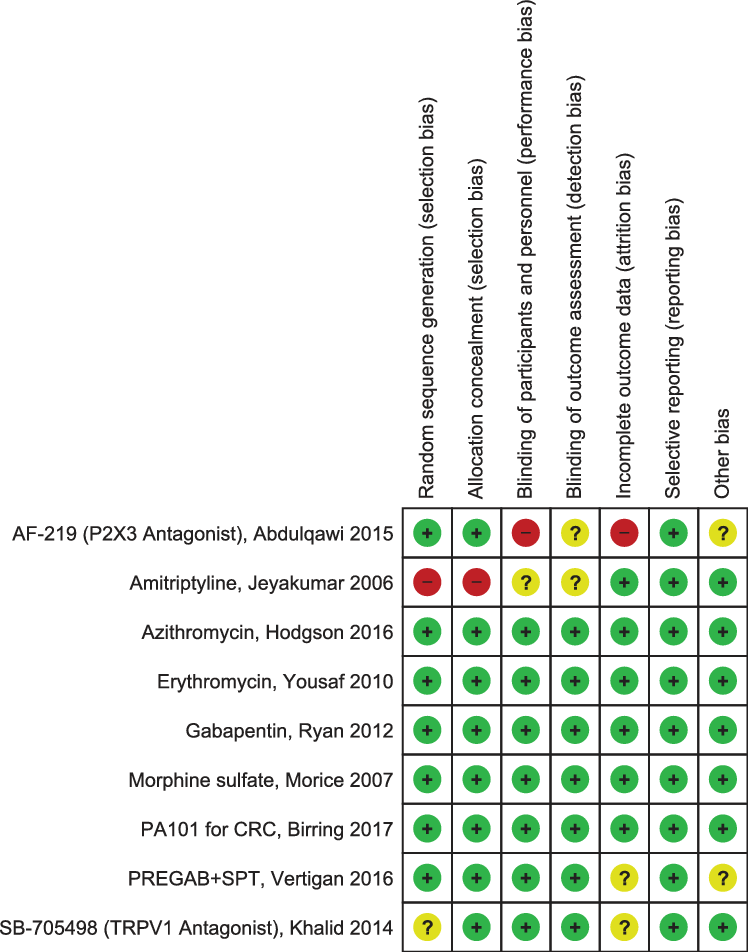 | 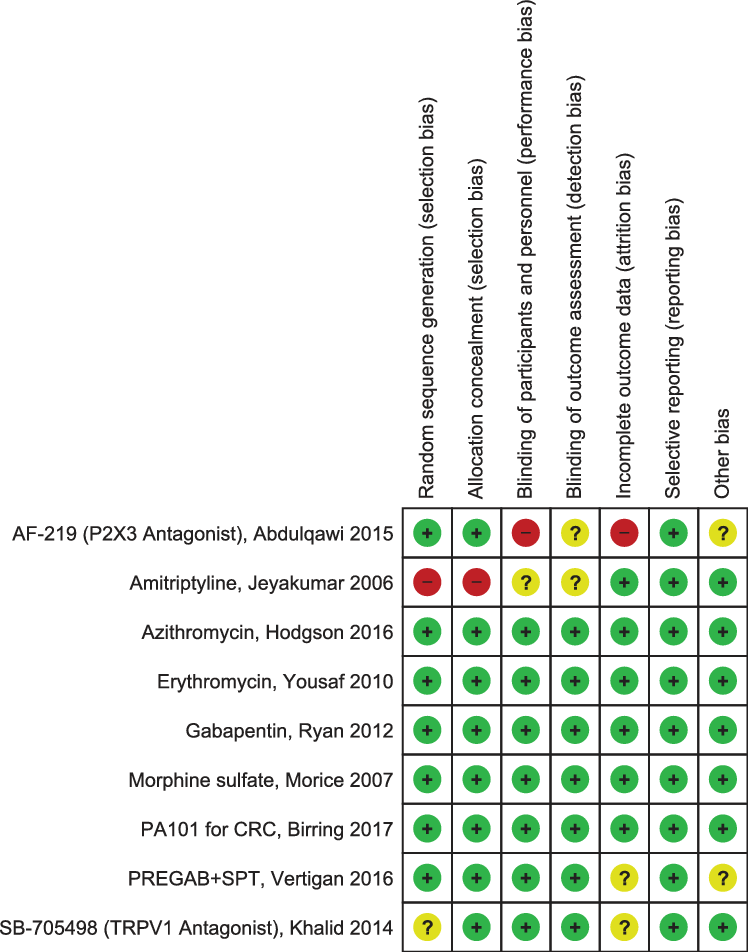 | 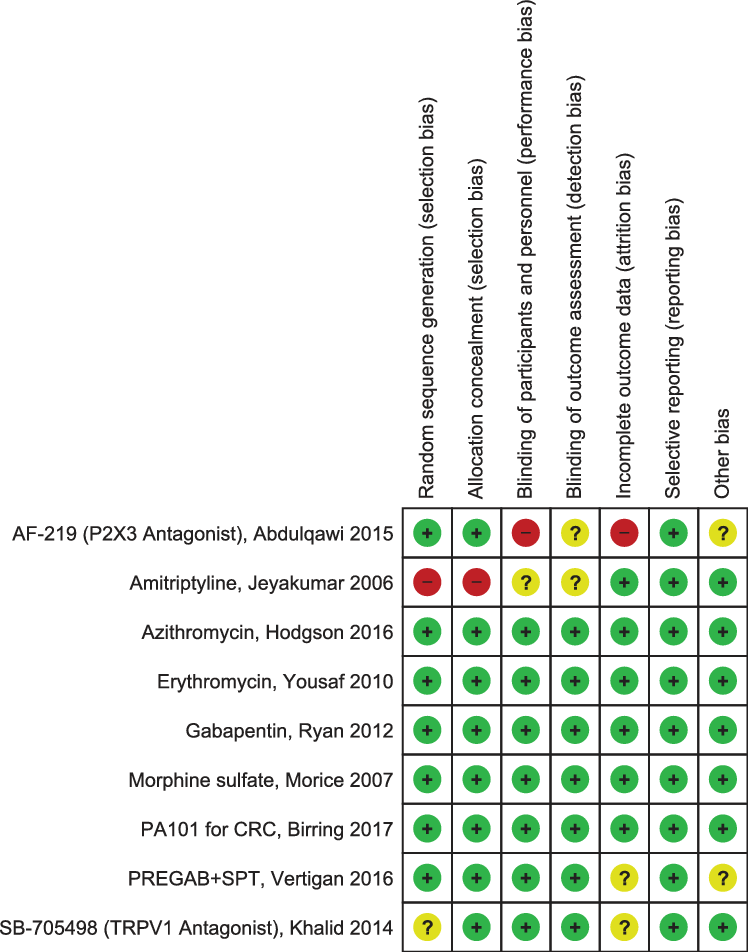 | 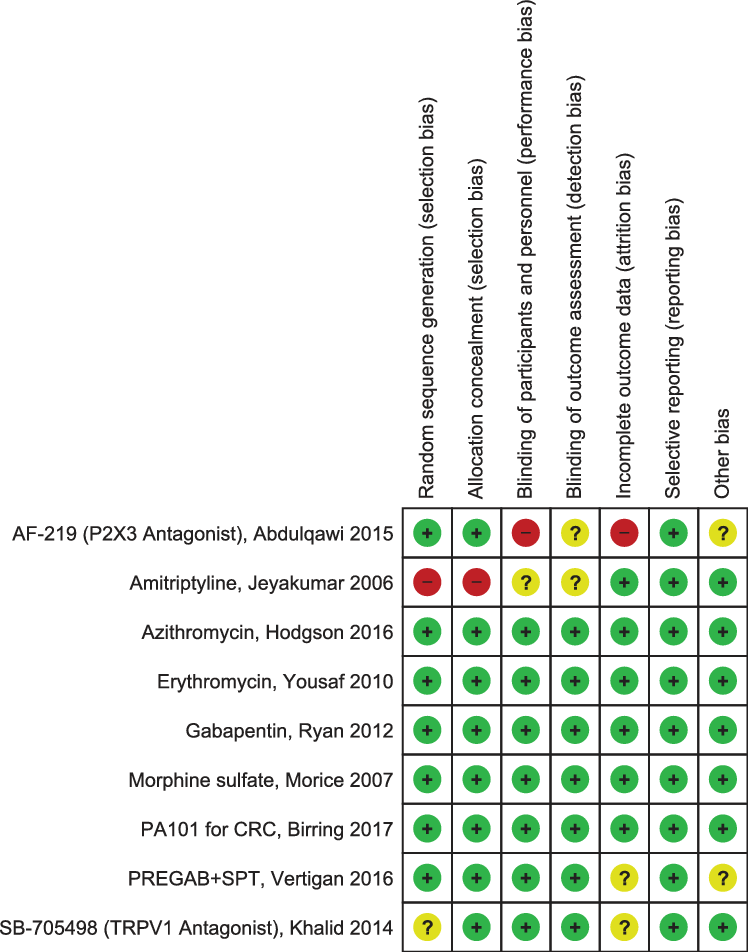 | 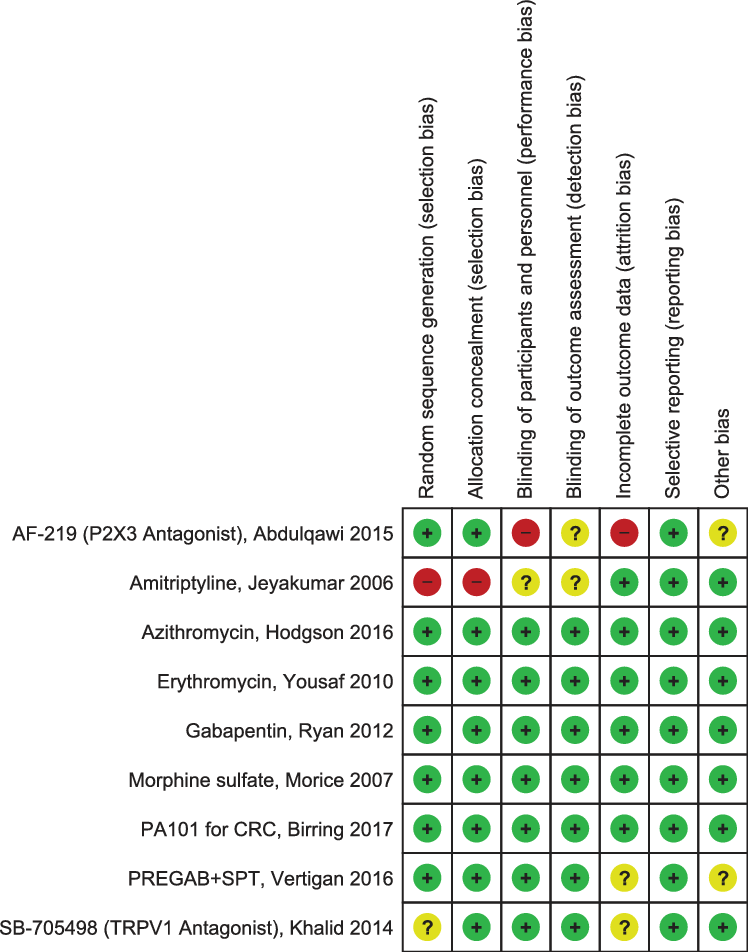 | 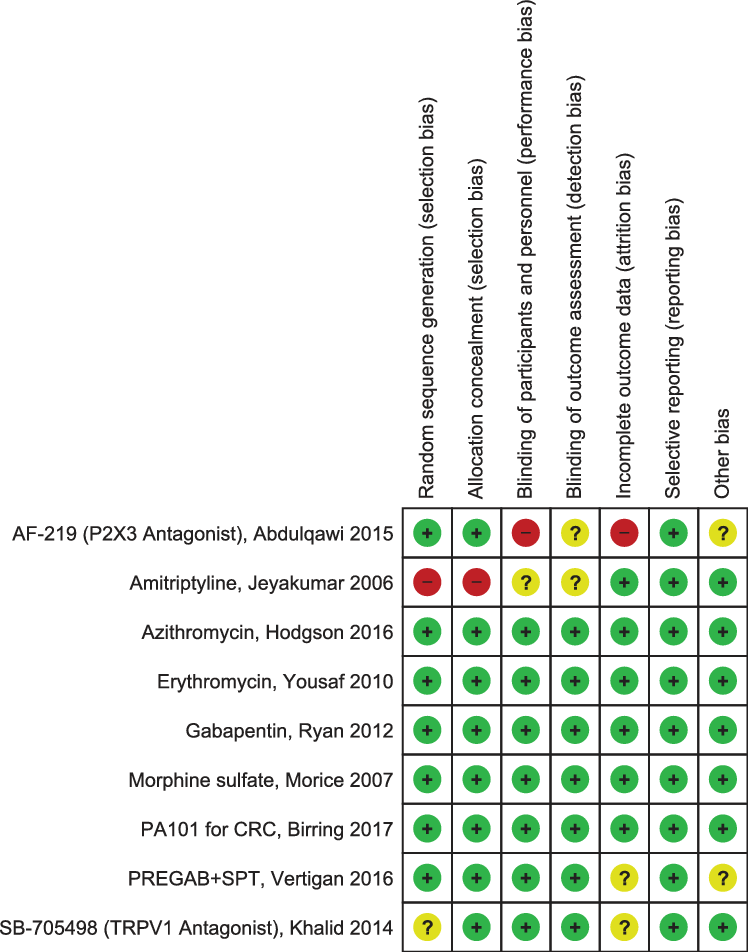 | 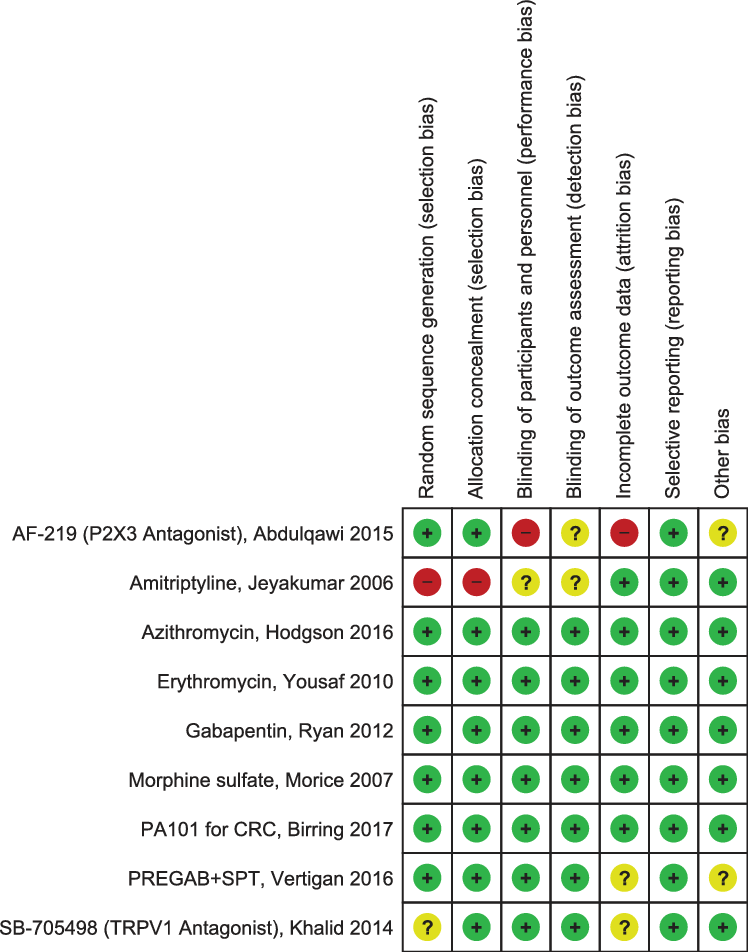 | 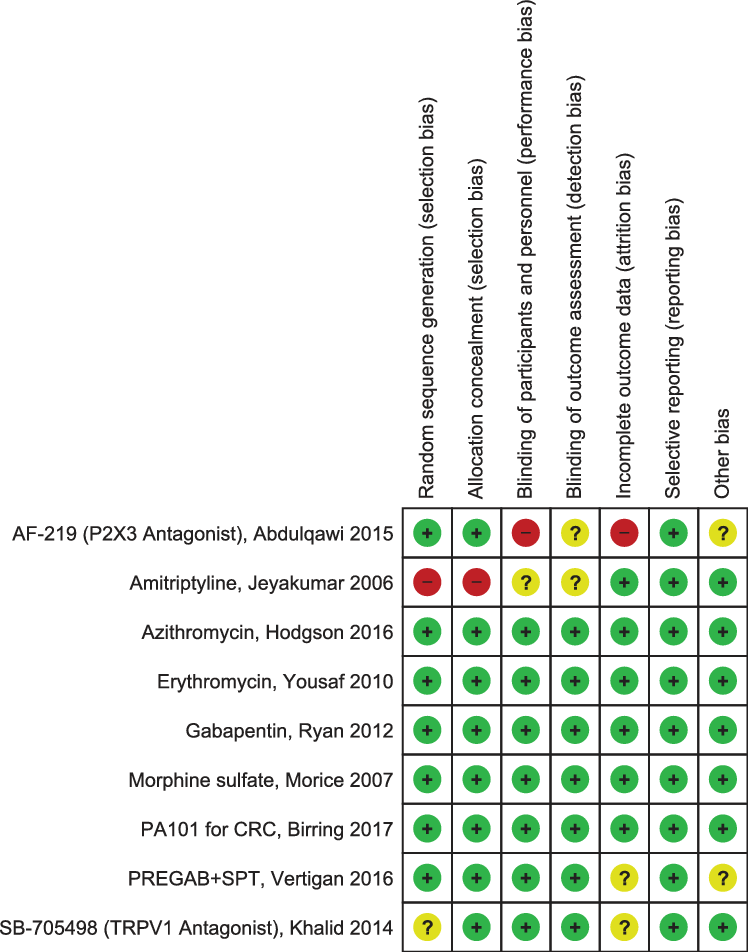 | 11 |
| Juraskova et al., 2013 | Non-RCT | 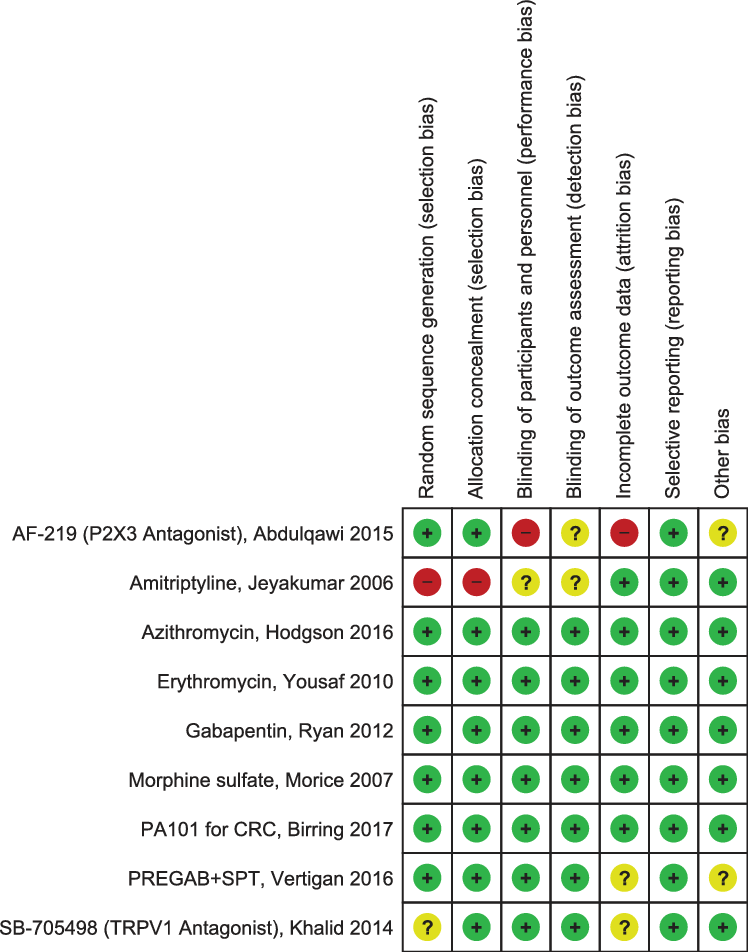 | 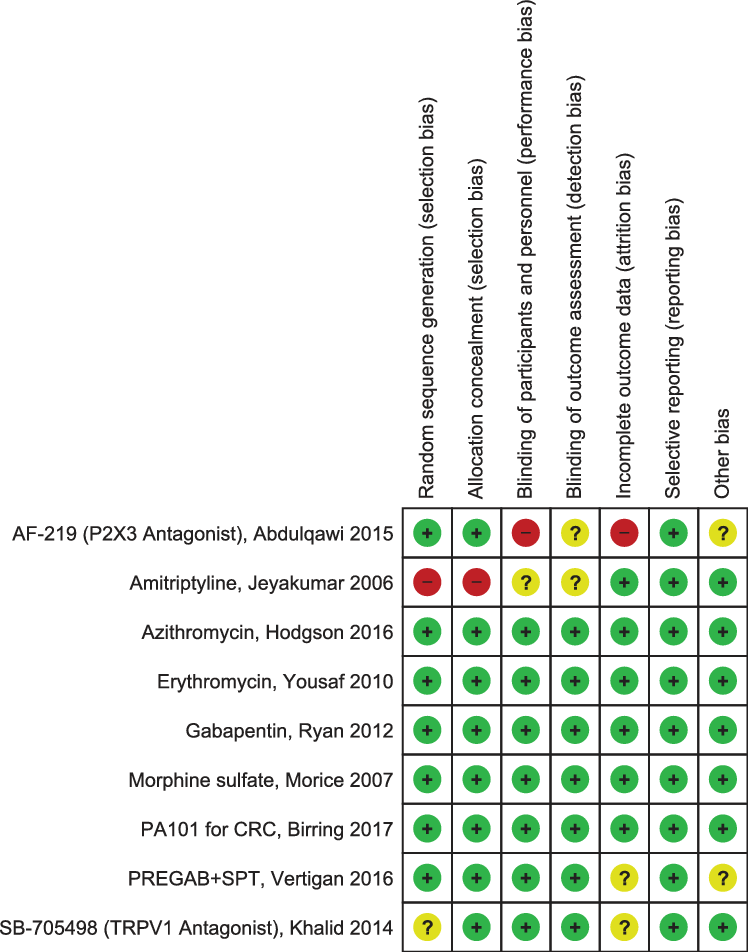 | 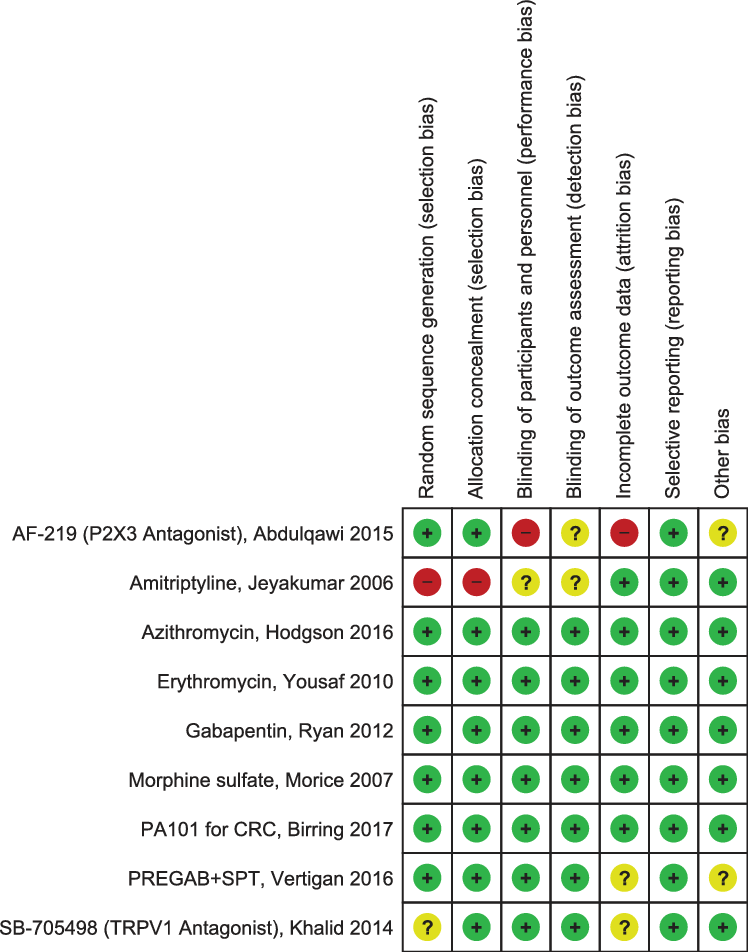 | 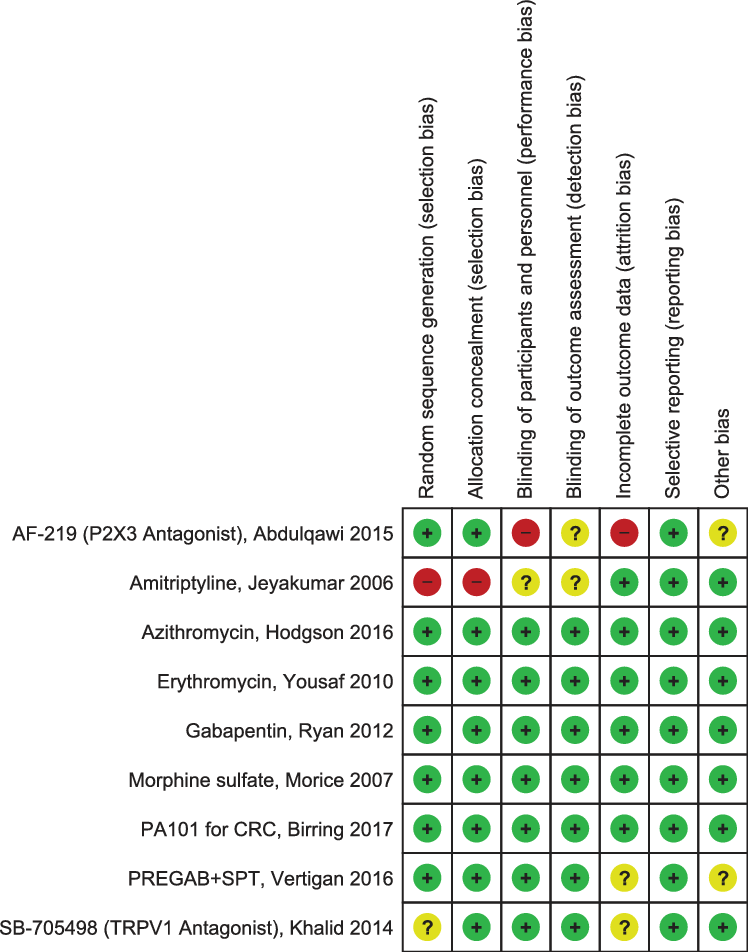 |  |  |  |  |  |  |  |  | 9 |
| Bokaie et al., 2022 | Non-RCT |  |  |  |  |  |  |  |  |  |  |  |  | 6 |
| Bober et al., 2019 | Non-RCT |  |  |  |  |  |  |  |  |  |  |  |  | 9 |
| **Mixed cancer populations: 1 RCT** | | | | | | | | | | | | | | |
| Shover et al., 2013 | RCT |  |  |  |  |  |  |  |  |  |  |  |  | 10 |

RCT: randomized controlled trial, TIDieR: Template for Intervention Description and Replication

(b) Detailed TIDieR Checklist

| **Author, year** | **Name of intervention** | **Rationale** | **Materials** | **Procedures** | **Provider** | **Mode of delivery** | **Location** | **Intervention dosage** | **Tailoring** | **Modifications** | **Planned intervention adherence & strategies for adherence** | **Actual intervention adherence** |
| --- | --- | --- | --- | --- | --- | --- | --- | --- | --- | --- | --- | --- |
| Advani et al., 2017 | Combination of sexual counseling, vaginal moisturizers, lubricants, dilation, and PFM exercises | Based on previous work, it was hypothesized that a combination of sexual counseling, vaginal moisturizers, lubricants, dilation, and PFM exercises would prevent further deterioration of sexual function, compared to usual care. | Study booklet (Why is it important to take your aromatase inhibitor), vaginal moisturizer (two types), water-based lubricant, silicone vaginal dilator, (Soulsource®1in.×4in.), website | Women were instructed to use the moisturizer daily during week 1, and then 2–3 times per week. Women were advised to have penetrative sex with a partner and/or to use the dilator at least 2 times per week (with vaginal lubricant). All women received access to the website providing detailed help with women’s cancer-related sexual problems. 6 phone coaching calls were scheduled during the 12-week treatment period, plus 3-monthly follow-up calls. Calls lasted 15–30 min and included standard questions on frequency of sex, use of the moisturizer, lubricant, and dilator, satisfaction with the vaginal moisturizer, and bother with genital irritation, hot flashes, or joint pain/stiffness problems. | NR | Online (website) & telehealth (coaching calls) | Unclear  Assume home-based. Patients recruited from UT MD Anderson Cancer Centre. | Moisturizer daily during week 1, and then 2–3 times per week. Women were advised to have penetrative sex with a partner and/or to use the dilator at least 2 times per week with water-based vaginal lubricant. 6 phone coaching calls were scheduled during the 12-week treatment period, plus 3-monthly follow-up calls. Calls lasted 15–30 min. | Generic | NR | At follow-up, items assessed whether a woman had discontinued her aromatase inhibitor and her daily adherence in the past 2 weeks.  At each phone call, women were asked if they had used the web intervention. | Out of 9 planned calls, the mean number achieved was 5.71±2.76.  19 (54%) never used the web intervention, 10 (29%) used it once, and 6 (17%) more than once. |
| Fatehi et al., 2019 | Psychosexual counseling | This study was mainly based on Ganz and colleagues’ conceptual framework that proposes that breast cancer and its treatment influence sexual quality of life. In this study, a sexual quality of life improvement intervention was proposed to maintain couple relationship, coping skills and improve sexual function which may ultimately lead to a change in sexual quality of life. | NR | The intervention consisted of 6 x 90-120 min weekly counseling sessions. The content of the sessions consisted of the initial meeting and familiarity, describing the anatomy of sexual organs and sexual cycles in both genders, the consequences of breast cancer and its treatment effects on sexual relationships and the relationship with the spouse, and providing solutions based on Master and Johnson’s sex therapy principles to deal with sexual problems. | NR | NR  Eligible patients were invited to attend a meeting in Educational-Medical Centre of Imam Khomeini Hospital in Sari city to become familiar with the type and purposes of the study.  Unclear how the counseling sessions were delivered. | NR or unclear  Recruitment: cancer centre located in Imam Khomeinin Hospital in Sari city, North of Iran.   Eligible patients were invited to attend a meeting in Educational-Medical Centre of Imam Khomeini Hospital in Sari city to become familiar with the type and purposes of the study. Unclear where the counseling sessions were delivered. | 6 x 90-120 min weekly counseling sessions | Unclear | NR | NR | NR |
| Hummel et al., 2017; 2018 | Internet-Based Cognitive Behavioral Therapy (CBT) | There is a range of effective psychological interventions for sexual dysfunctions, with F2F CBT generally considered to be the gold standard. There is growing evidence that Internet-based CBT is an effective method of treatment for a variety of psychological problems, including sexual dysfunctions. | Internet-based CBT that included standardized information texts and homework assignments. The client had access to the therapy program using a personal account via a secured, password-protected Web site. | The Internet-based CBT was composed of ~20 weekly sessions to be completed within a maximum of 24 weeks.  Before the start of the CBT, the therapist and client formulated therapeutic goals, and these were included in the treatment plan. The sexologist selected 4-5 modules that suited the sexual problems best. Each module consisted of multiple interventions, varying between 4-8 interventions per module. Each intervention consisted of standardized information texts, homework assignments, a report to the sexologist, and feedback from the sexologist. The sessions did not take place in real time, but rather consisted of an extensive reply from the therapist via email in response to the completed homework assignments. Involvement of the partner was desirable, but not mandatory.  Two evaluation interviews were scheduled by telephone, 1 halfway through and 1 at the end of therapy. During these interviews the therapist reviewed with the client the extent to which goals had been achieved and set future goals (including maintenance of progress made after the end of therapy). | Female psychologists or sexologists.  All psychologists had a Master of Science degree in clinical psychology and extensive experience in the treatment of sexual dysfunctions. 3 were registered as sexologists at the Dutch Scientific Association for Sexology. 1 of the psychologists was a registered health psychologist. All psychologists or sexologists had undergone special training in the sexual issues of breast cancer survivors and the application of the Internet-based CBT program. | Modules and interventions: Internet-based and individual, involving written communication (via email) between participants and therapists.  Telephone evaluation sessions (2 - halfway through and at the end of therapy). | Internet-based.  Recruitment: 10 hospitals in the Netherlands. | The Internet-based CBT was composed of ~20 weekly sessions that had to be completed within a maximum of 24 weeks. | Before the start of the CBT, the therapist and client formulated therapeutic goals, and these were included in the treatment plan. The sexologist selected 4-5 modules that suited the sexual problems best.   The CBT was tailored to the needs of the individual, including the choice of modules and homework exercises and the frequency of contact.  There was room for the therapist and client to tailor the timing between sessions (e.g., if needed, the next session could be delayed). | NR | Adherence to the intervention was one of the major, recurring items on the agenda for the weekly meetings of the psychologists or sexologists. If a week passed without client activity, a reminder was sent via email by the therapist. If another week passed, the therapist telephoned the client. To improve adherence to therapy, 2 motivational calls were scheduled by the therapist: one during the first half and one during the second half of the CBT (these calls took place in addition to the telephonic evaluation interviews). In the case of more serious problems with therapy adherence, the therapist telephoned the client to discuss the cause and possible solutions. | The CBT was successfully completed (according to the judgment of the therapist) by 61.9% of women (mean duration of CBT, 22.1 weeks; standard deviation [SD], 4.5); 31.0% ended the CBT prematurely (mean duration of CBT, 9.6 weeks; SD, 5.8), and 7.1% never started the CBT. The most common reasons for attrition were time constraints (25.6%), the intensity of the CBT (20.5%), and personal circumstances (12.8%) or relationship problems (7.7%). |
| Duijts et al., 2012 | (1) CBT (+ relaxation exercises); (2) physical exercise (aerobic); (3) CBT + physical exercise | There is growing evidence that CBT and physical exercise can have a positive impact on vasomotor symptoms in naturally occurring menopause. It has been hypothesized that the positive effect of exercise on vasomotor symptoms may be because of elevated levels of endorphins that regulate central thermoregulation. | Heart rate monitor (CBT + physical exercise group) | CBT program: consisted of 6 x 90-min weekly group sessions, including relaxation exercises. The primary focus of the CBT was on hot flashes and night sweats, but other symptoms and problem areas were also addressed. A booster session was held 6 weeks after completion of the program.  Physical exercise program: 12-week, individually tailored, home-based, self-directed, exercise program of 2.5-3 hours per week. During the intake session, the physiotherapist assisted each woman in selecting an appropriate form of exercise. Each woman was provided with a heart-rate monitor and was instructed in its use to achieve a target heart rate (60-80% intensity using the Karvonen formula). In weeks 4 and 8, women had telephone interviews with the physiotherapist to discuss their experience and the possible need to adjust the program. In the last week, women visited the clinic for a final session during which they received advice on how best to maintain the desired level of physical activity.  Women in the combined intervention group underwent the CBT + physical exercise concurrently. | CBT: clinical psychologist and clinical social workers experienced in counseling women with breast cancer and specially trained in administering CBT.   Physical exercise: specially trained physiotherapists. | CBT: group sessions, supervised, F2F.  Physical exercise: individual, self-directed (unsupervised). Telephone interviews in Week 4 and 8. Last week - women visited a clinic for a final session.   Physical exercise intake session: supervised, F2F, and assume individual. | CBT: unclear (carried out at 4 locations). Assume hospital recruitment site but not explicitly stated.  Physical exercise: home-based. Intake session: unclear (carried out in 6 locations). Assume hospital recruitment site but not explicitly stated.   Recruitment: hospitals in the Amsterdam and Rotterdam regions of the Netherlands. | CBT: 6 x 90-min weekly group sessions, with a booster program held 6 weeks after completion of the program.  Physical exercise: 12 weeks, 2.5-3 hours of exercise per week at target heart rate (60-80% Karvonen). | Physical exercise: individually tailored. Each woman was assisted by a physiotherapist to select an appropriate form of exercise. | NR | Program compliance was assessed via session attendance records for CBT participants and number and intensity of training sessions, as recorded by the heart rate monitor for CBT + physical exercise participants.   Participants were considered compliant if they attended at least 4 CBT sessions and/or if they had a minimum of 24 physical exercise training sessions, with an average of 3 kilocalories burned per kilogram per session, that is, a metabolic equivalent of 6. | High levels of undercompliance were observed for all 3 interventions. 58% of the CBT group, 64% of the physical exercise group, and 70% of the combined group did not meet criteria for compliance. |
| Schover et al., 2011 | Sisters Peer Counseling in reproductive issues after treatment program | In a pilot study, we partnered with the national advocacy organization Sisters Network Inc. to create a pilot intervention, Sisters Peer Counseling in Reproductive Issues After Treatment (SPIRIT), which was designed to improve knowledge and reduce symptoms related to sexual dysfunction, menopause, and distress about infertility in African American breast cancer survivors. Given the positive results, a national trial of the SPIRIT program, again in partnership with Sisters Network Inc. was conducted. | SPIRIT workbook, prepaid telephone card, counsellor developed local and national reproductive health resource list, treatment manuals for counseling sessions. | For the peer counselled group, a counsellor met with the participant for 3 x 60-90 min sessions during the treatment period (in the participant’s home or at a site like a church or community centre). Each session focused on 1 chapter of the workbook. Counsellors sometimes travelled overnight to accommodate participants who lived in remote areas, conducting 3 sessions within a period of 1-2 days.  The telephone counseling group’s packet included the workbook, the counsellor’s contact information, encouragement to call the counsellor for up to 30-min to discuss issues in the SPIRIT workbook, and a prepaid telephone card. The counsellor did not initiate any contacts in the telephone condition unless the Houston team identified a need; for example, a questionnaire result might trigger a telephone assessment of psychological distress. | Trained peer counsellors (attended a 5-day counsellor training conference) | Peer counselled group: individual, supervised, F2F.   Telephone group: individual, supervised, telehealth. | Peer counselled group: participant’s home or at a site like a church or community centre.  Telephone group: telephone. | Peer counselled group: 6-week treatment period, 3 x 60-90-min sessions.  Telephone group: 6 weeks, patient encouraged to call the counsellor for up to 30-min to discuss issues in the SPIRIT workbook. | Each chapter began with a list of topics (for example, ‘‘Learning more about ways to overcome vaginal dryness’’). The survivor rated the personal importance of each topic before each counseling session, which helped to tailor the focus according to factors like patient age, concerns about sexuality or childbearing, etc. | NR | Seven counsellors were appointed as regional managers. Managers acted as counsellors but received additional compensation for weekly quality-control duties, which included checking by telephone with assigned counsellors on recruitment efforts and counseling sessions and reviewing mental health or medical concerns.  Telephone group: The counsellor kept a log of the date, time, duration, and topic of any calls from the participant. | In the telephone counselled group, only 22% of women called their counsellor during the 6-week intervention period. The mean total number of min on the telephone with the counsellor was 28 (17) min. |
| Zangeneh et al., 2023 | Sexual education based on the Ex‑PLISSIT model | Some studies have shown that sexual education based on the Ex‑PLISSIT model can improve women’s sexual function and sexual satisfaction with chronic diseases, such as diabetes and multiple sclerosis, and in reproductive age. But there are limited studies on women undergoing breast cancer treatment. | Education booklets, online group through a virtual network | The training included 4 x 60-90 min session that were based on the four stages of the Ex‑PLISSIT model: permission (P), limited information (LI), specific suggestions (SS), and intensive therapy (IT). The education sessions included measures to solve common sexual problems after cancer treatment, relaxation techniques, breathing exercises, Kegel exercises, and managing body image. | Study researchers who had completed the sexual education courses. The content of the training sessions was prepared and implemented under the supervision of a member of the research team who specialized in sex therapy. | Online, group-based education | Online: The intervention group was informed about the days and hours of the sessions through the online group. The link to join the meeting was sent to the online group members on the morning of the meeting.   Recruitment: Isfahan branch of cancer prevention and control centre. | 4 x 60-90 min education sessions (one per week for 4 weeks) | Generic with opportunity for individual questions. During the online education sessions, in addition to providing educational materials, questions raised by group members were answered, and participants shared their experiences. The researcher answered questions of the participants and sent messages for them through the virtual network. | NR | NR | 100% - participants were excluded if they did not attend all training sessions or complete post-intervention assessment. |
| Alfarra et al., 2022 | Supervised PF rehabilitation (which included the PFM training, seven Yoga poses, manual therapy, and dilator) | There are a number of physical therapy approaches for cancer patients with sexual dysfunction combined with /without UI including PFM training, soft tissue mobilization, core strengthening abdominal and pelvic exercises, and vaginal dilators. | Mirror, dilators, yoga mat | The patients were seen for 8 x 45-min sessions weekly, including 30-min of exercise (PF exercises and yoga poses: Mountain pose, Tree pose, standing forward bend pose, worrier pose, bridge pose, bound angle pose, and seated twist pose), 10-min of manual therapy for the PFMs, and 5-min teaching the patient how to use dilator education. Dilator use was encouraged at home 3-4 times per week. | Unclear  Researcher (can assume women’s health specialist therapist but not explicitly stated). | Supervised.  Assume individual, F2F though not clearly stated. | King Faisal Specialist Hospital and research centre | 8 x 45-min sessions (one per week for 8 weeks). Each session included 30-min PF exercises, 10-min manual therapy, and 5-min of dilator education. Dilator was encouraged at home 3-4 times per week. | Unclear  Therapist started using the smallest dilator in the kit and slowly increase the dilator size over time as the patient feels more comfortable. | NR | NR | NR |
| Colombage et al., 2023 | Telehealth PFM training | There is some evidence that women with breast cancer taking aromatase inhibitors have weak PFM strength and endurance, there may be a role for PF conservative therapies, such as PFM training to treat stress UI in women following treatment for breast cancer. | Access to internet/Zoom facilities and a mobile device (not provided), access to an app, femfit® biofeedback device. | Participants underwent a 12-week PFM training program using an intra-vaginal pressure biofeedback device. In the first telehealth session, participants learnt how to contract their PFMs, how to use the femfit® device, and how to complete their home exercise program. The pressure readings from the femfit® were displayed via an app on their mobile phone. They followed a home exercise program installed on the femfit® phone app which was based on a published PFM training program. | All sessions of the program were conducted by a female researcher, a registered physiotherapist, who had undergone postgraduate PF physiotherapy training to enable her to deliver this intervention. | Supervised, individual, telehealth sessions via Zoom and weekly check-ins with the physiotherapist via email. | Zoom/participants home | 8 supervised sessions over 12 weeks. Participants completed 3 sets of 6-10 maximal contractions, 6-10 fast contractions, 3 podium (endurance) contractions and 3 knack contractions per PFM training session. Participants were instructed to complete the PFM training program 5 times per week. | The program progressed every 4 weeks by either increasing the duration of each contraction or increasing the load of the exercise by progressing from across-gravity (lying) to against-gravity (sit or stand) positions. The program was tailored by varying the home exercise program progression earlier or later than the set 4-week interval. | NR | Participant adherence to the exercise program was monitored through an exercise diary incorporated into the femfit® phone app which acted as a motivation strategy. Other motivation strategies included weekly check-ins with the physiotherapist which covered education on how PFM training may help their symptoms, setting short term goals, exploring enablers and barriers to completing their exercises and setting reminder notifications from the femfit® phone app to complete their home exercise program. | The mean attendance rate to supervised sessions with the physiotherapist was 95.9% (SD ± 3.1). The mean adherence rate to the home exercise program was 76.3% (SD ± 11.4). |
| Juraskova et al., 2013 | Olive oil, vaginal exercise, and moisturizer | Combined use of PFM relaxation exercises with olive oil as lubricant and a polycarbophil-based vaginal moisturizer may be effective in managing dyspareunia and sexual problems in women with breast cancer; however, this combination of treatments has not yet been investigated. | Replens (moisturizer), organic olive oil, nonlatex condoms | Participants attended 4 appointments with a PF physiotherapist who taught techniques for penetration to achieve PFM relaxation, and PFM relaxation exercises which involved 5 repetitions of gently contracting, holding, and relaxing the PFMs. In addition, participants were provided with Replens® and organic olive oil and instructed to: (i) apply Replens® 3 times per week; (ii) perform PFM relaxation exercises 2 times per day; and (iii) use olive oil during intercourse. | Experienced PF physiotherapist | F2F, individual, supervised for PFM training sessions and follow-up. Unsupervised for home-based activities. | NR  Assume in-clinic at the recruiting hospital for PFM training sessions and follow-up, and home-base otherwise though not clearly stated. | 26 weeks; PFM relaxation training was administered at weeks 0 and 4, with follow-up at weeks 12 and 26. Participants were instructed to PFM relaxation exercises 2 times per day, apply vaginal moisturizer 3 times per week, and use olive oil as a lubricant during intercourse. | NR | NR | Participants recorded their practice of the exercises and use of Replens® in a compliance diary which was reviewed, and any problems addressed, at each physiotherapy visit. | The average compliance with twice/day PFM exercises was 80%, and the average compliance with using Replens® three times/week was 88%, over the 26 weeks. |
| Bokaie et al., 2022 | Group counseling based on a problem-solving solution | The advantage of the problem-solving method is that it can be used for individual and/or group treatments. This approach is based on CBT and could improve an individual’s ability to cope with stressful life experiences. | NR | 8 x 90-min counseling session per week (assume language barrier - and assume intervention is 8 weeks in duration with 1 x 90-min counseling session per week). Participants formed 4 groups, each consisting of 8 individuals. Sessions included dedicating time and space to oneself, practicing relaxation skills, practicing sensory exercises, breathing training, practicing joint massage, and Kegel exercising and sharing it with one’s spouse. | NR | Group-based, in person | Breast cancer institute | 8 x 90-min counseling sessions (1 per week for 8 weeks) | NR | NR | NR | NR |
| Bober et al., 2019 | Sexual health and rehabilitation after ovarian suppression treatment | Previously developed a brief, evidence-based sexual health intervention for women treated for ovarian cancer and for women who underwent prophylactic oophorectomy. Guided by our integrative model for sexual health rehabilitation that addresses physical, psychological, and cognitive aspects of sexual dysfunction, we have expanded on this clinical intervention to test its efficacy in young breast cancer survivors suffering from ovarian suppression related sexual dysfunction. | Take-home materials were provided, including instructions for exercises, and information about personal products and relevant resources. | A single 4-hour group session, followed by a brief booster telephone call 1 month later. The intervention was comprised of 4 modules: education about sexual health rehabilitation; experiential exercises in body awareness, including PF relaxation; structured mindfulness based cognitive exercises, and: individual goal planning, with actionable steps to be reviewed during the post-group telephone booster. | Clinical psychologist experienced in treating cancer-related sexual dysfunction | Group-based and telephone booster (assume individual) | NR | 1 x 4-hour group session; 1 x telephone booster at 1-month. | Individual goal planning, with actionable steps to be reviewed during the post-group telephone booster. Booster phone call: intended to help women review their progress with their individualized action plan, problem-solve around continuing issues, and plan for maintenance moving forward. | NR | Booster telephone call 1-month post | NR |
| Schover et al., 2013 | Internet-based intervention for cancer-related sexual problems + 3 supplemental in-person counseling sessions | An internet-based intervention may be a cost-effective way for oncology settings to comply with the new guidelines. An internet-based intervention with couples after prostate cancer using email contact with the therapist was as effective in improving sexual function as 3 in-person sessions of CBT. Pilot studies with internet-based interventions for female sexual dysfunction have showed promise for women unselected for health and in gynecologic cancer survivors. | *Tendrils* website, therapist manual | Internet-based intervention: the *Tendrils* web site included text, graphics, animations, and multicultural photographs and clipart. Instructions suggested an order for using the site, but women could navigate from the home page to sections including information on sexual and fertility consequences of cancer and treatment; genital anatomy, sex after menopause; vaginal dryness and pain (with detailed advice on vaginal moisturizers, lubricants, PF exercises, and dilators); causes and treatment options for loss of desire or orgasm problems, body image, resuming sex comfortably using sensate focus exercises, sexual issues related to ostomies or incontinence, communication with sexual partners and health professionals, dating, lesbian relationships, and sex after childhood and adolescent cancer.  Internet-based intervention + 3 supplemental in-person counseling sessions: 3 supplemental in-person counseling sessions. A therapist manual provided overall guidance and content checklists for each of the 3 sessions. Counsellors guided women through the web site and discussed behavioral homework. | In-person CBT: 2 master’s level mental health professional | Individual, website.  CBT: supervised, F2F (assume individual). | Unclear | All women used the website for a 12-week period (half received 3 supplemental counseling sessions). | Generic for website.  Unclear for counseling sessions. | NR | Website usage was electronically recorded, excluding time spent completing questionnaires. For each participant, total min of usage was calculated during the 12-week treatment period, between post-treatment and 6-month follow-up assessments, and across the entire study period. | The treatment groups did not differ significantly on usage during the treatment period. Usage across the entire study was very similar between groups, with the mean (SD) for the combined sample at 149.0 (157.1). |

CBT: cognitive behavioral therapy, F2F: face-to-face, NR: not reported, PF: pelvic floor, PFM: pelvic floor muscle
