# Supplementary material for: Effectiveness of Pelvic Floor Muscle and Education-based Therapies on Bladder, Bowel, Vaginal, Sexual, Psychological Function, Quality of Life, and Pelvic Floor Muscle Function in Females Treated for Breast Cancer: A Systematic Review
Source: Curr Oncol Rep. 2025 Jan 29;27(2):168–89. doi: 10.1007/s11912-024-01633-3 (PMC11861006; doi:10.1007/s11912-024-01633-3)
Supplement: Supplementary file 3 — Supplementary file3 (DOCX 122 KB) [file 11912_2024_1633_MOESM3_ESM.docx]

**Supplementary Information 3. Effectiveness of Conservative Therapies in Breast Cancer Populations.**

(a) Bladder Function

| **Bladder function** | **Intervention arms** | | **Outcomes** | | **Results** | | **Considerations for interpretation** | | |
| --- | --- | --- | --- | --- | --- | --- | --- | --- | --- |
| **Study; sample size; type of cancer n (%)** | **Treatment group (TG)** | **Comparator group (CG)** | **What was measured?** | **Outcome measure/tool used** | **Values, significance; or narrative description** | **Direction of findings** | **Sample size calculation, primary outcome, end timepoint specified (if >1 follow-up timepoints); baseline outcome measure variance; floor/ceiling effect bias; adjustment for between-group differences on key characteristics and baseline outcomes** | **Adequate statistical analysis (ITT, per protocol, both, or unclear; multiple comparisons without adjustment)** | **TIDieR score (range 0-12); quality of intervention (dose response issues); interpretation of intervention effect (in relation to timing of delivery and of outcome assessment)** |
| **2 RCTs, between-group comparisons** | | | | | | | | | |
| Duijts 2012 [22];  n=422;  Breast (100%) | Education ± physical exercise:  (1) CBT (+ relaxation exercises); (3) CBT (+ relaxation exercises) + physical exercise | Wait-list (usual care) | Urinary symptoms | BFLUTS | Mean(SE) between-group differences:  Baseline to post-intervention and to 3 months post-intervention: TG (CBT) -1.10(0.30), p<0.001* and -1.09(0.40), p=0.007*; TG (CBT + physical exercise) -0.98(0.30), p=0.001* and -0.85(0.40), p=0.036* | Difference in favor for both TG (CBT and CBT + physical exercise) versus comparator | Sample size calculation provided (90% power to detect an effect size of 0.4); Primary outcome specified: endocrine symptoms (FACT-ES) and perceived burden of hot flashes and night sweats (Hot Flush Rating Scale) | Both;  Multiple comparisons without adjustment | 11;  Potentially adequate dosage; Short- and medium-term follow-up; Time since cancer treatment NR |
| Schover 2011 [23];  n=297;  Breast (100%) | Education (Sisters Peer Counseling in reproductive issues after treatment program) | Workbook +  optional telephone counseling of less than 30 min | Urinary incontinence | Breast Cancer Prevention Trial Symptom Checklist (5-point scale from not at all to extremely bothersome over the past 4 weeks) | No data provided, but statement of no differences between groups across time. | No differences between groups | No sample size calculation provided; No primary outcome specified; Unclear risk of baseline outcome variance and risk of floor/ceiling effect bias (no data provided) | ITT;  Multiple comparisons without adjustment | 11;  Potentially low dosage, mismatch between intervention and outcome, and similarities between intervention arms;  Short-, medium- and long-term follow-up; Time since cancer treatment NR |
| **0 non-RCT with 2 groups, between-group comparisons** | | | | | | | | | |
| **2 non-RCTs with single group, within-group comparisons** | | | | | | | | | |
| Alfarra 2022 [26];  n=30;  Breast (100%) | PFM therapy (active + passive) + yoga | NA | Urogenital distress | UDI-6 (from PFDI-20) | Results presented in multiple figures corresponding to the items of UDI-6 with the following statement: “Significant improvements in UDI-6 were observed in all participants by 80%,” p=NR. | Improvement in outcome | No sample size calculation provided;  Primary outcome specified: sexual function (FSFI-Breast Cancer); Unclear risk of baseline outcome variance and risk of floor/ceiling effect bias (no data provided) | Unclear;  Multiple comparisons without adjustment | 7;  Unclear dosage of intervention, Medium-term follow-up; Time since cancer treatment NR |
| Colombage 2023 [27];  n=54;  Breast (100%) | PFM therapy (active) | NA | Urinary incontinence prevalence; frequency, severity; impact | %; ICIQ-UI SF | Urinary incontinence prevalence: baseline 55/55 (100%) and post-intervention 32/55 (58%), p=NR;  Mean(95%CI) change:  ICIQ-UI SF: -9.4(-8.5 to -10.4), p=NR | Improvement in all outcomes | Sample size calculation provided, but not for this outcome – based on feasibility outcomes and sample size achieved | ITT;  Multiple comparisons without adjustment | 11;  Short-term follow-up; Time since cancer treatment NR |
| BFLUTS: Bristol Female Lower Urinary Tract Symptoms Questionnaire, CBT: cognitive-behavioral therapy, CG: comparator group, CI: confidence interval, FACT-ES: Functional Assessment of Cancer Therapy-Endocrine Subscale, FSFI: Female Sexual Function Index, ICIQ-UI SF: International Consultation on Incontinence Questionnaire-Urinary Incontinence Short Form, ITT: intention-to-treat, PFDI-20: Pelvic Floor Distress Inventory-20, NA: not applicable, NR: not reported, PFM: pelvic floor muscle, RCT: randomized controlled trial, TG: treatment group, UDI-6: Urinary Distress Inventory-6  *Difference reached statistical significance (p<0.05) or reached statistical significance according to methods used by the authors. | | | | | | | | | |

(b) Vaginal Function

| **Vaginal function** | **Intervention arms** | | **Outcomes** | | **Results** | | **Considerations for interpretation** | | |
| --- | --- | --- | --- | --- | --- | --- | --- | --- | --- |
| **Study; sample size; type of cancer n (%)** | **Treatment group (TG)** | **Comparator group (CG)** | **What was measured?** | **Outcome measure/tool used** | **Values, significance; or narrative description** | **Direction of findings** | **Sample size calculation, primary outcome, end timepoint specified (if >1 follow-up timepoints); baseline outcome measure variance; floor/ceiling effect bias; adjustment for between-group differences on key characteristics and baseline outcomes** | **Adequate statistical analysis (ITT, per protocol, both, or unclear; multiple comparisons without adjustment)** | **TIDieR score (range 0-12); quality of intervention (dose response issues); interpretation of intervention effect (in relation to timing of delivery and of outcome assessment)** |
| **2 RCTs, between-group comparisons** | | | | | | | | | |
| Advani 2017 [18];  n=57;  Breast (100%) | PFM therapy (passive) + education (+ usual care) | Usual care | Vaginal irritation; vaginal pH | BESS - gynecologic irritation subscale; Vaginal pH | BESS - gynecologic irritation subscale at 6 months and 12 months post-intervention: not significantly  different across time or between groups;  Vaginal pH at 6 months and 12 months post-intervention:  not significantly  different across time or between groups. | No differences between groups | No sample size calculation provided; Primary outcome specified: sexual function (FSFI); Potential risk of baseline outcome variance | ITT;  Multiple comparisons without adjustment | 9;  Unclear dosage of therapy and no data provided for adherence to recommendations; Medium- and long-term follow-up; Time since cancer treatment NR |
| Schover 2011 [23];  n=297;  Breast (100%) | Education (Sisters Peer Counseling in reproductive issues after treatment program) | Workbook +  optional telephone counseling of less than 30 min | Vaginal irritation | Breast Cancer Prevention Trial Symptom Checklist (5-point scale from not at all to extremely bothersome over the past 4 weeks) | - | Not interpretable | No sample size calculation provided; No primary outcome specified; Unclear risk of baseline outcome variance and risk of floor/ceiling effect bias (no data provided) | ITT;  Multiple comparisons without adjustment | 11;  Short-, medium- and long-term follow-up; Time since cancer treatment NR |
| **0 non-RCT with 2 groups, between-group comparisons** | | | | | | | | | |
| **0 non-RCT with single group, within-group comparisons** | | | | | | | | | |
| BESS: Breast Cancer Prevention Trial Symptom Scale, CG: comparator group, CI: confidence interval, FSFI: Female Sexual Function Index, ITT: intention-to-treat, NR: not reported, PFM: pelvic floor muscle, RCT: randomized controlled trial, TG: treatment group  *Difference reached statistical significance (p<0.05) or reached statistical significance according to methods used by the authors. | | | | | | | | | |

(c) Sexual Function

| **Sexual function** | **Intervention arms** | | **Outcomes** | | **Results** | | **Considerations for interpretation** | | |
| --- | --- | --- | --- | --- | --- | --- | --- | --- | --- |
| **Study; sample size; type of cancer n (%)** | **Treatment group (TG)** | **Comparator group (CG)** | **What was measured?** | **Outcome measure/tool used** | **Values, significance; or narrative description** | **Direction of findings** | **Sample size calculation, primary outcome, end timepoint specified (if >1 follow-up timepoints); baseline outcome measure variance; floor/ceiling effect bias; adjustment for between-group differences on key characteristics and baseline outcomes** | **Adequate statistical analysis (ITT, per protocol, both, or unclear; multiple comparisons without adjustment)** | **TIDieR score (range 0-12); quality of intervention (dose response issues); interpretation of intervention effect (in relation to timing of delivery and of outcome assessment)** |
| **6 RCTs, between-group comparisons** | | | | | | | | | |
| Advani 2017 [18];  n=57;  Breast (100%) | PFM therapy (passive) + education (+ usual care) | Usual care | Sexual function; Sexual interest; Sexual distress; Dyspareunia | FSFI; MSIQ; FSDS-R; BESS - dyspareunia subscale | FSFI (total score and pain subscale) and MSIQ (total score, desire, response, satisfaction subscales) at 6 months and 12 months post-intervention: not significantly  different across time or between groups, except active group H (hyaluronic acid moisturizer) improved significantly more on sexual function total score than group P (prebiotic moisturizer), p=0.04*;  Mean(SD):  FSDS-R at 6 months (12 months NR) post-intervention: TG 12.92(11.78) vs CG 23.18(16.50), p=0.02*;  BESS - dyspareunia subscale at 6 months (12 months NR) post-intervention: TG 2.96(2.30) vs CG 3.82(2.88), p=0.07 | Mixed;  Difference in favor of TG on FSDS-R;  No differences between groups on FSFI, MSIQ, BESS - dyspareunia subscale | No sample size calculation provided; Primary outcome specified: sexual function (FSFI); Potential risk of baseline outcome variance | ITT;  Multiple comparisons without adjustment | 9;  Unclear dosage of therapy and no data provided for adherence to recommendations; Medium- (and long-term for some outcomes) follow-up; Time since cancer treatment NR |
| Fatehi 2019 [19];  n=118;  Breast (100%) | Education (psychosexual counselling) | Wait-list (no intervention) | Sexual function; Sexual satisfaction; Sexual quality of life | FSFI; LSSQ; SQOL-F | Mean(SD) at 3 months post-intervention (no data provided for post-intervention):  FSFI: TG 21.49(6.7) vs CG 14.10(8.1), p<0.001*;  LSSQ: TG 44.89(6.20) vs CG 46.20(4.0), p=0.073;  SQOL-F: TG 91.01(17.9) vs CG 38.23(10.6), p<0.001* | Mixed;  Difference in favor of TG for sexual function and sexual quality of life;  No differences between groups for sexual satisfaction | Sample size calculation provided (no detail on which outcome and follow-up timepoint were selected as primary); Sample size not achieved | ITT;  Multiple comparisons without adjustment | 4;  Potentially adequate dosage; Medium-term follow-up only; Time since cancer treatment: mean 10-11 (SD 4-5) in months |
| Hummel 2017, 2018 [20, 21];  n=169;  Breast (100%) | Education (internet-based CBT) | Wait-list (with information) | Sexual function; Sexual distress | FSFI; SAQ; FSDS-R | Mean(SD) between-group differences -baseline to post-intervention:  FSFI total score: 3.84(1.45), p=0.009*;  FSFI desire: 0.66(0.15), p<0.001*;  FSFI arousal: 0.88(0.29), p=0.002*;  FSFI lubrication: 0.95(0.32), p=0.004*;  FSFI orgasm: 0.74(0.35), p=0.035*;  FSFI satisfaction: 0.42(0.25), p=0.095;  FSFI pain: 0.39(0.34), p=0.254;  SAQ pleasure: 2.41(0.64), p<0.001*;  SAQ discomfort: -1.03(0.31), p=0.001*;  SAQ habit: 0.46(0.19), p=0.017*;  FSDS-R: -5.00(1.39), p<0.001*  Linear and quadratic effect of time from baseline to post-intervention, 3 months and 9 months post-intervention TG within-group differences: data from different timepoints were combined to compare between 2 timelines, i.e., baseline, follow-up timepoints 1-2 vs follow-up timepoints 2-3-4. | Mixed;  Between-group comparisons:  Difference in favor of TG for FSFI sexual function, desire, arousal, lubrication, SAQ pleasure, discomfort, habit, and FSDS-R;  No differences between groups for FSFI orgasm, satisfaction, and pain  TG within-group comparisons:  Not interpretable according to follow-up timepoints | Sample size calculation provided (80% power to detect an effect size of 0.5); Primary outcomes specified: Sexual function (FSFI and SAQ) and sexual distress (FSDS-R); Significant difference between groups at baseline on proportion of females who had chemotherapy (greater proportion in CG) – not accounted for in analysis | ITT;  Multiple comparisons without adjustment | 11;  Potentially adequate dosage but data on adherence appear to indicate low to moderate participant adherence for this therapy of 20 sessions in 20-24 weeks; Short-term follow-up for between-group comparison and medium-term follow-up for TG within-group comparison; Time since cancer treatment NR;  Time since cancer diagnosis: mean 38 (SD 16) in months |
| Duijts 2012 [22];  n=422;  Breast (100%) | Education ± physical exercise:  (1) CBT (+ relaxation exercises); (3) CBT (+ relaxation exercises) + physical exercise | Wait-list (usual care) | Sexual function | SAQ | Mean(SE) between-group differences -baseline to post-intervention and to 3 months post-intervention:  SAQ habit: TG (CBT) 0.24(0.16), p=0.134 and 0.33(0.16), p=0.042*; TG (CBT + physical exercise) 0.12(0.16), p=0.443 and 0.51(0.16), p=0.002*;  SAQ pleasure: TG (CBT) 2.04(0.65), p=0.002* and 1.66(0.71), p=0.022*; TG (CBT + physical exercise) 1.28(0.76), p=0.095 and 1.52(0.79), p=0.056 | Mixed (versus comparator);  Difference in favor of TG for sexual habit (CBT and CBT + physical exercise for medium-term follow-up only);  Difference in favor of TG for sexual pleasure (CBT only for short- and medium-term follow-up) | Sample size calculation provided (90% power to detect an effect size of 0.4); Primary outcome specified: endocrine symptoms (FACT-ES) and perceived burden of hot flashes and night sweats (Hot Flush Rating Scale) | Both;  Multiple comparisons without adjustment | 11;  Potentially adequate dosage but high level of undercompliance reported;  Short- and medium-term follow-up; Time since cancer treatment NR |
| Schover 2011 [23];  n=297;  Breast (100%) | Education (Sisters Peer Counseling in reproductive issues after treatment program) | Workbook +  optional telephone counseling of less than 30 min | Sexual function | FSFI | No data provided, but statement of no differences between groups across time. | No differences between groups | No sample size calculation provided; No primary outcome specified; Unclear risk of baseline outcome variance and risk of floor/ceiling effect bias (no data provided) | ITT;  Multiple comparisons without adjustment | 11;  Potentially low dosage and similarities between intervention arms;  Short-, medium- and long-term follow-up; Time since cancer treatment NR |
| Schover 2013 [24];  n=58;  Breast (81%)  Gynecological (19%) | Education (internet-based intervention for cancer-related sexual problems + 3 supplemental in-person counselling sessions) | Internet-based intervention for cancer-related sexual problems | Sexual function; Sexual interest | FSFI; MSIQ | No data provided, but statement of difference in favor of TG. | Difference in favor of TG | No sample size calculation provided; Primary outcome specified: sexual function (FSFI); Unclear risk of baseline outcome variance and floor/ceiling effect bias (no data provided) | ITT;  Multiple comparisons without adjustment | 10;  Short- and medium-term follow-up; Time since cancer treatment: NR;  Time since cancer diagnosis: mean 3.5 (SD 4) in years |
| **1 non-RCT with 2 groups, between-group comparisons** | | | | | | | | | |
| Zangeneh 2023 [25];  n=80;  Breast (100%) | Education (sexual education based on the Ex‑PLISSIT model including relaxation techniques, breathing techniques, PFM exercises) | Routine care | Sexual function; Sexual satisfaction | FSFI; LSSQ | Mean±SD:  FSFI (total score) at post-intervention: TG 60.5±17.97 vs CG 48.7±20.18, p=0.176;  LSSQ at post-intervention: TG 97.9±12.65 vs CG  86.2±14.35, p=0.020* | Mixed;  Improvement in sexual satisfaction;  No improvement in sexual function | Sample size formula provided (no detail on which outcome was selected as primary); Unclear risk of baseline outcome variance and floor/ceiling effect bias (data provided don’t match the score range of FSFI) | Per protocol (only those who attended all sessions were included in the analysis);  Multiple comparisons without adjustment | 10;  Potentially adequate dosage;  Short-term follow-up; Time since cancer diagnosis/treatment NR |
| **4 non-RCTs with single group, within-group comparisons** | | | | | | | | | |
| Alfarra 2022 [26];  n=30;  Breast (100%) | PFM therapy (active + passive) + yoga | NA | Sexual function | FSFI-Breast Cancer | Results presented in multiple figures corresponding to the items of FSFI-Breast Cancer with the following statement: “Significant improvement in sexual function as assessed by the FSFI-BC indicated that sex desire improved by 86%, sexual arousal improved by 83%, lubrication improved by 76.3%, orgasm improved by 70%, general satisfaction improved by 44-54%, 73.3% felt pain minor, and  23% free of pain during intercourse.” | Improvement in all outcomes | No sample size calculation provided;  Primary outcome specified: sexual function (FSFI-Breast Cancer); Unclear risk of baseline outcome variance and risk of floor/ceiling effect bias; Unstandardized data interpretation and unclear for level of significance (no data of statistical analysis provided) | Unclear;  Multiple comparisons without adjustment | 7;  Unclear dosage of intervention, Medium-term follow-up; Time since cancer treatment NR |
| Juraskova 2013 [28];  n=25;  Breast (100%) | PFM therapy (active + passive) | NA | Sexual satisfaction; Dyspareunia; Sexual function | FSFI satisfaction subscale; VAS-DYS; SAQ | Mean(SD):  FSFI sexual satisfaction: baseline 2.4(1.37) and post-intervention 3.5(1.40), p=significant;  VAS-DYS: baseline 7.0(2.40) and post-intervention 2.7(2.31), p=significant;  SAQ total score: baseline 7.2(3.19) and post-intervention 11.6(4.26), p=significant;  SAQ pleasure: baseline 6.4(3.43) and post-intervention 8.7(3.04), p=significant;  SAQ discomfort: baseline 0.8(1.00) and post-intervention 2.9(2.05), p=significant;  SAQ habit: baseline 0.6(0.62) and post-intervention 0.9(0.80), p=NS | Mixed;  Improvement in all outcomes, expect SAQ habit | No sample size calculation provided; No primary outcome specified | ITT;  Multiple comparisons without adjustment | 9;  Potentially adequate dosage;  Short-term follow-up; Time since cancer treatment NR |
| Bokaie 2022 [29];  n=32;  Breast (100%) | Education (group counselling based on a problem-solving solution) | NA | Sexual function; Sexual satisfaction | FSFI; LSSQ | Mean(SD):  FSFI total score: baseline 18.37(8.35), post-intervention 20.88(7.67), and 1 month post-intervention 22.95(5.79), p<0.001*;  FSFI desire: baseline 2.98(0.67), post-intervention 2.99(0.68), and 1 month post-intervention 3.11(0.71), p<0.001*;  FSFI arousal: baseline 2.98(1.30), post-intervention 3.22(1.04), and 1 month post-intervention 4.43(0.98), p<0.003*;  FSFI lubrication: baseline 2.91(1.47), post-intervention 3.36(1.32), and 1 month post-intervention 4.02(0.99), p<0.001*;  FSFI orgasm: baseline 3.24(1.78), post-intervention 3.81(1.36), and 1 month post-intervention 4.08(0.96), p<0.001*;  FSFI satisfaction: baseline 3.39(1.67), post-intervention 3.90(1.54), and 1 month post-intervention 4.07(1.02), p<0.001*;  FSFI pain: baseline 3.18(2.08), post-intervention 3.60(1.73), and 1 month post-intervention 3.24(1.14), p<0.001*;  LSSQ: baseline 65.27(5.98), post-intervention 68.08(5.61), and 1 month post-intervention 70.46(5.35), P<0.02* | Improvement in all outcomes | No sample size calculation provided; No primary outcome specified | ITT;  Multiple comparisons without adjustment, except Bonferroni post-hoc test for FSFI total score | 5;  Short- and medium-term follow-up; Time since cancer treatment: mean 3.5 (SD 3) in years |
| Bober 2020 [30];  n=20;  Breast (100%) | Education (sexual health and rehabilitation after ovarian suppression treatment) | NA | Sexual function | FSFI | Mean(SD) change from baseline to 2 months post-intervention:  FSFI total: 5.24(9.01), p=0.021*;  FSFI desire: 0.66(1.00), p=0.010*;  FSFI arousal: 0.63(1.74), p=0.131;  FSFI lubrication: 1.15(1.60), p=0.006*;  FSFI orgasm: 1.05(2.08), p=0.040*;  FSFI satisfaction: 1.12(1.89), p=0.019*;  FSFI pain: 0.63(2.40), p=0.267 | Mixed;  Improvement in all outcomes, except arousal and pain | No sample size calculation provided; No primary outcome specified | ITT;  Multiple comparisons without adjustment | 9;  Potentially adequate dosage; Medium-term follow-up; Time since cancer treatment: 85% at 1 month or less and 15% at 2-3 months post-cancer treatment |
| BESS: Breast Cancer Prevention Trial Symptom Scale, CBT: cognitive-behavioral therapy, CG: comparator group, CI: confidence interval, FACT-ES: Functional Assessment of Cancer Therapy-Endocrine Subscale, FSDS(-R): Female Sexual Distress Scale(-Revised), FSFI: Female Sexual Function Index, ITT: intention-to-treat, LSSQ: Larson Sexual Satisfaction Questionnaire, MSIQ: Menopausal Sexual Interest Questionnaire, NA: not applicable, NR: not reported, NS: non-significant, PFM: pelvic floor muscle, RCT: randomized controlled trial, SAQ: Sexual Activity Questionnaire, SD: standard deviation, SQOL-F: Sexual Quality of Life-Female Questionnaire, TG: treatment group, VAS-DYS: Visual Analogue Score-Dyspareunia  *Difference reached statistical significance (p<0.05) or reached statistical significance according to methods used by the authors. | | | | | | | | | |

(d) Psychological Function

| **Psychological function** | **Intervention arms** | | **Outcomes** | | **Results** | | **Considerations for interpretation** | | |
| --- | --- | --- | --- | --- | --- | --- | --- | --- | --- |
| **Study; sample size; type of cancer n (%)** | **Treatment group (TG)** | **Comparator group (CG)** | **What was measured?** | **Outcome measure/tool used** | **Values, significance; or narrative description** | **Direction of findings** | **Sample size calculation, primary outcome, end timepoint specified (if >1 follow-up timepoints); outcome heterogeneity; floor/ceiling effect bias; adjustment for between-group differences on key characteristics and baseline outcomes** | **Adequate statistical analysis (ITT, per protocol, both, or unclear; multiple comparisons without adjustment)** | **TIDieR score (range 0-12); quality of intervention (dose response issues); interpretation of intervention effect (in relation to timing of delivery and of outcome assessment)** |
| **4 RCTs, between-group comparisons** | | | | | | | | | |
| Hummel 2017, 2018 [20, 21];  n=169;  Breast (100%) | Education (internet-based CBT) | Wait-list (with information) | Anxiety; Depression | HADS | Mean(SD) between-group differences -baseline to post-intervention:  HADS depression: -0.18(0.48), p=0.707;  HADS anxiety: -0.17(0.51), p=0.738;  HADS psychological distress: -0.35(0.88), p=0.690 | No differences between groups | Sample size calculation provided (80% power to detect an effect size of 0.5); Primary outcomes specified: Sexual function (FSFI and SAQ) and sexual distress (FSDS-R); Potential risk of floor/ceiling effect bias (low scores at baseline); Significant difference between groups at baseline on proportion of females who had chemotherapy (greater proportion in CG) – not accounted for in analysis | ITT;  Multiple comparisons without adjustment | 11;  Potentially adequate dosage but data on adherence appear to indicate low to moderate participant adherence for this therapy of 20 sessions in 20-24 weeks; Short-term follow-up for between-group comparison; Time since cancer treatment NR;  Time since cancer diagnosis: mean 38 (SD 16) in months |
| Duijts 2012 [22];  n=422;  Breast (100%) | Education ± physical exercise:  (1) CBT (+ relaxation exercises); (3) CBT (+ relaxation exercises) + physical exercise | Wait-list (usual care) | Psychological distress | HADS | - | Not interpretable | Sample size calculation provided (90% power to detect an effect size of 0.4); Primary outcome specified: endocrine symptoms (FACT-ES) and perceived burden of hot flashes and night sweats (Hot Flush Rating Scale) | Both;  Multiple comparisons without adjustment | 11;  Short- and medium-term follow-up; Time since cancer treatment NR |
| Schover 2011 [23];  n=297;  Breast (100%) | Education (Sisters Peer Counseling in reproductive issues after treatment program) | Workbook +  optional telephone counseling of less than 30 min | Emotional distress | BSI-18 | No data provided, but statement of no differences between groups across time. | No differences between groups | No sample size calculation provided; No primary outcome specified; Unclear risk of baseline outcome variance and risk of floor/ceiling effect bias (no data provided) | ITT;  Multiple comparisons without adjustment | 11;  Potentially low dosage, and similarities between intervention arms; Short-, medium- and long-term follow-up; Time since cancer treatment NR |
| Schover 2013 [24];  n=58;  Breast (81%)  Gynecological (19%) | Education (internet-based intervention for cancer-related sexual problems + 3 supplemental in-person counselling sessions) | Internet-based intervention for cancer-related sexual problems | Emotional distress | BSI-18 | No data provided, but statement of no differences between groups across time. | No differences between groups | No sample size calculation provided; Primary outcome specified: sexual function (FSFI); Unclear risk of baseline outcome variance and floor/ceiling effect bias (no data provided) | ITT;  Multiple comparisons without adjustment | 10;  Potentially low dosage or mismatch between intervention and outcome;  Short- and medium-term follow-up; Time since cancer treatment: NR;  Time since cancer diagnosis: mean 3.5 (SD 4) in years |
| **0 non-RCT with 2 groups, between-group comparisons** | | | | | | | | | |
| **2 non-RCTs with single group, within-group comparisons** | | | | | | | | | |
| Juraskova 2013 [28];  n=25;  Breast (100%) | PFM therapy (active + passive) | NA | Psychological distress | HADS | HADS anxiety: baseline 6.8(4.55) and post-intervention 6.0(4.28), p=significant;  HADS depression: baseline median 2 (IQR 1–4) and post-intervention median 1 (IQR range 1–4), p=NS | Mixed;  Improvement in anxiety but not in depression | No sample size calculation provided; No primary outcome specified; Potential risk of floor/ceiling effect bias | ITT;  Multiple comparisons without adjustment | 9;  Potentially adequate dosage; Short-term follow-up; Time since cancer treatment NR |
| Bober 2020 [30];  n=20;  Breast (100%) | Education (sexual health and rehabilitation after ovarian suppression treatment) | NA | Somatization; Depression; Anxiety; Global severity | BSI-18 | Mean(SD) change from baseline to 2 months post-intervention:  Somatization: 0.05(6.79), p=0.973;  Depression: 3.05(6.92), p=0.070;  Anxiety: 3.53(5.47), p<0.001*;  Global severity: 3.53(5.47), p=0.012* | Mixed;  Improvement in anxiety and global severity;  No improvement in somatization and depression | No sample size calculation provided; No primary outcome specified | ITT;  Multiple comparisons without adjustment | 9;  Potentially adequate dosage; Medium-term follow-up; Time since cancer treatment: 85% at 1 month or less and 15% at 2-3 months post-cancer treatment |
| BSI-18: Brief Symptom Inventory-18, CBT: cognitive-behavioral therapy, CG: comparator group, CI: confidence interval, FACT-ES: Functional Assessment of Cancer Therapy-Endocrine Subscale, FSFI: Female Sexual Function Index, FSDS(-R): Female Sexual Distress Scale(-Revised), HADS: Hospital Anxiety and Depression Score, IQR: interquartile range, ITT: intention-to-treat, NA: not applicable, NR: not reported, NS: non-significant, PFM: pelvic floor muscle, RCT: randomized controlled trial, SAQ: Sexual Activity Questionnaire, SD: standard deviation, TG: treatment group  *Difference reached statistical significance (p<0.05) or reached statistical significance according to methods used by the authors. | | | | | | | | | |

(e) Quality of Life

| **Quality of life** | **Intervention arms** | | **Outcomes** | | **Results** | | **Considerations for interpretation** | | |
| --- | --- | --- | --- | --- | --- | --- | --- | --- | --- |
| **Study; sample size; type of cancer n (%)** | **Treatment group (TG)** | **Comparator group (CG)** | **What was measured?** | **Outcome measure/tool used** | **Values, significance; or narrative description** | **Direction of findings** | **Sample size calculation, primary outcome, end timepoint specified (if >1 follow-up timepoints); outcome heterogeneity; floor/ceiling effect bias; adjustment for between-group differences on key characteristics and baseline outcomes** | **Adequate statistical analysis (ITT, per protocol, both, or unclear; multiple comparisons without adjustment)** | **TIDieR score (range 0-12); quality of intervention (dose response issues); interpretation of intervention effect (in relation to timing of delivery and of outcome assessment)** |
| **4 RCTs, between-group comparisons** | | | | | | | | | |
| Hummel 2017, 2018 [20, 21];  n=169;  Breast (100%) | Education (internet-based CBT) | Wait-list (with information) | Health-related quality of life | SF-36; FACT-ES | Mean(SD) between-group differences -baseline to post-intervention:  SF-36 physical functioning: 0.27(1.82), p=0.881;  SF-36 role physical: -0.13(7.07), p=0.985;  SF-36 bodily pain: 1.53(3.26), p=0.639;  SF-36 general health perceptions: -0.16(2.46), p=0.949;  SF-36 vitality: -0.09(2.99), p=0.976;  SF-36 social functioning: 0.40(3.68), p=0.109;  SF-36 role emotional: -9.90(7.04), p=0.161;  SF-36 mental health: -2.06(2.62), p=0.433;  FACT-ES: 1.91(1.16), p=0.103 | No differences between groups | Sample size calculation provided (80% power to detect an effect size of 0.5); Primary outcomes specified: Sexual function (FSFI and SAQ) and sexual distress (FSDS-R); Significant difference between groups at baseline on proportion of females who had chemotherapy (greater proportion in CG) – not accounted for in analysis | ITT;  Multiple comparisons without adjustment | 11;  Potentially adequate dosage but data on adherence appear to indicate low to moderate participant adherence for this therapy of 20 sessions in 20-24 weeks; Short-term follow-up for between-group comparison; Time since cancer treatment NR;  Time since cancer diagnosis: mean 38 (SD 16) in months |
| Duijts 2012 [22];  n=422;  Breast (100%) | Education ± physical exercise:  (1) CBT (+ relaxation exercises); (3) CBT (+ relaxation exercises) + physical exercise | Wait-list (usual care) | Health-related quality of life | SF-36; FACT-ES | Mean(SE) between-group differences -baseline to post-intervention and to 3 months post-intervention:  SF-36 physical functioning: TG (CBT) 6.65(2.04), p=0.001* and 3.69(2.24), p=0.100; TG (CBT + physical exercise) 6.52(2.03), p=0.001* and 3.51(2.24), p=0.117;  SF-36 bodily pain: TG (CBT) -6.37(3.31), p=0.055 and 4.47(3.48), p=0.199; TG (CBT + physical exercise) -5.32(3.23), p=0.107 and -1.55(3.48), p=0.656;  SF-36 mental health: TG (CBT) 4.65(2.27), p=0.042* and 1.76(2.39), p=0.463; TG (CBT + physical exercise) 8.18(2.56), p=0.002* and 8.46(2.69), p=0.002*;  SF-36 mental component: TG (CBT) 3.83(1.56), p=0.015* and 1.09(1.74), p=0.532; TG (CBT + physical exercise) 5.49(1.77), p=0.002* and 4.65(1.97), p=0.019*;  SF-36 vitality: TG (CBT) 8.11(2.60), p=0.002* and 3.85(3.09), p=0.214; TG (CBT + physical exercise) 7.75(2.92), p=0.009* and 5.13(3.47), p=0.141;  SF-36 role emotional: TG (CBT) 12.96(7.20), p=0.073 and 5.73(8.25), p=0.488; TG (CBT + physical exercise) 26.53(8.14), p=0.001* and 19.99(9.30), p=0.033*;  FACT-ES: TG (CBT) 4.11(0.95), p<0.001* and 3.48(0.96), p<0.001*; TG (CBT + physical exercise) 3.51(0.95), p<0.001* and 3.14(0.96), p=0.001* | Mixed (versus comparator);  Difference in favor of both TGs for physical functioning and vitality at short-term follow-up only;  Difference in favor of TG (CBT + physical exercise only) for role emotional at short- and medium-term follow-up;  Difference in favor of both TGs for mental subscales at short-term follow-up but only for CBT + physical exercise at medium-term follow-up;  Difference in favor of both TG (CBT and CBT + physical exercise) for FACT-ES at short- and medium-term follow-up;  No differences between groups for bodily pain | Sample size calculation provided (90% power to detect an effect size of 0.4); Primary outcome specified: endocrine symptoms (FACT-ES) and perceived burden of hot flashes and night sweats (Hot Flush Rating Scale) | Both;  Multiple comparisons without adjustment | 11;  Potentially adequate dosage but high level of undercompliance reported;  Short- and medium-term follow-up; Time since cancer treatment NR |
| Schover 2011 [23];  n=297;  Breast (100%) | Education (Sisters Peer Counseling in reproductive issues after treatment program) | Workbook +  optional telephone counseling of less than 30 min | Spirituality well-being | FACIT-Sp | No data provided, but statement of no differences between groups across time. | No differences between groups | No sample size calculation provided; No primary outcome specified; Unclear risk of baseline outcome variance and risk of floor/ceiling effect bias (no data provided) | ITT;  Multiple comparisons without adjustment | 11;  Potentially low dosage and similarities between intervention arms; Short-, medium- and long-term follow-up; Time since cancer treatment NR |
| Schover 2013 [24];  n=58;  Breast (81%)  Gynecological (19%) | Education (internet-based intervention for cancer-related sexual problems + 3 supplemental in-person counselling sessions) | Internet-based intervention for cancer-related sexual problems | Quality of life | Quality of Life in Adult Cancer Survivors (QLACS) | No data provided, but statement of no differences between groups across time. | No differences between groups | No sample size calculation provided; Primary outcome specified: sexual function (FSFI); Unclear risk of baseline outcome variance and floor/ceiling effect bias (no data provided) | ITT;  Multiple comparisons without adjustment | 10;  Potentially low dosage;  Short- and medium-term follow-up; Time since cancer treatment: NR;  Time since cancer diagnosis: mean 3.5 (SD 4) in years |
| **0 non-RCT with 2 groups, between-group comparisons** | | | | | | | | | |
| **1 non-RCT with single group, within-group comparisons** | | | | | | | | | |
| Juraskova 2013 [28];  n=25;  Breast (100%) | PFM therapy (active + passive) | NA | Health-related quality of life | FACT-B; FACT-ES | Mean(SD):  FACT-B: baseline 108.7(13.68) and post-intervention 113.8(15.50), p=significant;  FACT-ES: baseline 51.0(9.24) and post-intervention 53.8(8.66), p=significant | Improvement in all outcomes | No sample size calculation provided; No primary outcome specified | ITT;  Multiple comparisons without adjustment | 9;  Short-term follow-up; Time since cancer treatment NR |
| CBT: cognitive-behavioral therapy, CG: comparator group, CI: confidence interval, FACIT-Sp: Spiritual Well Being Subscale of the Functional Assessment of Cancer, FACT-B: Functional Assessment of Cancer Therapy-Breast, FACT-ES: Functional Assessment of Cancer Therapy-Endocrine Subscale, FSFI: Female Sexual Function Index, FSDS(-R): Female Sexual Distress Scale(-Revised), ITT: intention-to-treat, NA: not applicable, NR: not reported, NS: non-significant, PFM: pelvic floor muscle, RCT: randomized controlled trial, SAQ: Sexual Activity Questionnaire, SD: standard deviation, TG: treatment group  *Difference reached statistical significance (p<0.05) or reached statistical significance according to methods used by the authors. | | | | | | | | | |

(f) PFM Function

| **PFM function** | **Intervention arms** | | **Outcomes** | | **Results** | | **Considerations for interpretation** | | |
| --- | --- | --- | --- | --- | --- | --- | --- | --- | --- |
| **Study; sample size; type of cancer n (%)** | **Treatment group (TG)** | **Comparator group (CG)** | **What was measured?** | **Outcome measure/tool used** | **Values, significance; or narrative description** | **Direction of findings** | **Sample size calculation, primary outcome, end timepoint specified (if >1 follow-up timepoints); outcome heterogeneity; floor/ceiling effect bias; adjustment for between-group differences on key characteristics and baseline outcomes** | **Adequate statistical analysis (ITT, per protocol, both, or unclear; multiple comparisons without adjustment)** | **TIDieR score (range 0-12); quality of intervention (dose response issues); interpretation of intervention effect (in relation to timing of delivery and of outcome assessment)** |
| **0 RCT, between-group comparisons** | | | | | | | | | |
| **0 non-RCT with 2 groups, between-group comparisons** | | | | | | | | | |
| **3 non-RCTs with single group, within-group comparisons** | | | | | | | | | |
| Alfarra 2022 [26];  n=30;  Breast (100%) | PFM therapy (active + passive) + yoga | NA | PFM strength | Oxford scale, via vaginal palpation | “Muscle power as measured by the Oxford scale improved from grade 1 or 2 out of 5 to 3 or 3+ out of 5”, p=NR. | Unclear (likely improvement in outcome) | No sample size calculation provided;  Primary outcome specified: sexual function (FSFI-Breast Cancer); Unclear risk of baseline outcome variance and risk of floor/ceiling effect bias (no data provided) | Unclear;  Multiple comparisons without adjustment | 7;  Unclear dosage of intervention, Medium-term follow-up; Time since cancer treatment NR |
| Colombage 2023 [27];  n=54;  Breast (100%) | PFM therapy (active) | NA | PFM strength | Intra-vaginal squeeze pressure (mmHg), via manometer | Mean(95%CI) change: 4.8(3.9 to 5.5), p=NR | Improvement in outcome | Sample size calculation provided, but not for this outcome – based on feasibility outcomes and sample size achieved | ITT;  Multiple comparisons without adjustment | 11;  Short-term follow-up; Time since cancer treatment NR |
| Juraskova 2013 [28];  n=25;  Breast (100%) | PFM therapy (active + passive) | NA | PFM resting; PFM relaxation; | Manometry (H_2_O); intra-vaginal surface EMG (μV) | Mean(SD):  PFM resting (H_2_O): baseline 33.7(8.47) and post-intervention 28.4(5.30), p=NR;  PFM resting (μV): baseline 1.6(0.48) and post-intervention 1.6(0.39), p=NR;  PFM relaxation (H_2_O): baseline 28.2(8.36) and post-intervention 22.8(6.88), p=NR;  PFM resting (μV): baseline 1.3(0.34) and post-intervention 1.2(0.20), p=NR | Unclear (potential improvement - reduction - in PFM resting and relaxation as assessed by manometry but not by surface EMG) | No sample size calculation provided; No primary outcome specified | ITT;  Multiple comparisons without adjustment | 9;  Potentially adequate dosage; Short-term follow-up; Time since cancer treatment NR |
| CG: comparator group, CI: confidence interval, EMG: electromyography, FSFI: Female Sexual Function Index, ITT: intention-to-treat, MOS : modified Oxford scale, NA: not applicable, NR: not reported, PFM: pelvic floor muscle, RCT: randomized controlled trial, SD: standard deviation, TG: treatment group  *Difference reached statistical significance (p<0.05) or reached statistical significance according to methods used by the authors. | | | | | | | | | |
